# Proceedings of the 6th UK and Ireland implementation science research conference

**DOI:** 10.1186/s13012-024-01341-3

**Published:** 2024-03-20

*Sustaining Health and Public Services in an Uncertain Future: What Role for Implementation Science?*

**Institute of Psychiatry, Psychology and Neuroscience, King's College London & University of Limerick**

## P1: Interventions to promote effective interprofessional collaboration that improves care outcomes for older people: a realist synthesis of evidence

### Carmel Davies^1^, Deirdre O’Donnell^1^, Apolonia Radomska^1^, Éidín Ní Shé^3^, Marie O’Shea^1^, Catherine Devaney^2^, Sarah Donnelly^1^, Gráinne O’Donoghue^1^, Aoife De Brún^1^, Helen Whitty^2^, PJ Harnett^2^, Deirdre Lang^2^, Reema Harrison^4^, Eilish McAuliffe^1^, Emer Ahern^2^

#### ^1^University College Dublin, Dublin 4, Ireland; ^2^National Clinical Programme for Older Persons, Dublin, Ireland; ^3^Royal College of Surgeons of Ireland, Dublin, Ireland; ^4^Macquarie University, Sydney, Australia

##### **Correspondence:** Carmel Davies (carmel.davies@ucd.ie)

*Implementation Science 2024*, **19(1)**:P1

**Background**

Within global healthcare policy, interprofessional integrated models of care are integral to quality healthcare for older people yet are challenging to implement [1,2,3]. Evidence to guide implementation remains underdeveloped [4]. Drawing on international research evidence, this review identifies interventions to promote effective interprofessional collaboration for improving care for older people. Healthcare implementation is complex and influenced by context, explaining why an intervention might work in one place and not another. Realist approaches explain ‘how’ complex interventions work through dynamic interactions between context, mechanisms, and outcomes [5].

**Methods**

The review follows the reporting standards of realist reviews [6]. A systematic search in PubMed, CINAHL, PsycINFO, EMBASE and SCOPUS identified primary research studies from 2013-2023. Data were extracted from the theory of interprofessional collaboration in community for older people. Context, interventions, outcomes, and mechanisms were identified and developed into Programme Theories (PTs).

**Results**

A suite of PTs in the form of Context Mechanism and Outcomes (CMOs) and Resource (R) configurations are presented with the following as an example:

Where CST members have professional experience in different areas of service delivery (C), they bring to the team pre-existing relationships with other HCPs (R), enabling good cross sectoral communication and trust (M), leading to enhanced continuity of care (O).

**Conclusion**

The paper explains what interventions work to support interprofessional collaboration and integrated care for older people. It provides a roadmap for implementation planning to help workforce planning and capacity building to improve older people’s health services. The realist synthesis is the first phase of a larger realist evaluation. The PTs will be tested and refined within Community Specialist Teams for Older People, implemented recently in Ireland as part of the National Integrated Care Programme for Older Persons [7]. PT empirically tested in local contexts enhance theory to support scale-up efforts across the Irish health system.

**Trial Registration:** Not applicable

**Consent to publish:** Not applicable

**References**

1. Science Advice for Policy by European Academics. Transforming the future of ageing. In Science Advice for Policy by European Academics. 2019. Available from https://sapea.info/topic/ageing/
2. World Health Organisation. Health workforce for ageing populations. (2016). Available from https://www.semeg.es/uploads/archivos/health-workforce-ageing-populations.pdf
3. Ní Shé, É. N., O’Donnell, D. E., O’Shea, M., & Stokes, D. New Ways of Working? A Rapid Exploration of Emerging Evidence Regarding the Care of Older People during COVID19. International Journal of Environmental Research and Public Health. 2020; 17(18): 6442. 10.3390/ijerph17186442
4. Ellis, G., & Sevdalis, N. Understanding and improving multidisciplinary team working in geriatric medicine. Age And Ageing*.* 2019: 48(4): 498–505. 10.1093/ageing/afz021
5. Rycroft-Malone, J., McCormack, B., Hutchinson, A.M. *et al.* Realist synthesis: illustrating the method for implementation research. Implementation Science. 2012; (7): 33. 10.1186/1748-5908-7-33
6. Wong, G., Westhorp, G., Manzano, A., Greenhalgh, J., Jagosh, J., & Greenhalgh, T. RAMESES II reporting standards for realist evaluations. BMC Medicine. 2016;14(1). 10.1186/s12916-016-0643-1
7. Department of Health. Sláintecare Implementation Strategy and Action Plan 2021-2023. Government of Ireland. 2021. Available from https://www.gov.ie/en/publication/6996b-slaintecare-implementation-strategy-andaction-plan-2021-2023/

## P2: The impact of after action review on safety culture and staff-wellbeing: findings from a mixed-methods effectiveness-implementation study in a hospital setting in Ireland

### Siobhan Eithne McCarthy^1^, Mairead Finn^1^, Gintare Valentelyte^2^, Fiona Boland^3^, Catherine Hogan^4^, Loretta Jenkins^4^, Theresa Keane^1^, Lisa Mellon^5^, Lorraine Schwanberg^4^, Aisling Walsh^6^, David Williams^7^, Natasha Rafter^8^

#### ^1^Graduate School of Healthcare Management, RCSI University of Medicine & Health Sciences, Dublin, Ireland; ^2^Healthcare Outcomes Research Centre, School of Population Health, RCSI University of Medicine & Health Sciences, Dublin, Ireland; ^3^Data Science Centre, School of Population Health, RCSI University of Medicine & Health Sciences; ^4^National Quality and Patient Safety Directorate, Health Service Executive, Ireland; ^5^Dept of Health Psychology, School of Population Health, RCSI University of Medicine & Health Sciences, Dublin, Ireland; ^6^Dept of Public Health & Epidemiology, School of Population Health, RCSI University of Medicine & Health Sciences, Dublin, Ireland; ^7^School of Medicine, RCSI University of Medicine & Health Sciences, Dublin, Ireland; ^8^School of Population Health, RCSI University of Medicine & Health Sciences, Dublin, Ireland

##### **Correspondence:** Siobhan Eithne McCarthy (smccarthy@rcsi.ie)

*Implementation Science 2024*, **19(1)**:P2

**Background**

Originating in the US Army and now in the Incident Management Framework (IMF) of the Irish Health Service, After Action Review (AAR) is a non-hierarchical facilitated approach to team learning. AAR enables groups to come to a shared mental model about what happened, why it happened and to identify learning. AAR has been linked to improved safety culture in the US fire-fighting sector. How best to support adoption of AAR in healthcare is unknown, as well its effectiveness[1]. Therefore, we examined the effect of AAR on safety culture and second victim experience (the psychological/physical impact of patient safety events on staff) and its implementation at an Irish hospital.

**Method**

Drawing on Proctor’s framework[2], we conducted a mixed-methods effectiveness-implementation study. Hospital staff completed surveys (Hospital Survey on Patient Safety Culture 2.0 and Second Victim Experience and Support Tool) before (May/June 2021), and at the end of a twelve-month AAR intervention period (Sept/Oct 2022). Core implementation strategies were the site adoption of AAR as part of the Health Service Executive IMF and the training of hospital selected staff (one in twelve) as AAR Facilitators using a simulation-based training programme. Six months after the training, using the Theoretical Domains Framework (TDF), focus groups were conducted with AAR Facilitators to explore the enablers and barriers to AAR implementation. Information about number of AAR meetings, their quality and financial costs were also estimated.

**Results**

Findings were triangulated using Proctor’s framework[2]. These demonstrate the domains of safety culture and second victim experience which improved/deteriorated at the end of the 12-month period, and the impact of the inner and outer context on AAR uptake. Recommendations for behaviour change techniques to support future AAR implementation are made.

**Conclusion**

Results will directly inform local hospital decision making and national policy approaches to incorporating AAR in hospitals in Ireland.

**Trial Registration:** Not applicable

**Consent to publish:** Not applicable

**References**

1. McCarthy SE, Keane T, Walsh A, Mellon L, Williams DJ, Jenkins L, Hogan C, Stuart C, Rafter N. Effect of after action review on safety culture and second victim experience and its implementation in an Irish hospital: A mixed methods study protocol. PLoS One. 2021 Nov 18;16(11):e0259887
2. Proctor E, Silmere H, Raghavan R, Hovmand P, Aarons G, Bunger A, Griffey R, Hensley M. Outcomes for implementation research: conceptual distinctions, measurement challenges, and research agenda. Administration and policy in mental health and mental health services research. 2011 Mar;38:65-76.

## O3: Process evaluation of the SMARThealth pregnancy hybrid type 2 cluster randomised controlled trial: a protocol

### Nicole Votruba^1,2^, Ankita Sharma^1^, Sreya Majumdar^3^, Sudhir Thout^3^, Deversetty Praveen^3,4^, Varun Arora^5^, Pallab K. Maulik^3,4^, Jane Hirst^1,2^

#### ^1^Nuffield Department of Women’s & Reproductive Health, University of Oxford, United Kingdom; ^2^The George Institute for Global Health UK, Imperial College London, London, United Kingdom; ^3^The George Institute for Global Health, India; ^4^The George Institute for Global Health, University of New South Wales, Sydney, NSW, Australia; ^5^Post Graduate Institute of Medical Science, Rohtak, India

##### **Correspondence:** Nicole Votruba (nicole.votruba@wrh.ox.ac.uk)

*Implementation Science 2024*, **19(1)**:O3

**Background**

Women who experience anaemia, gestational diabetes and hypertension during the perinatal phase are at high risk of long-term complications. However, effective low-cost strategies to integrate non-communicable disease screening into pregnancy care in low-income settings are rare [1]. SMARThealth Pregnancy (SHP2) is a hybrid type-2 effectiveness-implementation trial aiming to improve health during pregnancy and the first year after birth using a community-based, digital approach [2]. A detailed process evaluation will be carried out to determine the implementation outcomes and strategies of the intervention, to understand the effects (or lack thereof), to clarify assumptions around causal mechanisms, and to enhance understanding on generalisability. Aims: To (1) examine implementation outcomes, (2) identify contextual factors and mechanisms of action/impact, (3) understand mechanisms and strategies.

**Method**

A mixed-methods, theory-driven process evaluation will be performed, in parallel to the main SHP2 trial for both intervention and active control arms. The mixed-methods study design will assess the process evaluation objectives. Data collection will include quantitative data collection from the digital application, health/training records, surveys, qualitative interviews and focus groups with key stakeholders, ethnographic observation, documentary analysis, and notes audit. Implementation outcomes will be assessed using the RE-AIM framework (reach, effectiveness, adoption, implementation, maintenance) [3] and Proctor et al’s implementation outcomes typology [4]. The effectiveness of implementation strategies will be assessed using the Expert Recommendations for Implementing Change (ERIC) compendium [5]. Data analysis will apply mixed deductive and inductive thematic analysis.

**Results**

The analysis will aim to seek explanations for outcomes achieved in the SHP2 trial. Results will be analysed across and between clusters, allowing to compare/contrast context and implementation between them and with other clusters showing similar outcomes.

**Conclusion**

This process evaluation is part of the SHP2 effectiveness-implementation study. It will inform the iterative development of a future intervention scale-up and adoption, or in case of a null trial, to understand which factors contributed.

**Trial registration:** Not applicable

**Consent to publish:** Yes

**References**

1. Nagraj S, Kennedy SH, Jha V, Norton R, Hinton L, Billot L, et al. SMARThealth Pregnancy: Feasibility and Acceptability of a Complex Intervention for High-Risk Pregnant Women in Rural India: Protocol for a Pilot Cluster Randomised Controlled Trial. Frontiers in Global Women’s Health. 2021;2.
2. Hirst JE, Votruba N, Billot L, Arora V, Rajan E, Thout SR, et al. A community-based intervention to improve screening, referral, and follow-up of non-communicable diseases and anaemia amongst pregnant and postpartum women in rural India: study protocol for a cluster randomised trial Trials. 2023(In Review).
3. Glasgow RE, Harden SM, Gaglio B, Rabin B, Smith ML, Porter GC, et al. RE-AIM Planning and Evaluation Framework: Adapting to New Science and Practice With a 20-Year Review. Front Public Health. 2019;7:64.
4. Proctor E, Silmere H, Raghavan R, Hovmand P, Aarons G, Bunger A, et al. Outcomes for implementation research: conceptual distinctions, measurement challenges, and research agenda. Adm Policy Ment Health. 2011;38(2):65-76.
5. Powell BJ, Waltz TJ, Chinman MJ, Damschroder LJ, Smith JL, Matthieu MM, et al. A refined compilation of implementation strategies: results from the Expert Recommendations for Implementing Change (ERIC) project. Implementation Science. 2015;10(1):21.

## P5: The feasibility of implementing rapid-learning methodology to inform radiotherapy treatment: healthcare professionals’ views

### Arbaz Kapadi^1^, David P French^1^, Gareth Price^2,3^, Corinne Faivre-Finn^2,3^, Rebecca Holley^2,3^, Kate Wicks^2,3^

#### ^1^Manchester Centre of Health Psychology, The University of Manchester, Manchester, UK; ^2^The University of Manchester, Division of Cancer Sciences, Radiotherapy Related Research, Manchester, UK; ^3^The Christie NHS Foundation Trust, Wilmslow Road, Withington, Manchester, UK

##### **Correspondence:** Arbaz Kapadi (arbaz.kapadi@manchester.ac.uk)

*Implementation Science 2024*, **19(1)**:P5

**Background**

Pragmatic continuous learning approaches (‘rapid-learning’) using real-world data (RWD) have the potential to provide evidence to optimise interventions in radiotherapy [1,2]. RWD is the data routinely collected as standard of care about all patients. An NIHR-funded method-development study, RAPID-RT, is currently evaluating the clinical effectiveness of a rapid-learning approach within lung cancer and the feasibility of implementing rapid-learning in practice [2]. We report on radiotherapy professionals’ perceptions of rapid-learning and RWD, and identifying key factors that affect implementation in the clinic.

**Methods**

Interviews were conducted with radiotherapy professionals (*n*=23) based across five geographically diverse UK cancer sites. Interview participants included clinical oncologists, physicists, radiographers, treatment planning and digital services staff. Data collection took place between January and May 2023, analysing data using inductive thematic analysis [3].

**Results**

Participants’ opinions centred on four main themes (Table 1): 1) The alignment of rapid-learning methodologies with the reality of practice, 2) Variability of clinical and RWD, 3) The maturity of data and digital infrastructures for rapid-learning, 4) Support and evidence needed to convince adoption of rapid-learning approaches.

**Conclusion**

Rapid-learning approaches using RWD offer alternatives to traditional randomised controlled trials for the evaluation of changes in radiotherapy practice. They may also provide better external validity. However, rapid-learning is dependent upon the quality of supporting data. The development of data and digital infrastructures are necessary to improve data accessibility and quality, along with support mechanisms for implementation (e.g. analytical support, time, resource investment). This will strengthen the evidence needed to support rapid-learning approaches.

**Trial Registration:** Not applicable

**Consent to publish:** Not applicable

**References**

1. Price G, Mackay R, Aznar M, McWilliam A, Johnson-Hart C, Van Herk M, Faivre-Finn, C. Learning healthcare systems and rapid learning in radiation oncology: Where are we and where are we going?. Radiother Oncol. 2021, Nov;164:183-195
2. Price G, Devaney S, French DP, Holley R, Holm S, Kontopantelis E, et al. Can Real-world Data and Rapid Learning Drive Improvements in Lung Cancer Survival? The RAPID-RT Study. Clinical Oncol. 2022, Jan;34(6):407-410.
3. Braun G, Clarke, V. Thematic Analysis: A Practical Guide. London; SAGE: 2022

**Table 1 (Abstract P5):** Theme summary of study results

Theme	Theme Summary
**1] The alignment of rapid-learning methodologies with the reality of practice**	A rapid-learning approach using RWD can provide timely evidence and help to formalise changes (through an iterative learning process) that are routinely introduced but with little evaluation. The use of RWD further complements the vision to ‘learn from every patient’ offering greater potential for treatment personalisation.
**2] Variability of clinical and RWD**	Concerns around the quality of clinical and RWD extend to data collection (e.g. poor follow-up data), issues over data recording and sharing formats, incompleteness of data reporting (e.g. toxicity and PROM data), and data analysis (time, resource, skill).
**3]The maturity of data and digital infrastructures for rapid-learning**	The need to develop integrative data and digital infrastructures to enable timely access to data and data standardisation.
**4] Support and evidence needed to convince adoption of rapid-learning approaches.**	Support for implementation of rapid-learning includes time and resources for data collection, access to analytical support, and clear surveillance strategy (to increase confidence in the reliability of data). Further method clarification to better understand additional practice demands and provide guidance over critical data points (to minimise risk) when introducing treatment changes.

## O6: A protocol for developing a checklist tool that places intersectional inequalities at the centre of patient and public involvement activities

### Patrick Kierkegaard^1,2^

#### ^1^CRUK Convergence Science Centre, Imperial College London, London, SW7 2AZ, UK; ^2^NIHR London In Vitro Diagnostics Cooperative, Imperial College London, W2 1NY, UK

##### **Correspondence:** Patrick Kierkegaard (p.kierkegaard@imperial.ac.uk)

*Implementation Science 2024*, **19(1)**:O6

**Background**

To adequately address critical issues pertaining to intersectional inequalities, researchers must include patients as equal members of their research teams [1-3]. This process is known as patient and public involvement (PPIE). As important and valuable as PPIE is, there is no standard methodology for guiding and measuring collaboration between researchers and patients with regards to addressing intersectional health inequalities [4].

A few tools to support PPIE exist but they are designed for reporting purposes and are limited in scope [5-7]. They are not designed to assist researchers and patients in addressing intersectional inequalities.

This protocol describes the steps being taken to close this gap by designing a checklist tool that will allow researchers and patients determine to what extent their PPIE activities addresses critical issues relating to intersectional health inequalities.

**Method**

First, in-depth interviews will be conducted to understand contextual challenges associated with addressing and reporting intersectional inequalities during PPIE activities. Second, participatory design focus group will be conducted to co-design the new tool. Third, real-world projects will be utilized as case studies to pilot test and refine the new tool. This process will be guided by the Health Equity Implementation Framework [8].

**Results**

The primary objective of this study are the co-development of a checklist tool that will improve the methodologies used to ensure PPIE activities address intersectional health inequalities. A secondary objective of the tool is to pilot test it in order to evaluate potential improvements and its adoption into regular PPIE practice.

**Conclusion**

An important objective of this protocol is to establish a framework for the creation of a checklist tool that will complement existing PPIE report tools in order to ensure that patients and researchers engage in meaningful PPIE activities throughout the research cycle in order to address intersectional health inequalities.

**Trial Registration:** Not applicable

**Consent to publish:** Not applicable

**References**

1. Seale B: Patients as partners. Building collaborative relationships among professionals, patients, carers and communities London: The Kings Fund 2016.
2. Brett J, Staniszewska S, Mockford C, Herron‐Marx S, Hughes J, Tysall C, Suleman R: Mapping the impact of patient and public involvement on health and social care research: a systematic review. Health Expectations 2014, 17(5):637-650.
3. Baxter S, Muir D, Brereton L, Allmark C, Barber R, Harris L, Hodges B, Khan S, Baird W: Evaluating public involvement in research design and grant development: using a qualitative document analysis method to analyse an award scheme for researchers. Research Involvement and Engagement 2016, 2(1):1-15.
4. Staley K: ‘Is it worth doing?’ Measuring the impact of patient and public involvement in research. Research Involvement and Engagement 2015, 1(1):6.
5. Price A, Schroter S, Snow R, Hicks M, Harmston R, Staniszewska S, Parker S, Richards T: Frequency of reporting on patient and public involvement (PPI) in research studies published in a general medical journal: a descriptive study. BMJ open 2018, 8(3):e020452.
6. Weschke S, Franzen DL, Sierawska AK, Bonde L-S, Strech D, Schorr SG: Reporting of patient involvement: a mixed-methods analysis of current practice in health research publications using a targeted search strategy. BMJ Open 2023, 13(1):e064170.
7. Turnhout E, Metze T, Wyborn C, Klenk N, Louder E: The politics of co-production: participation, power, and transformation. Current Opinion in Environmental Sustainability 2020, 42:15-21.
8. Woodward EN, Singh RS, Ndebele-Ngwenya P, Melgar Castillo A, Dickson KS, Kirchner JE: A more practical guide to incorporating health equity domains in implementation determinant frameworks. Implementation Science Communications 2021, 2(1):61.

## P7: Transdisciplinary implementation science for complex futures

### Hossai Gul^1,3^, Stephanie Best^2,4^, Janet Long^3,4^, Ellenore Martin^4^, Lucinda Murray^5^, Vanessa Fitzgerald^6^, Frances Rapport^3^, Mike Field^4,5^, Jeffrey Braithwaite^3,4^

#### ^1^TD School, University of Technology Sydney, Sydney, NSW, 2007, Australia; ^2^Peter MacCallum Cancer Centre, University of Melbourne, Melbourne, VIC, 3010, Australia; ^3^Australian Institute of Health Innovation, Macquarie University, Sydney, NSW, 2109, Australia; ^4^Australian Genomics, Melbourne, VIC, 3051, Australia; ^5^Hunter New England Local Health District, New Lambton, NSW, 2305, Australia; ^6^NSW Ministry of Health, St Leonards, NSW, 2065, Australia

##### **Correspondence:** Hossai Gul (hossai.gul@uts.edu.au)

*Implementation Science 2024*, **19(1)**:P7

**Background**

The innovation-to-implementation gap remains an elusive wicked challenge across sectors from health, education, social services, and business. Implementation science offers effective tools for guiding change; however, reductionist application of these tools risks the discipline becoming redundant amidst rapidly evolving complex systems. This presentation reports on a transdisciplinary approach to conducting implementation science research.

**Methods**

A sequence of studies were conducted via a mixed method methodology and structured by the process model Implementation Mapping (IM) to guide the development of implementation strategies. Ten tools were used to operationalise each step of IM via a transdisciplinary co-production process.

**Results**

Transdisciplinary research is distinct from multidisciplinary (the coordinated effort to solve a problem but remaining within disciplinary boundaries) and interdisciplinary (coherent synthesis of knowledge from a variety of disciplines). Implementation science is an interdisciplinary field where knowledge from many disciplines have been integrated into theories, models, and frameworks which focus on various aspects of implementation. However, solving complex implementation challenges cannot be approached by implementation science alone. Implementation is a hyper-connected challenge and requires the input of many disciplines and spheres of individuals and groups outside of research. Transdisciplinary implementation science incorporates the following features: (1) integration of knowledge from many disciplines in a way that transcends and responds to implementation challenges, (2) the research involves participation by actors outside of the research sphere (going beyond consumers) and values their expertise and experience as indispensable for effective implementation, (3) implementation complexity is centralised via a holistic systems lens, (4) the implementation research is action-oriented (practical strategy deployment), future-focused (scale and sustainability), and impact-driven (outputs are secondary).

**Conclusion**

Transdisciplinary implementation science was a challenge in practice in numerous forms, however, the process was continuously improved via reflexivity by the research team which led to more impactful implementation research and practice.

**Trial Registration:** Not applicable

**Consent to publish:** Not applicable

## P8: Systems science approach to conducting and integrating implementation assessments across multiple settings within complex healthcare systems

### Hossai Gul^1,3^, Stephanie Best^2,4^, Janet Long^3,4^, Ellenore Martin^4^, Lucinda Murray^5^, Vanessa Fitzgerald^6^, Frances Rapport^3^, Mike Field^4,5^, Jeffrey Braithwaite^3,4^

#### ^1^TD School, University of Technology Sydney, Sydney, NSW, 2007, Australia; ^2^Peter MacCallum Cancer Centre, University of Melbourne, Melbourne, VIC, 3010, Australia; ^3^Australian Institute of Health Innovation, Macquarie University, Sydney, NSW, 2109, Australia; ^4^Australian Genomics, Melbourne, VIC, 3051, Australia; ^5^Hunter New England Local Health District, New Lambton, NSW, 2305, Australia; ^6^NSW Ministry of Health, St Leonards, NSW, 2065, Australia

##### **Correspondence:** Hossai Gul (hossai.gul@uts.edu.au)

*Implementation Science 2024*, **19(1)**:P8

**Background**

Implementation needs assessments are a critical first stage in implementation research and can consist of mapping target behaviours, processes, and barriers and facilitators. These barriers and facilitators are often reported as discrete lists despite the complex, interrelated, and dynamic reality that shapes implementation determinants. The aim of this body of work was to conduct an implementation needs assessment that provided a holistic view of implementation determinants to guide the development of evidence-informed implementation strategies in support of real-world genomic implementation efforts.

**Methods**

The model of care being implemented combined specialist clinical genetics services with nongenetic primary paediatric services. The implementation needs assessment began within genetics services via qualitative semi-structured interviews (*n*=14 participants, clinical genetics professionals) and continued within paediatrics via a cross-sectional survey (*n*=114 respondents, paediatricians) and semi-structured interviews (*n*=22, paediatricians). The resultant data were analysed using: (1) the Interactive Systems Framework (ISF) for mapping the implementation system; (2) pathway mapping techniques to visualise changes required in processes and practices; (3) Implementation Mapping to identify target behaviours; (4) mapping of implementation barriers and facilitators using the Consolidated Framework for Implementation Research (CFIR) (within genetic services) and the TDF in combination with descriptive statistics (within paediatric services); and (5) to integrate all findings a rich picture was developed using soft system methodology.

**Results**

A systems science approach to implementation needs assessment revealed the specific relationships between barriers and facilitators that informed strategy design, bundling, and sequence of deployment. A rich picture view of implementation needs within a system also provided clear leverage points and areas within the system that change was not feasible, ethical, or outside of the sphere of influence of implementation teams – enabling better resource utilisation.

**Conclusion**

Integrating implementation science tools with systems science tools allows for a more effective implementation needs assessments rooted in truer depictions of complex realities.

**Trial Registration:** Not applicable

**Consent to publish:** Not applicable

## P9: The post-implementation scenario: investigating the sustainability of matrix support through professional practices

### Ana Laura Salomé Lourencetti^1^, Carlos Alberto dos Santos Treichel^2^, Maria Giovana Borges Saidel^1^, Rosana Teresa Onocko Campos^3^

#### ¹School of Nursing, State University of Campinas, Campinas-SP, Brazil; ²Department of Maternal Child and Psychiatric Nursing, School of Nursing, University of São Paulo, Brazil; ³Department of Collective Health, School of Medical Sciences, State University of Campinas, Campinas-SP, Brazil

##### **Correspondence:** Ana Laura Salomé Lourencetti (laurana.salome@gmail.com)

*Implementation Science 2024*, **19(1)**:P9

**Background**

In Brazil, matrix support is a collaborative work program that proposes shared and integrated care in mental health[1]. Similar to other health policies, its sustainability depends on several factors, such as the behavioral change of health workers in practice. This study aims to investigate health workers’ perceptions of the matrix support implementation process in a medium-sized municipality seeking the barriers and facilitators for its sustainability in their practices according to the domains proposed by the Theoretical Domains Framework (TDF)[2].

**Methods**

The implementation process took place between 2019 and 2021. One year after completion, primary and specialized mental health care professionals participate in two qualitative methodologies: in-depth interviews and observation during routine activities through previous scripts built based on the Theoretical Domains Framework to identify the sustainability of matrix support. The models proposed by Cane et al. (2012) [2] and Huijg et al. (2014) [3] were used to guide the scripts and analysis of the results.

**Results**

Eleven professionals participated in the interviews, and approximately 50 professionals were observed during team and matrix support meetings. After a hybrid analysis of the contents collected, it was identified that the most significant facilitators related to domains of knowledge, skills, and beliefs about capabilities. The barriers were related with contexts and resources (material resources and organizational culture) and beliefs about consequences.

**Conclusion**

The domains of knowledge, skills and beliefs about capabilities can be associated with the effectiveness of the training implementation strategies used during the implementation process. However, the reported barriers overlap: the sustainability of matrix support was affected by the professionals’ lack of belief in the effectiveness of the program in a context with a lack of resources and organizational barriers. This study also reinforces the use of TDF to identify barriers and facilitators that support the practice of health policies in the services routine.

**Trial Registration:** Not applicable

**Consent to publish:** Not applicable

**References**

1. Campos GWS, Domitti AC. Apoio matricial e equipe de referência: uma metodologia para gestão do trabalho interdisciplinar em saúde. Cad Saude Publica. 2007; 23(2):399-407.
2. Cane, J., O’Connor, D. & Michie, S. Validation of the theoretical domains framework for use in behaviour change and implementation research. Implementation Sci 7, 37 (2012). 10.1186/1748-5908-7-37
3. Huijg, J.M., Gebhardt, W.A., Crone, M.R. et al. Discriminant content validity of a theoretical domains framework questionnaire for use in implementation research. Implementation Sci 9, 11 (2014). 10.1186/1748-5908-9-11

## P10: Qualitative exploration of the views and experiences of making every contact count and within service providers and users within the third and social economy sector: a reflexive thematic analysis of semi-structured interviews

### Beth Nichol^1^, Rob Wilson^2^, Angela Rodrigues^3^, Catherine Haighton^1^

#### ^1^Department of Social Work, Education, and Community Wellbeing, Northumbria University, Newcastle upon Tyne, UK; ^2^Newcastle Business School, Northumbria University, Newcastle upon Tyne, UK; ^3^Department of Psychology, Northumbria University, Newcastle upon Tyne, UK

##### **Correspondence:** Beth Nichol (bethany.nichol@northumbria.ac.uk)

*Implementation Science 2024*, **19(1)**:P10

**Background**

The Making Every Contact Count (MECC) initiative encourages brief, opportunistic advice around health and wellbeing. Minimal research exists on MECC within the Third and Social Economy (TSE) sector (groups or organisations operating independently to family and government with social justice as the primary aim), despite increasing funding and training roll-out in this area. The current study aimed to assess the barriers and facilitators of implementation of MECC within the TSE and consider how training and delivery of MECC could be amended to optimise implementation within this setting.

**Methods**

Purposive sampling was applied to capture a wide variety of TSE settings including charities, religious settings, and youth clubs. To explore whether MECC conversations are already occur without formal MECC training, service provider participants did not need to have received MECC training. 20 qualitative semi-structured interviews were conducted with service users (*n* = 5) and providers (*n* = 15). Reflexive thematic analysis was applied using Nvivo.

**Results**

Health and wellbeing conversations occur naturally within these settings, without the need for specific training. However, unlike traditional MECC conversations, these conversations emphasise passivity, namely waiting for the service user to initiate and listening without provision of advice. Trusting relationships facilitate conversations between service users and providers within TSE settings, but also act as a barrier to initiating MECC conversations due to fear of damaging these relationships. Service providers draw upon a breadth of previous experience to apply advanced interpersonal skills. However, having the resources to signpost to further services, ideally internally, is essential.

**Conclusion**

MECC training should be adapted for TSE settings, with an acknowledgement that conversations around health and wellbeing already occur. Service providers within the TSE particularly would benefit from training on how to initiate conversations around health and wellbeing and play an active role in assisting the person to realise health behaviour change.

**Trial Registration:** Not applicable

**Consent to publish:** Not applicable

## P11: Implementing change relationships in multi-site youth justice settings- a methodological design

### Jacqueline Dwane^1^, Caitlin Lewis^1^, Eoin O’Meara Daly^1^ Sean Redmond^1^ Alice Coffey^2^

#### ^1^Research Evidence into Programmes, Policy and Practice (REPPP), School of Law, Faculty of Arts, Humanities and Social Science, University of Limerick, Ireland; ^2^Department of Nursing and Midwifery, Faculty of Education and Health Sciences, University of Limerick, Ireland

##### **Correspondence:** Jacqueline Dwane (Jacqueline.dwane@ul.ie)

*Implementation Science 2024*, **19(1)**:P11

**Background**

This paper outlines a methodological approach to implementation research in youth justice settings. A multi-site study examined relationships between youth justice practitioners and young people in Youth Diversion Projects (YDPs). We found that effective relationships in YDPs are a crucial change mechanism in reducing offending. We coded findings from a practitioner-led evaluation to the updated CFIR [1] to categorise implementation determinants. We co-designed an evidence-informed relationship model to guide effective relationship practices. The policymaker endorsed full scale out of the model. Young people can gain from high-quality practitioner relationships in all YDPs (n105).

**Methods**

The findings from the multi-site (n16) study inform four discrete implementation strategies [2]. These involve capacity development and support measures to implement agile and effective relationships. We will conduct an antecedent assessment of attitudes towards the practice change [3]. A process evaluation will examine practitioners’ experience of the model and capacity development strategies. Quarterly online meetings will collect systematic real-time qualitative insights from 105 project teams. We will also ask projects to answer one prescribed question to measure success as part of their discussion. We will record the transcripts from this longitudinal study for analysis.

**Results**

We will provide quarterly evidence of the implementation experience to the funder. Later, we will analyse the data according to the Implementation Outcomes Framework [4].

**Conclusion**

This paper builds on knowledge from a three-year action research process. It proposes a method to advance scale-out and implement an evidence-informed relationship model. We welcome guidance from other methodologists and implementation experts to advance the methodology.

**Trial Registration:** Not applicable

**Consent to publish:** Not applicable

**References**

1. Damschroder, L.J., Reardon, C.M., Widerquist, M.A.O. *et al.* (2022). The updated Consolidated Framework for Implementation Research based on user feedback. Vol. 17. *Implementation Science*, 75.
2. Powell, B.J., Waltz, T.J., Chinman, M.J., *et al.* (2015). A refined compilation of implementation strategies: results from the Expert Recommendations for Implementing Change (ERIC) project. Vol. 10. *Implementation Science.*
3. Weiner, B.J, Lewis, C.C., Stanick, C, Powell B.J. *et al.* (2017) Psychometric assessment of three newly developed implementation outcome measures. Vol. 12. *Implementation Science*, 1.
4. Proctor, E.K., Silmere, H., Raghavan, R. *et al.* (2011) Outcomes for implementation research: conceptual distinctions, measurement challenges, and research agenda. Vol. 38. *Adm Policy Mental Health*

## P12: Insights on barriers, facilitators, and lessons learned in the implementation of complex suicide prevention interventions: a systematic review

### Sadhvi Krishnamoorthy^1^, Sharna Mathieu^1^, Gregory Armstrong^2^, Victoria Ross^1^, Jillian Francis^3,4,5^, Lennart Reifels^6^, Kairi Kõlves^1^

#### ^1^Australian Institute for Suicide Research and Prevention, World Health Organization Collaborating Centre for Research and Training in Suicide Prevention, School of Applied Psychology, Griffith University, Queensland, Australia; ^2^Nossal Institute for Global Health, Melbourne School of Population and Global Health, University of Melbourne, Victoria, Australia; ^3^School of Health Sciences, The University of Melbourne, Victoria, Australia; ^4^Department of Health Services Research, Peter MacCallum Cancer Centre, Victoria, Australia; ^5^Clinical Epidemiology Program, Ottawa Hospital Research Institute, Ottawa, Ontario, Canada; ^6^Centre for Mental Health, Melbourne School of Population and Global Health, The University of Melbourne, Victoria, Australia

##### **Correspondence:** Sadhvi Krishnamoorthy (sadhvi.krishnamoorthy@griffithuni.edu.au)

*Implementation Science 2024*, **19(1)**:P12

**Background**

Understanding what works in preventing suicidal behaviour is complex and remains largely unaddressed. A clear evidence-practice gap exists. One of the ways to bridge this gap is to understand the influence of determinants on intervention delivery, adoption, and sustainment along with experiences and lessons learned on the ground. This study examines barriers, facilitators and lessons learned from implementing complex suicide prevention interventions across the world.

**Methods and materials**

This study reports on data from a comprehensive systematic review of complex suicide prevention interventions, using updated PRISMA guidelines. All English language records (including grey literature) between 1990-2022 were searched on PubMed, CINAHL, PsycINFO, ProQuest, SCOPUS and CENTRAL. Interventions were defined as being *complex* if they consisted of three or more components, implemented across two or more levels of the social ecology. Data on barriers, facilitators and lessons learned was extracted from clusters of reports on interventions and were mapped using the updated Consolidated Framework for Implementation Research (CFIR).

**Results**

The most common barriers were reported in the inner setting domain and were related to the compatibility of the intervention, culture and maintaining relational connections. The most reported facilitators were related to the individual motivation, capability, and need. Lessons learned focused on the importance of adaptation and ensuring responsiveness to contextual needs.

**Limitations**

Data on barriers, facilitators and lessons learned was inferred from the reports included in the study and hence was limited in its understanding of implementation experiences.

**Conclusion**

This study emphasises the importance of documenting and analysing important influences on the implementation process. This information can help develop a better understanding of how the evidence-practice translation happens in suicide research and prevention.

**Trial Registration:** Not applicable

**Consent to publish:** Not applicable

## P13: Understanding implementation and impact of post-Covid remote monitoring pathways in integrated care systems

### Judith Fynn^1^, Adam Wagner^3,6^, Lisa Miners^3,6^, Katherine Cummergen^1^, Sophie Knight^1^, Alan Bellinger^4^, Kevin Minier^5^, Sarah Rae^5^, Jennifer Lynch^2,6^

#### ^1^Health Innovation East, Unit C, Magog Court, Shelford Bottom, Cambridge, CB22 3AD, UK; ^2^University of Hertfordshire, Hatfield, Hertfordshire AL10 9AB, UK; ^3^University of East Anglia, Norwich Research Park, Norwich NR4 7TJ, UK; ^4^Healthwatch Hertfordshire, Kings Court, London Rd, Stevenage SG1 2NG, UK; ^5^East of England Citizen’s Senate, Unit C, Magog Court, Shelford Bottom, Cambridge, CB22 3AD, UK; ^6^NIHR Applied Research Collaboration East of England, Douglas House,18 Trumpington Road, CB2 8AH

##### **Correspondence:** Judith Fynn (judith.fynn@eahsn.org)

*Implementation Science 2024*, **19(1)**:P13

**Background**

Remote monitoring (RM) enables observation and reporting of physiology and behaviour with the intention of supporting patients to self-manage their conditions. In England, RM is central to the National Health Service (NHS) recovery from the COVID-19 pandemic and the government’s plan to drive efficiency, free up hospital beds, clinician time and reduce the COVID backlog [1]. Improving understanding of RM implementation and its impacts on patients, staff, carers and health and care systems is critical to enable system resilience post pandemic.

We evaluated the implementation of technology-enabled RM pathways at four sites within different Integrated Care Systems (ICS), each varying in health condition/patient cohort and delivery model.

**Method**

The four mixed method evaluations were designed and conducted with patient and public involvement (PPI). The evaluations had distinct overlapping phases: (1) pathway mapping and logic modelling; (2) quantitative analysis to understand patient characteristics, resources and costs and qualitative staff interviews to understand delivery and impacts; (3) knowledge mobilisation.

**Results**

We used the NASSS framework [2] to identify key factors affecting the implementation of RM pathways in ICS. Cross-cutting themes included: potential for access inequities; system-level challenges and enablers; importance of reporting, sharing and use of data. Variability in data recorded and informatics processes within health systems affects the ability to fully understand patient characteristics, including excluded patients, and wider impacts of RM pathways on staff, patients and carers. Clinical champions were key to driving the development and delivery of RM pathways. Differing staffing and delivery models influenced acceptability and potential for scale-up, spread and sustainability.

**Conclusion**

Understanding impacts of different implementation models, including staffing integrated working and co-production, is critical to enabling health systems to deliver RM at scale. Improved data sharing and recognition of system level resource requirements is critical to sustaining delivery of novel pathways in the new ICS infrastructure and improving patient experience.

**Trial Registration:** Not applicable

**Consent to publish:** Not applicable

**Acknowledgement**

This work was produced as part of an NHS Insights Prioritisation Project (NIPP) jointly commissioned by the Accelerated Access Collaborative (AAC) at NHS England and the National Institute of Health Research (NIHR) and was undertaken as part of a collaboration between the Applied Research Collaboration East of England and Health Innovation East.

**References**

1. Department of Health and Social Care. (2022, June 28). Digital revolution to bust COVID backlogs and deliver more tailored care for patients. *GOV.UK*. https://www.gov.uk/government/news/digital-revolution-to-bust-covid-backlogs-and-deliver-more-tailored-care-for-patients
2. NASSS reference: Greenhalgh T, Wherton J, Papoutsi C, Lynch J, Hughes G, A’Court C, Hinder S, Fahy N, Procter R, Shaw S. Beyond Adoption: A New Framework for Theorizing and Evaluating Nonadoption, Abandonment, and Challenges to the Scale-Up, Spread, and Sustainability of Health and Care Technologies. J Med Internet Res. 2017 Nov 1;19(11):e367. 10.2196/jmir.8775. PMID: 29092808; PMCID: PMC5688245.

## P14: The connected Yorkshire community health checks programme for cardiovascular disease prevention

### Ciaran O’Neill, Zuneera Khurshid, Vishal Sharma, Michael McCooe

#### Improvement Academy, Bradford Institute for Health Research, Temple Bank House, Duckworth Lane Bradford, BD9 6RJ, UK

##### **Correspondence:** Ciaran O’Neill (ciaran.o'neill@bthft.nhs.uk)

*Implementation Science 2024*, **19(1)**:P14

**Background**

Bradford District is one of the most deprived local authorities in England[1] and the rate of Cardiovascular Disease is also higher than the national average[2]. This project was co-designed in response to a community expectation to implement pre-emptive actions to reduce this risk. The aim of this project is to implement a series of outreach health check events with a community centred approach for cardiovascular disease prevention, and improving community access to social prescribers and primary care.

**Methods**

Community engagement and co-design are central to the implementation process. It is a mixed-methods evaluation based on Implementation Science and Participatory Action Research principles. The quantitative measures include hypertension case finding, identification of pre-diabetes and improved blood pressure and HbA1c control. The qualitative implementation evaluation uses a combination of traditional implementation science and rapid qualitative evaluation approaches using the Stanford Lightning Reports method[3] to explore contextual factors that impact implementation, and assess implementation fidelity, appropriateness, and feasibility.

**Results**

The first event was in February 2023, 61% of the 103 who attended had an abnormal blood pressure and two subsequently received a new diagnosis of Type 2 Diabetes. The event was perceived as useful by the community. Initial analysis revealed good community and stakeholder engagement, and access to funding were major implementation facilitators. Scope creep, unclear commitment from some stakeholders, system and structural challenges, and limiting beliefs of some community members about health improvement emerged as significant implementation barriers.

**Conclusion**

The health check event was successful in responding to community health needs, and developing and implementing a community-based, co-designed approach. Initial data demonstrated usefulness of the approach and its potential for improving engagement amongst people from deprived communities; however system level barriers remain a significant challenge to the sustainability of the intervention.

**Trial Registration:** Not applicable

**Consent to publish:** Not applicable

**References**

1. City of Bradford Metropolitan District Council. Joint Strategic Needs Assessment, The Population of Bradford District. Bradford. 2022. Available from: https://jsna.bradford.gov.uk/documents/The%20population%20of%20Bradford%20District/1.2%20Health%20Inequalities%20and%20Life%20Expectancy/Health%20Inequalities%20and%20life%20expectancy.pdf date accessed 18^th^ July 2022.
2. Bradford’s Healthy Hearts: better management of CVD patients [Internet]. GOV.UK. Available from: https://www.gov.uk/government/case-studies/bradfords-healthy-hearts-better-management-of-cvd-patients
3. Brown‐Johnson C, Safaeinili N, Zionts D, Holdsworth LM, Shaw JG, Asch SM, Mahoney M, Winget M. The Stanford Lightning Report Method: a comparison of rapid qualitative synthesis results across four implementation evaluations. Learning Health Systems. 2020 Apr;4(2):e10210.

## P15: Tailoring implementation strategies which target patients in healthcare contexts: protocol for a scoping review sub-study

### Laura-Jane McCarthy^1^, Fiona Riordan^1^, Jane Murphy^1^, Nickola Pallin^1^, Claire Kerins^2^, Bianca Albers^3^, Lauren Clack^3^, Eimear Morrissey^4^, Geoffrey M. Curran^5^, Cara C. Lewis^6^, Byron J. Powell^7^, Justin Presseau^8^, Luke Wolfenden^9^, Sheena M. McHugh^1^

#### ^1^School of Public Health, University College Cork, Cork, Ireland; ^2^Health Promotion, University of Galway, Galway, Ireland; ^3^Institute for Implementation Science in Health Care, University of Zurich, Zurich, Switzerland; ^4^School of Psychology, University of Galway, Galway, Ireland; ^5^Department of Psychiatry, University of Arkansas for Medical Sciences, Little Rock, AR, USA; ^6^Kaiser Permanente Washington Health Research Institute, 1730 Minor Avenue, Suite 1600, Seattle, WA, 98101, USA; ^7^Brown School, Washington University in St. Louis, One Brookings Drive, Campus Box 1196, St. Louis, MO, 63130, USA; ^8^Clinical Epidemiology, Ottawa Hospital Research Institute, Ottawa, Canada; ^9^School of Medicine and Public Health, College of Health, Medicine, and Wellbeing, the University of Newcastle, Callaghan, NSW, Australia

##### **Correspondence:** Laura-Jane McCarthy (laura-janemccarthy@ucc.ie)

*Implementation Science 2024*, **19(1)**:P15

**Background**

Tailoring strategies to target salient barriers to and enablers of implementation is considered important to support the successful delivery and uptake of evidence-based healthcare interventions (EBI). We are conducting a scoping review to explore how tailoring has been conceptualised, operationalised, and evaluated in healthcare contexts. Our understanding of how to tailor patient-level strategies to support the uptake of an EBI (e.g., screening intervention) is limited; this extends to cultural tailoring, whereby consideration is given to the culture, language and local factors that reflect the preferences and needs of patients[1]. How and when patients should be included in the tailoring process is also unclear. To address this gap, we will conduct a sub-analysis which will specifically focus on tailoring strategies which target patients.

**Methods**

The scoping review is being conducted in line with best practice guidelines [2] and will be reported in accordance with the Preferred Reporting Items for Systematic Reviews and Meta-analysis extension for scoping reviews (PRISMA-ScR). Searches have been conducted of MEDLINE, Embase, Web of Science, Scopus, from 2005 to present. Articles related to cultural tailoring or tailoring of patient-level implementation strategies will be isolated and sub-analysed. Analysis will be quantitative, including descriptive numerical summaries of study characteristics and the tailoring process. Qualitative content analysis will also be conducted.

**Results**

Title/abstracts of 5936 articles identified through database searches have been screened. Full text screening of 956 articles is currently being conducted by the research team and expected to be completed by August 2023.

**Conclusion**

Findings from this sub-study will identify how patient level tailoring, including cultural tailoring of implementation strategies has been conceptualised, operationalised, and evaluated in healthcare. Furthermore, findings may provide more clarity on how and when patients should be included in the tailoring process.

**Trial Registration:** Not applicable

**Consent to publish:** Not applicable

**References**

1. Cabassa LJ, Baumann AA. A two-way street: Bridging implementation science and cultural adaptations of mental health treatments. Implementation Science. 2013;8(1). 10.1186/1748-5908-8-90.
2. Levac D, Colquhoun H, O’Brien KK. Scoping studies: Advancing the methodology. Implementation Science. 2010;5(1). 10.1186/1748-5908-5-69.

## O16: A policy lab to accelerate translation of novel research into policy to improve timely detection and appropriate action in care of women with pre-eclampsia in Sierra Leone

### Katy Kuhrt^1^, Osman Koroma^2^, Alexandra Ridout^1^, Francis Smart^3^, Harriet Boulding^4^, Andrew Shennan^1^ on behalf of the NIHR CRIBS Global Maternal Health Group

#### ^1^Department of Women and Children’s Health, King’s College London, London, UK; ^2^Welbodi Partnership, Freetown, Sierra Leone; ^3^Department of Policy, Planning and Information, Ministry of Health and Sanitation, Sierra Leone; ^4^The Policy Institute, King’s College London, London, UK

##### **Correspondence:** Katy Kuhrt (katykuhrt24@gmail.com)

*Implementation Science 2024*, **19(1)**:O16

**Background**

Pre-eclampsia is the second leading cause of maternal death globally, including Sierra Leone, where women are 2000 times more likely to die compared to the UK[1]. Key reasons are delayed detection and lack of appropriate action (anti-hypertensives, anticonvulsants, delivery). In Sierra Leone, we demonstrated that early identification of abnormal vital signs is associated with a reduction in maternal mortality[2], and enables targeted interventions (early delivery), which saves babies lives (NNT = 30), and reduces severe maternal hypertension. Policy Labs are an engagement approach used to facilitate research evidence uptake into policy and practice. Integration of this novel evidence into maternity care is critical to minimise adverse outcomes.

**Methods**

Based on the ‘trust-translation-timing’ model developed by King’s Policy Institute[3] we co-hosted a Policy Lab with the Ministry of Health and Sanitation in Sierra Leone attended by a diverse group of stakeholders, who received a briefing pack synthesizing key evidence prior to the event. Participants discussed barriers and facilitators in small, mixed groups and devised collaborative strategies for translation of the new research into pre-eclampsia management.

**Results**

39 attendees (women, community representatives, religious leaders, health workers, policy makers and politicians) identified multiple challenges, i.e.: community beliefs that eclamptic women are possessed by ‘demons’, cost of transport and treatment and lack of trust in healthcare. Key recommendations included intentional community engagement through public health education campaigns, and specialized Pre-eclampsia Care centres. 15 participants formed a technical working group and are currently involved in development and delivery of a national pre-eclampsia awareness programme.

**Conclusion**

Early detection and appropriate action is a critical issue for pre-eclampsia management in Sierra Leone. Policy Labs are an effective tool to facilitate the co-development of evidence-based collaborative policies, including community education and empowerment, to expedite reduction in mother and infant morbidity and mortality.

**Trial Registration:** Not applicable

**Consent to publish:** Not applicable

**References**

1. Shennan AH, Green M, Chappell LC. Maternal deaths in the UK: pre-eclampsia deaths are avoidable. Lancet. 2017;389(10069):582-4.
2. Vousden N, Lawley E, Nathan HL, Seed PT, Gidiri MF, Goudar S, et al. Effect of a novel vital sign device on maternal mortality and morbidity in low-resource settings: a pragmatic, stepped-wedge, cluster-randomised controlled trial. The Lancet Global Health. 2019;7(3):e347-e56.
3. Hinrichs-Krapels S, Bailey J, Boulding H, Duffy B, Hesketh R, Kinloch E, et al. Using Policy Labs as a process to bring evidence closer to public policymaking: a guide to one approach. Palgrave Communications. 2020;6(1):101.

## P17: A cross-sectional exploration of the generative mechanisms and potential staff outcomes associated with interprofessional collaboration within the newly established community specialist teams integrating care for older people in Ireland

### Apolonia Radomska^1^, Dr Deirdre O’Donnell^1^, Helen Whitty^2^, Catherine Devaney^2^, Dr Emer Ahern^2^, Dr PJ Harnett^2^

#### ^1^School of Nursing, Midwifery and Health Systems, University College Dublin, Dublin, Ireland; ^2^National Clinical Programme for Older People (NCPOP), Dublin, Ireland

##### **Correspondence:** Apolonia Radomska (apoloniaradomska1@ucd.ie)

*Implementation Science 2024*, **19(1)**:P17

**Background**

The National Clinical Programme for Older Persons Service Model [1] describes comprehensive service delivery supported by interdisciplinary teams transitioning care for older people along end-to-end integrated care pathways. Interprofessional Collaboration (IPC) is core to the implementation of this service model. However, there is an evidence gap in understanding what works to support IPC in this context. Furthermore, little is known about the staff outcomes that may be associated with these new ways of working for Medicine, Nursing and Health and Social Professionals (HSCPs) employed in interprofessional teams.

This study describes the generative mechanisms and staff outcomes that may be associated with IPC among professionals employed in the 30 newly established community specialist teams for older persons (CST-OPs). This local stakeholder knowledge supports an ongoing realist review of international evidence generating initial programme theory (IPTs) on what works and why to support IPC in community-based care integration for older people.

**Methods**

A cross-sectional representative survey of members (*N*=69) employed in the 30 CST-OPs was undertaken. The survey measured indicators of competence identified in a co-designed ECLECTIC framework for core competencies for IPC in interdisciplinary care teams for older persons [2]. These included generative mechanisms such as internal team processes and systems, as well as values and beliefs supporting collaboration.

**Results**

The findings describe the generative mechanisms associated with IPC and outcome factors for staff including higher job satisfaction, work engagement, trust and psychological safety. Findings elaborate on instances of service innovation which resulted from IPC.

**Conclusion**

The findings support the development of IPTs hypothesising the dynamic relationship between context, mechanisms and staff outcomes associated with team collaboration. These IPTs will be tested, refined and expanded through a planned realist implementation evaluation of the co-designed ECLECTIC framework among CST-OPs. This realist evidence will support implementation of the Older Person’s Service Model.

**Trial Registration:** Not applicable

**Consent to publish:** Not applicable

**References**

1. Enhanced Community Care [Internet]. HSE.ie. Available from: https://www.hse.ie/eng/services/list/2/primarycare/enhanced-community-care/
2. O’Donnell D, O’Shea M, Donnelly S, Ní Shé É, O’ Donoghue G, Bourke N, et al. Getting Started in Developing Core Competences for Interprofessional Collaboration Within Integrated Care Teams for Older People: A Framework for the National Integrated Care Programme for Older Persons [Internet]. ICPOP. University College Dublin; 2021 [cited 2023 May 16]. Available from: https://www.icpop.org/publications#gref

## P18: Addressing physical healthcare in mental health settings: implementation and evaluation of two novel interventions

### Raymond McGrath^1^, Julie Williams^2^, Gracie Tredget^1^, Amy Ronaldson^3^, Jorge Arias de la Torre^3^, Isabel McMullen^1^, Prashanth Reddy^4^, George Gillett^1^, Theo Boardman-Pretty^1^, Nick Sevdalis^2^, Fiona Gaughran^5^, Ioannis Bakolis^3^, Zarnie Khadjesari^6^, Euan Sadler^7^

#### ^1^South London and Maudsley NHS Foundation Trust, London, SE5 8AZ, UK; ^2^Centre for Implementation Science, King’s College London, London, SE5 8AF, UK; ^3^Department of Biostatistics and Health Informatics, King’s College London, London, SE5 8AF, UK; ^4^King’s College Hospital NHS Foundation Trust, Denmark Hill, London, SE5 9RS, UK; ^5^Psychosis Studies, King's College London, London, SE5 8AF, UK; ^6^Behavioural and Implementation Science (BIS) research group, University of East Anglia, Norwich, UK; ^7^Department of Nursing, Midwifery and Health, University of Southampton, Southampton, UK

##### **Correspondence:** Raymond McGrath (raymond.mcgrath@slam.nhs.uk)

*Implementation Science 2024*, **19(1)**:P18

**Background**

People with severe mental illness (SMI) have significantly worse physical health and are less likely to receive medical interventions compared to the general population. The IMPHS Project [1] implemented and evaluated two novel service interventions (Consultant Connect and a Physical Health Clinic) in inpatient settings at the UK’s largest Mental Health NHS Foundation Trust, during the Covid-19 pandemic.

Consultant Connect (CC) is an app-based communications platform, now available in all clinical areas in the Trust. The Physical Health Clinic is available to 12 wards, and provides a consultant physician to respond to referrals.

The interventions aim to increase support for mental health clinicians managing physical health conditions and improve integration between the mental health Trust and its partner acute Trusts.

**Methods**

Both interventions have been evaluated separately, in terms of 1) understanding the process of implementation, and 2) establishing acceptability and feasibility. Implementation activities have been logged and mapped to strategies and domains in the Expert Recommendations for Implementing Change (ERIC) framework. Data on uptake and usage has also been collected, as well as qualitative feedback from users (*n*=18).

**Results**

Statistical analysis of the implementation strategies used and how they map to uptake and usage data over the same period is currently ongoing. The mapping exercise identified 39 ERIC strategies were employed once or more, to either or both interventions, since the launch. CC has been used >2700 times, and its app has been downloaded and registered >550 times by Trust clinicians. The PHC has received >240 referrals and been used by >60 clinicians.

**Conclusion**

Integration of services is a priority for the UK health and care system and has the potential to improve health outcomes for the population as a whole. Results from this evaluation can provide insights for future novel service developments and can help to overcome implementation and sustainability challenges.

**Trial Registration:** Not applicable

**Consent to publish:** Not applicable

**Reference**

1. King’s Health Partners [Internet]. London: KHP; 2023. Integrating our mental and physical healthcare systems project (IMPHS); 2023 March [cited 2023 May 15]. Available from https://www.kingshealthpartners.org/our-work/mind-and-body/our-projects/physical-healthcare-in-severe-mental-illness

## O19: Acceptability of the CONNECTS-Food resource: supporting primary schools in implementing a systems-based whole school approach to food

### Wendy Burton^1^, Jayne V. Woodside ^2^, Harry Rutter^3^, Amir M. Sharif^4^, Charlotte E.L. Evans^5^, Suzanne Spence^6^, Sara Ahern^7^, Niamh O’ Kane^2^, Maria Bryant^1,8^, Tim Baker^9^

#### ^1^Department of Health Sciences, University of York, Heslington, York, YO10 5DD, UK; ^2^Centre for Public Health, Queens University Belfast, University Rd, Belfast, BT7 1NN,UK; ^3^Department of Social & Policy Sciences, University of Bath, Claverton Down, Bath, BA2 7AY, UK; ^4^Faculty of Management, Law and Social Sciences, University of Bradford, Richmond Road, Bradford, BD7 1DP, UK; ^5^School of Food Science and Nutrition, University of Leeds, Woodhouse, Leeds, LS2 9JT, UK; ^6^Population Health Sciences Institute, University of Newcastle, Newcastle upon Tyne, NE1 7RU, UK; ^7^Better Start Innovation Hub, Bradford Institute of Health Research, Bradford Royal Infirmary, Duckworth Lane, Bradford, BD9 6RJ, UK; ^8^Hull York Medical School, Heslington, York, YO10 5DD, UK; ^9^Independent Consultant, London, UK

##### **Correspondence:** Wendy Burton (wendy.burton@york.ac.uk)

*Implementation Science 2024*, **19(1)**:O19

**Background**

Schools promote healthy nutrition and reduce health inequalities through the implementation of whole school approaches to food (e.g., food culture, environment, and education). However, uptake of such approaches is often low. As part of the CONNECTS-Food project, an online resource was developed with key stakeholders to set out key principles of a whole school approach to food, and address barriers to implementation within the school food system. This paper explores the acceptability of this resource by schools.

**Methods**

A qualitative interview study was undertaken with 15 stakeholders (senior leaders, teachers, and kitchen staff) across six UK primary schools. Participants were asked to review the CONNECTS-Food resource before interviews, providing feedback on its acceptability. A theoretical framework of acceptability [1] was used to inform the topic guide and was used as a deductive coding framework to analyse the data using thematic analysis.

**Results**

Participants found the CONNECTS-Food resource visually appealing and easy to navigate, and felt it contained useful resources to support implementation of a whole school approach to food. Following review, the majority expressed an intention to implement small changes within their school in line with key principles, using the resource for guidance. However, all those interviewed described implementation barriers to a whole school approach to food that could deter engagement with the resource, including competing priorities, perceived lack of time, and lack of mandatory requirements for implementation. Some interviews suggested the whole school approach to food concept is misunderstood, with limited recognition.

**Conclusion**

CONNECTS-Food could be used as a tool to support implementation of a whole school approach to food. Wider changes within school food systems are needed to encourage schools to adopt the resource. Further work should focus on supporting schools in understanding what a whole school approach to food means.

**Trial Registration:** ISRCTN85297523

**Consent to publish**

Written informed consent was obtained from all participants taking part in the research.

**Reference**

1. Sekhon, M., Cartwright, M. Francis, J. BMC Health Services Research (2017) 17:88

## O20: A theory of change of a quality improvement training programme at a large London hospital

### Katie L. Richards^1^, Manuela Russo^1^, Barbora Krausova^1^, Kathryn Watson^1^, Lorraine Catt^2^, Alister Notridge^2^, Rachel Olive^1^, Kia-Chong Chua^1^, Lucy Goulding^1^, Andrea Cortes^2^, Nick Sevdalis^1,3^

#### ^1^King’s Improvement Science, Centre for Implementation Science, Health Service and Population Research Department, King’s College London, London, UK; ^2^Quality Improvement Team, King’s College Hospital NHS Foundation Trust, London, UK; ^3^Centre for Behavioural and Implementation Science Interventions, National University of Singapore, Singapore

##### **Correspondence:** Katie L. Richards (katie.1.richards@kcl.ac.uk)

*Implementation Science 2024*, **19(1)**:O20

**Background**

Organisation-wide capacity building programmes for quality improvement (QI) have been linked to higher ratings in quality assessments [1]. However, the conditions and mechanisms through which these programmes impact improvement goal(s) at scale have not always been clearly articulated. The aim of this study was to develop a Theory of Change (ToC) outlining the ultimate goals of a QI training programme and the conditions and mechanisms required to reach these goals.

**Methods**

A qualitative study informed by the Aspen Institute’s guide to ToC was conducted [2]. Twenty participants were purposively recruited, including QI team members, hospital staff, and past/present patients. Research evidence, QI training materials, and data gathered during workshops and semi-structured interviews were used to iteratively develop the ToC. Data were analysed using framework analysis.

**Results**

The ultimate goals identified during the study were improvements in QI infrastructure, a QI culture, and sustained improvements in the quality and experience of care, services and operations for patients, staff, and the wider community. Views on the goals were mixed, but many felt that they should evidence sustained improvements in care. Key conditions and mechanisms required to reach these goals included: (1) leadership supporting and enabling QI; (2) QI perceived as relevant and a priority; (3) capacity/time for training and QI; (4) QI governance; (5) staff awareness of ‘QI offer’; (6) accessibility of ‘QI offer’; (7) patients and the public co-producing QI; (8) listening to and involving staff at all levels and a diverse programme/project team; (9) appropriately using data; and (10) sharing, learning and disseminating internally and externally.

**Conclusion**

Our results suggest that the aims of the training programme should be to improve QI infrastructure, promote a QI culture, and sustain improvements in the quality of care, services and operations. Leadership support emerged as one of the most crucial conditions required to reach these goals.

**Trial Registration:** Not applicable

**Consent to publish:** Not applicable

**References**

1. Care Quality Commission. Quality improvement in hospital trusts: Sharing learning from trusts on a journey of QI. Newcastle upon Tyne (UK):Care Quality Commission; 2018. Available from https://www.cqc.org.uk/sites/default/files/20180911_QI_hospitals_FINAL.pdf
2. Anderson, AA. The Community Builder’s Approach to Theory of Change: A Practical Guide to Theory Development. New York (NY): Aspen Institute; 2006.

## O21: Improving comprehensive care: insights from a mixed method survey following the introduction of Australian comprehensive care standard

### Beibei Xiong^1^, Paul Prudon^1^, Daniel X. Bailey^1^, Christine Stirling^2^, Melinda Martin-Khan^1,3,4^

#### ^1^Centre for Health Services Research, The University of Queensland, Brisbane, Queensland, 4102, Australia; ^2^School of Nursing, University of Tasmania, Hobart, Tasmania, 7000, Australia; ^3^Department of Health and Life Sciences, University of Exeter, Exeter, England, EX1 2HZ, United Kingdom; ^4^School of Nursing, University of Northern British Columbia, Prince George, British Columbia, V2N 4Z9, Canada

##### **Correspondence:** Beibei Xiong (Beibei.Xiong@uq.edu.au)

*Implementation Science 2024*, **19(1)**:O21

**Background**

In 2019, the Australian Commission on Safety and Quality in Health Care (ACSQHC) mandated the Comprehensive Care Standard (CCS) as a means of ensuring patients receive total health care that meets their needs [1]. Health organisations use different approaches to meet the requirements of the standard, but they are measured against a common set of key indicators [2 , 3]. This project aims to examine the implementation challenges and facilitators of the CCS and the impacts of the CCS on patient care and outcomes in acute care hospitals.

**Methods**

A questionnaire was developed based on the ACSQHC’s evaluation of the CCS survey [4] and CCS implementation guide [5 , 6]. The main survey included five sections: demographics, knowledge, practices, barriers and facilitators, and perceived effects. We distributed the survey to care professionals through healthcare organisations’ and clinical networks’ websites, newsletters, emails, and social media from October 1 2022 to April 30 2023. RStudio was used for descriptive analysis, and Nvivo was used for theme analysis on text.

**Results**

We received 658 valid responses from Australian care professionals. Common implementation barriers include lack of knowledge about CCS, heavy documentation burden, staff shortage, team communication and handover gaps, and competing priorities. Common facilitators include risk screening tools in place, medical records modified to tailor CCS, CCS working groups and consumers involvement. More than half of the participants think that following the introduction of the CCS, there was an improvement in interdisciplinary collaboration, shared decision-making, and patient education, but also an increase in healthcare costs.

**Conclusions**

Integrating the existing system and process and providing extensive organizational support are needed for a successful implementation of the CCS. There is also a particular need for education and training on effective communication for shared decision-making and an interdisciplinary approach to patient risk identification and management.

**Trial Registration:** Not applicable

**Consent to publish:** Not applicable

**References**

1. Australian Commission on Safety and Quality in Health Care (ACSQHC). National Safety and Quality Health Sevice Standards. 2nd ed. Sydney: *ACSQHC*; 2021. [cited 2022 May 22]. Available from: *https://www.safetyandquality.gov.au/sites/default/files/2021-05/national_safety_and_quality_health_service_nsqhs_standards_second_edition_-_updated_may_2021.pdf*.
2. ACSQHC. Consumers and accreditation. 2020. [webpage on the internet]. [cited 2022 May 22]. Available from: *https://www.safetyandquality.gov.au/standards/national-safety-and-quality-health-service-nsqhs-standards/assessment-nsqhs-standards/consumers-and-accreditation*.
3. Murgo M, Dalli A. Australian health service organisation assessment outcome data for the first 2 years of implementing the Comprehensive Care Standard. *Australian Health Review*. 2022; 46(2): 210-6. 10.1071/ah21299
4. ACSQHC. Comprehensive Care Standard: Review of implementation. Sydney: *ACSQHC*; 2022. [cited 2022 March 1]. Available from: *https://www.safetyandquality.gov.au/sites/default/files/2022-08/comprehensive_care_standard_-_review_of_implementation.pdf*
5. ACSQHC. Implementing the Comprehensive Care Standard-Essential elements for delivering comprehensive care. Sydney: *ACSQHC*; 2018. [cited 2022 May 22]. Available from: *https://www.safetyandquality.gov.au/sites/default/files/migrated/Implementing-Comprehensive-care-Essential-Elements-Accessibility-PDF.pdf*.
6. ACSQHC. Implementing the Comprehensive Care Standard-A Conceptual Model for Supporting Comprehensive Care Delivery. Sydney: *ACSQHC*; 2018. [cited 2022 May 22]. Available from: *https://www.safetyandquality.gov.au/sites/default/files/2019-04/Conceptual-model-for-supporting-comprehensive-care-delivery.pdf*.

## P22: Scale-up of a novel vital signs alert device to improve maternity care in Sierra Leone: a mixed methods evaluation of device repair and maintenance

### Charlotte Greene^1^, Alice Pearson^1^, Alex Rideout^1^, Katy Kuhurt^1^, Daniel Gassim Kay Jah^2^, Francis Momoh^3^, Professor Andrew Shennan^1^

#### ^1^Department of Women and Children’s Health, King’s College London, London, UK; ^2^Welbodi Partnership, Freetown, Sierra Leone; ^3^Princess Christian Maternity Hospital, Ministry of Health and Sanitation, Freetown, Sierra Leone

##### **Correspondence:** Charlotte Greene (pearson.alice1@gmail.com)

*Implementation Science 2024*, **19(1)**:P22

**Background**

Sierra Leone (SL) has one of the highest rates of maternal mortality globally. The CRADLE VSA is a vital signs monitoring device and associated training package designed to enable early recognition and management of unwell pregnant women. Following a successful trial in SL which showed a 60% reduction in maternal mortality, the CRADLE device was rolled out across 8 (of 16) health districts in May 2020 - March 2021. Anecdotally there have been some reports of broken devices, and this needs further evaluation to ensure sustainability of the intervention.

**Aims**

1. To establish the proportion of CRADLE VSA devices reported as ‘broken’ and to systematically identify causes
2. To explore existing ‘maintenance and repair’ pathways to inform development of a robust maintenance strategy that can be applied at national level

**Methods**

Data was collected from five districts in SL between January-March 2023. ‘Broken’ devices were collected and categorized by problem. A selection of district health team, medical technicians and clinical staff were interviewed to explore barriers to maintenance and sustainability.

**Results**

During the national scale-up, 1257 devices were distributed amongst the 5 districts. Of these *n* = 261(20.8%) were reported ‘broken’. Allowing for devices that were working or damaged in storage, the commonest problems were cuff, *n*= 176 (75%), bulb, *n*= 100 (43%) and machine, *n*=43 (18%). 100% of interviewees were able to identify these specific problems. Barriers to repair included logistical challenges transporting broken devices, communication breakdown and high staff turnover.

**Conclusion**

The commonest problems were related to the cuff and bulb. These are cheap and easily replaced by local healthcare staff. We provided districts with spare cuffs and bulbs and produced a training video for users and medical store staff to identify and repair common problems. These simple changes could improve the sustainability of the device and facilitate long-term use.

**Trial Registration:** Not applicable

**Consent to publish:** Not applicable

## P24: Challenges for oral health promotion in the school health program in Brazil: a literature review informed by the CFIR framework

### Victoria Almeida Oliveira Furtunato^1^, Carlos Alberto dos Santos Treichel^2^

#### ^1^Department of Collective Health, School of Medical Sciences, State University of Campinas, Campinas-SP, Brazil; ²Department of Maternal-Child and Psychiatric Nursing, School of Nursing, University of São Paulo, São Paulo-SP, Brazil

##### **Correspondence:** Carlos Alberto dos Santos Treichel (treichel@usp.com)

*Implementation Science 2024*, **19(1)**:P24

**Background**

Established in 2007, the Programa de Saúde na Escola/ School Health Program (PSE) aims to contribute to the comprehensive education of Brazilian students in the public school system through health promotion, prevention and care [1]. Among the actions included in the program, are those aimed at improving oral health conditions through the participation of dentists in activities such as health education, topical application of fluoride, atraumatic restorative treatment (ART) and supervised brushing [1]. Although it presents favourable results, the implementation of the oral health component within the scope of the program is still low in several regions of the country. The objective of this study was to identify the challenges for promoting oral health in the School Health Program.

**Methods**

We conducted an integrative literature review in PubMed (Publisher Medline) and SciELO (Scientific Electronic Library Online) databases. The search took place between February and March 2022 and tracked studies published between 2007 and 2022 using the descriptors: *Programa de Saúde na Escola*; *PSE*; School Health Services; Dentistry; Dental surgeon; Dentist; Oral Health Promotion; Oral health; Preventive Dentistry. We categorized the identified challenges based on the Consolidated Framework for Implementation Research (CFIR) [2]. For this categorization, we used Minayo’s thematic categorical content analysis model [3].

**Results**

Challenges related to the internal context were the most frequent, with emphasis on those related to relationship and communication networks, and readiness for implementation. Among the most repeated challenges were the low promotion of intersectoriality and the lack of materials and adequate structure to carry out the program’s activities.

**Conclusion**

Despite a political definition at the national level, the lack of local preparation for organizing and maintaining program activities is an obstacle to promoting oral health through the program, thus demanding the use of implementation strategies to ensure its effectiveness and sustainability.

**Acknowledgments**

This study was financed in part by the Coordenação de Aperfeiçoamento de Pessoal de Nível Superior - Brasil (CAPES) - Finance Code 001.

**Trial Registration:** Not applicable

**Consent to publish:** Not applicable

**References**

1. Moreira RS, Mauricio HA, Jordão LMR, Freire MCM. Implementação do Programa Saúde na Escola: relação com aspectos da saúde bucal dos estudantes. Saúde debate. 2022; 46(spe3):166–78.
2. Damschroder LJ, Aron DC, Keith RE, et al. Fostering implementation of health services research findings into practice: a consolidated framework for advancing implementation science. Implementation Sci. 2009; 4:50.
3. Minayo MCS. O desafio do conhecimento: pesquisa qualitativa em saúde. 5. ed. São Paulo: Hucitec-Abrasco; 1998.

## O25: Exploring processes for implementing palliative care in intensive using normalisation process theory

### Stephanie A Meddick-Dyson^1^, Jason W Boland^1^, Andrew Bradshaw^2^, Mark Pearson^1^, Fliss E M Murtagh^1^

#### ^1^Wolfson Palliative Care Research Centre, Hull York Medical School, University of Hull, UK; ^2^Cicely Saunders Institute, Kings College London, London, England, United Kingdom

##### **Correspondence:** Stephanie A Meddick-Dyson (stephanie.meddick-dyson@nhs.net)

*Implementation Science 2024*, **19(1)**:O25

**Background**

If successfully implemented, palliative care interventions within Intensive Care Units (ICU) support patients and relatives in times of uncertainty and distress. This study aims to understand professional perspectives about providing palliative care within Intensive Care Units in the UK.

**Methods**

UK healthcare professionals with experience of providing or organising palliative care in the ICU were asked to complete the validated 23-item Normalisation Measure Development survey with 20 core items organised by Normalisation Process Theory constructs. Free text comments were thematically synthesised for further insight into how professionals work to provide palliative care in their ICU.

**Results**

153 completed surveys; 69% of respondents were ICU professionals, 31% were palliative care professionals. Respondents reported being familiar with palliative care in the ICU and that it was part of their normal work. Respondents had positive perspectives about implementation of palliative care in the ICU, reporting positively about coherence (sense-making work), cognitive participation (relational work) and reflexive monitoring (appraisal work). Rating of collective action (operational work) were more negatively perceived. Free-text responses revealed themes reflecting (i) professional roles within the ICU, including the significant interplay between ICU doctors and nurses, the benefits, and difficulties of specialist palliative care involvement, and the nuances of ICU care that require specialist knowledge. (ii) Timing of provision, comprising mixed perceptions of the ability to recognise the need for palliative care and how it is a routine part of ICU care. (iii) Challenges to providing palliative care in the ICU including conflicts, pressures, lack of training, and the need to avoid medicalisation of death.

**Conclusion**

The understanding and value of, and motivation for, providing palliative care in the ICU is promising. Important implementation gaps may lie within operational work. Future work is needed around resources and training to support palliative care provision and navigating the complex, but vital, interplay between multidisciplinary teams.

**Trial Registration:** Not applicable

**Consent to publish:** Not applicable

## P26: Palliative care implementation in the intensive care unit: using the implementation research logic model as a framework for systematic review and synthesis

### Stephanie A Meddick‐Dyson^1^, Jason W Boland^1^, Mark Pearson^1^, Sarah Greenley^2^, Rutendo Gambe^1^, John R Budding^1^, Fliss E M Murtagh^1^

#### ^1^Wolfson Palliative Care Research Centre, Hull York Medical School, University of Hull, Hull HU6 7RX, UK; ^2^Cancer Awareness, Screening and Diagnostic Pathways Research Group (CASP), Hull York Medical School, University of Hull, Hull, UK

##### **Correspondence:** Stephanie A Meddick‐Dyson (stephanie.meddick-dyson@nhs.net)

*Implementation Science 2024*, **19(1)**:P26

**Background**

Logic models help conceptualise and manage complexity and can provide a framework for systematic reviews [1]. The Implementation Research Logic Model (IRLM) allows examination of causal pathways and mechanisms enabling implementation [2]. This systematic review aimed to identify and synthesise knowledge on how models of integrating palliative care into the ICU have been implemented, providing critical recommendations for future development and implementation of complex interventions in the field. The IRLM has not yet been used in a systematic review. This study demonstrates the utility of the IRLM as an a priori framework for synthesis.

**Methods**

Standard systematic review methods following PRISMA guidelines. The IRLM was used as an a priori framework for synthesis of intervention characteristics, determinants, implementation strategies, mechanisms, and outcomes reported within effectiveness trials and process evaluations of palliative care interventions in the intensive care unit.

**Results**

71 effectiveness and/or feasibility studies, and 8 process evaluations referenced 66 interventions. The IRLM provided a clear framework to organise data. Consolidated Framework for Implementation Research and Expert Recommendations for Implementing Change headings formed NVivo codes for determinants of implementing palliative care interventions in the Intensive Care Unit (ICU), implementation strategies to address these, and mechanisms for how these strategies lead to change. These codes successfully captured nearly all data. Within included studies, determinants and implementation strategies were widely reported, but implementation mechanisms were not. The IRLM allowed for reporting of relationships between determinants, strategies, and mechanisms, and how these varied with intervention characteristics including ICU type and model of delivery of palliative care.

**Conclusion**

The IRLM was successfully used to guide a framework synthesis of evidence on implementation of palliative care interventions within ICUs. This methodology could be transferred to other subject areas to systematically review implementation factors. Future work is needed to understand the processes behind these strategies by use of theory.

**Trial Registration:** Not applicable

**Consent to publish:** Not applicable

**References**

1. Brunton G, Oliver S, Thomas J: Innovations in framework synthesis as a systematic review method. *Res Synth Methods* 2020, 11(3):316-330.
2. Smith JD, Li DH, Rafferty MR: The Implementation Research Logic Model: a method for planning, executing, reporting, and synthesizing implementation projects. *Implementation Science*, 15(1):84.

## P27: De-implementing low value practices in mental health care

### Qandeel Shah, Rebecca Lawton, Sarah Alderson, Ed Breckin

#### School of Psychology, University of Leeds, University Rd, Leeds, LS2 9JT, UK

##### **Correspondence:** Qandeel Shah (ll14qs@leeds.ac.uk)

*Implementation Science 2024*, **19(1)**:P27

**Background**

The use of healthcare practices with little or no benefit is a widespread problem, as evidence shows 20-25% of all healthcare provided is unnecessary or harmful. These potentially unsafe practices are known as low value care. Although there has been some progress in de-implementing low value care in primary and acute settings, little research has looked at low value practices in mental health care. With mental health services currently experiencing huge demands and a lack of resources it is has become increasingly important to de-implement low value care in this area. Therefore, this study aims to identify practices in mental health care that are potential targets for de-implementation.

**Method**

A Qualitative exploratory research design was used. 15 peer support workers were recruited from 5 different mental health charities to take part in interviews. The interviews were semi-structured and involved in-depth discussions about experiences of ineffective and wasteful care. Interviews were conducted online and lasted 30-90 minutes. The data was subject to abductive thematic network analysis which incorporates the principles of abductive theory of method, thematic network analysis, and thematic analysis.

**Findings**

The findings show 3 main practices peer support workers consider to be low value: (1) long term use of antidepressants, (2) physical restraint and (3) enhanced observations. Participants viewed these practices as ineffective, wasteful, and even harmful for the service user. They also made recommendations for how low value practices could be de-implemented. This included removing, reducing, restricting or replacing practices with more effective and safe alternatives.

**Conclusion**

This study identifies potential targets for de-implementation in mental health care from the perspective of peer support workers. De-implementing harmful or unnecessary care could help free up the vital resources needed to provide safe, high quality mental health care.

**Trial Registration:** Not applicable

**Consent to publish:** Not applicable

## O29: Physical healthcare in community mental health services for adults with Serious Mental Illness (SMI): implementing recommendations using the knowledge-to-action framework

### Gracie Tredget^1^, Julie Williams^2^, Ray McGrath^1^, Nick Sevdalis^2^, Fiona Gaughran^3^, Ioannis Bakolis^4^, Euan Sadler^5^

#### ^1^South London and Maudsley NHS Foundation Trust, London, SE5 8AZ, UK; ^2^Centre for Implementation Science, King’s College London, London, SE5 8AF, UK; ^3^Psychosis Studies, King’s College London, London, SE5 8AF, UK; ^4^Department of Biostatistics and Health Informatics, King’s College London, London, SE5 8AF, UK; ^5^Department of Nursing, Midwifery and Health, University of Southampton, Southampton, UK

##### **Correspondence:** Gracie Tredget (gracie.tredget@slam.nhs.uk)

*Implementation Science 2024*, **19(1)**:O29

**Background**

Adults with serious mental illnesses (SMI), die more prematurely from preventable physical health problems than the average population. In 2014, NICE guidance required mental health providers to complete annual physical health checks to better identify and address physical health problems amongst SMI patients. We conducted a service evaluation within a UK Mental Health Trust to investigate barriers faced regarding physical healthcare that hindered completion of checks within mental health settings. From this work, a series of recommendations were developed, that are now being translated using the Knowledge-to-Action (KTA) Framework [1] to improve physical healthcare for SMI patients across two Mental Health Trusts in south east London.

**Methods**

A service evaluation was conducted using a qualitative methodology, involving interviews (*n*=23) and focus groups (*n*=27) with mental health staff, patients, and carers. Thematic analysis was used to synthesis collected data, and reviewed through workshops with staff, patients, and carers to develop recommendations.

**Results**

23 interviews and 8 focus groups were completed (*n*=50).

4 recommendations were identified:

1. Clear organisational vision and strategy for physical healthcare
2. Accessible policy and guidelines
3. A comprehensive training programme
4. A quality framework outlining the physical healthcare offer for SMI patients

We seek to build upon these recommendations by supporting both Trusts to develop and implement them. To facilitate this, both Trusts are working together to establish a Community of Practice (COP) to share best practice. Using the KTA, we aim to achieve parity in physical healthcare practice across both Trusts for SMI patients.

**Conclusion**

We hope this work will improve physical healthcare standards in routine mental health practice, and better equip Mental Health Trusts to enhance access, care quality, and outcomes for SMI patients. We are working with both Trusts to evaluate whether these changes lead to improvements in the future.

**Trial Registration:** Not applicable

**Consent to publish:** Not applicable

**Reference**

1. Graham I, Logan J, Harrison M, Strauss S, Tetroe J, Caswell W, Robinson N: Lost in knowledge translation: time for a map? The Journal of Continuing Education in the Health Professions 2006, 26, p. 19.

## P30: Development of a video-based evidence synthesis knowledge translation resource: drawing on a user-centred design approach

### Cristian Deliv^1^, Declan Devane^2,3^, El Putnam^4^, Patricia Healy^2,3^, Amanda Hall^5^, Sarah Rosenbaum^6^, Elaine Toomey^2,3^

#### ^1^School of Medicine, University of Galway, Galway, Ireland; ^2^Cochrane Ireland and Evidence Synthesis Ireland, University of Galway, Galway, Ireland; ^3^School of Nursing and Midwifery, University of Galway, Galway, Ireland; ^4^School of English and Creative Arts, University of Galway, Galway, Ireland; ^5^Primary Healthcare Research Unit, Faculty of Medicine, Memorial University, Newfoundland, Canada; ^6^Centre for Informed Health Choices, Norwegian Institute of Public Health, Oslo, Norway

##### **Correspondence:** Elaine Toomey (Elaine.toomey@universityofgalway.ie)

*Implementation Science 2024*, **19(1)**:P30

**Link to publication**

## O31: Trusting relationships and implementation outcomes: findings from a trust-building intervention to support scale-up of an evidence-based program in child welfare

### Allison Metz^1^, Todd Jensen^1^, Amanda Farley^1^, Annette Boaz^2^, Lacy Dicharry^3^

#### ^1^School of Social Work, University of North Carolina at Chapel Hill, Chapel Hill, USA; ^2^London School of Hygiene and Tropical Medicine, London, UK; ^3^Lacy Dicharry Consulting, Baton Rouge, Louisiana, USA

##### **Correspondence:** Allison Metz (allison.metz@unc.edu)

*Implementation Science 2024*, **19(1)**:O31

**Background**

The research aim is to assess the feasibility and acceptability of developing and delivering a training and coaching intervention with implementation teams to build team cohesion, psychological safety, and trust, in order to increase capability, opportunity, and motivation to use evidence, and to enhance commitment and resilience for implementation. The setting is a public child welfare system in the United States implementing a statewide, evidence-based peer-to-peer mentoring model for youth in foster care. Implementation teams include service providers, public system leadership, and youth.

**Methods**

This study employs mixed-methods with a single-case design component. Participants consider hypothesized mechanisms (capability, opportunity, motivation; commitment and resilience) linking trust with improved implementation. Our analytic sample was comprised of 15 individuals (88 total observations; average of 5.9 data-points per participant) who participated in the full course of trust-building training activities. We employed multilevel mixed-effects linear regression to assess change over time in participants’ (a) perceptions that team members trusted them (8 items; α = 0.91) and (b) reports of their own trust toward team members (8 items; 𝛼 = 0.86). We also completed and qualitatively analyzed in-depth interviews (*n*=7).

**Results**

On average, participants reported significant increases over time in their perceptions that they were trusted by their team (b = 0.31 units, *p* < .05). In addition, on average, participants reported statistically negligible increases over time in the trust they had for their team (b = 0.07 units, *p* = .63). Results from the qualitative analysis foregrounded themes related to addressing power differentials, making space for trust building, and the contribution of trust to commitment and motivation for implementation.

**Conclusion**

This study demonstrates the feasibility of implementing a trust building intervention and developing skills of implementation stakeholders to foster trust among each other. Findings also emphasize the role of trust in contributing to implementation progress in complex systems. [1]

**Trial Registration:** Not applicable

**Consent to publish:** Yes

**Reference**

1. Metz A, Jensen T, Farley A, Boaz A. Is implementation research out of step with implementation practice? Pathways to effective implementation support over the last decade. Implementation Research and Practice. 2022 Jun;3:26334895221105585.

## P32: Views and experiences of mental health services implementation stakeholders in Ukraine after the onset of the current full-scale war

### Alyona Mazhnaya^1^, Beth McGinty^2^, Sergiy Bogdanov^3^

#### ^1^School of Public Health, National University of Kyiv-Mohyla academy, Kyiv, 04655, Ukraine; ^2^Population of Health Sciences, Weill Cornell Medicine, New York, NY, 10065, USA; ^3^Centre for Mental Health and Psychosocial Support, National University of Kyiv-Mohyla academy, Kyiv, 04655, Ukraine

##### **Correspondence:** Alyona Mazhnaya (a.mazhnaia@ukma.edu.ua)

*Implementation Science 2024*, **19(1)**:P32

**Background**

According to the WHO situation report [1], approximately 18 million people have been affected by the escalation of the war in Ukraine since February 24, 2022. As the war continues, the population of Ukraine continues to experience acute psychological distress, exacerbation of chronic mental health problems, and socioeconomic effects imposed by the war. In contrast, access to psychological and psychiatric support is limited. As the healthcare system has been stretched beyond capacity, multiple local and international organisations have mobilised to provide mental health services for various target groups and in many locations using different modalities. A steep increase in the number of service providers and the type of services that followed presents both: the opportunity and challenges from the perspective of scaling up mental health services to meet the needs and be sustainable. The gap remains in documenting, systematising, and analysing the implementation landscape for mental health services in Ukraine and humanitarian settings.

**Methods**

We conducted semi-structured interviews to gather insights about perceived features of outer context, bridging factors, and preparation and implementation of interventions according to the EPIS framework [2]. Data collection is ongoing, with a target sample size of up to 30 participants. Completed interviews include 8 participants who are mental health service providers working on regional, national, and international levels directly providing mental health services, organising them, or funding. Data were analysed thematically by identifying deductive (stemming from EPIS framework) and inductive themes.

**Results**

Several salient themes originated during analysis: recognition of mental health as a critical current and future area of public health in Ukraine, changing needs and services, and challenges to coordination and strategy-based programming. The most salient theme is the shared understanding of the critical role of mental health services for the population and the government’s commitment to reforming mental health services in Ukraine. Participants described the changing needs since the full-scale invasion, and service providers have been adapting programs and services to meet those needs. The data also highlights the need to build partnerships and find a place in the national mental health service provision system. Partnerships and referrals are largely based on ad-hoc collaborations and a need to solve particular -programmatic/project goals. Initiatives, projects, programs, volunteer efforts and state-funded services are challenging to coordinate and navigate for the end user.

**Conclusion**

Currently and in the future, mental health is a central public health issue in Ukraine. Programs and services are changing in response to needs. However, they need to be integrated into a mental health government strategy to improve experiences for the end user.

**Trial Registration:** Not applicable

**Consent to publish:** I provide consent to publish this abstract on behalf of co-authors.

**References**

1. WHO. War in Ukraine: situation report from WHO Ukraine country office. Issue No.48, March 22 2023. In: WHO, editor. War in Ukraine. https://www.who.int/europe/publications/i/item/WHO-EURO-2023-5319-45083-68790: WHO/EUROPE; 2023.
2. Moullin JC, Dickson KS, Stadnick NA, Rabin B, Aarons GA. Systematic review of the Exploration, Preparation, Implementation, Sustainment (EPIS) framework. Implementation Science. 2019;14(1):1.

## O33: A co-produced web-based implementation toolkit to facilitate adaptive implementation in health and social care

### Cindy Brooks^1,2^, Susi Lund^2^, David Kryl^1,3^, Michelle Myall^1,2^

#### 1NIHR ARC Wessex, University of Southampton, Southampton, UK; ^2^School of Health Sciences, University of Southampton, Southampton, UK; ^3^Wessex Academic Health Science Network, Southampton Science Park, Hampshire, UK

##### **Correspondence:** Cindy Brooks (C.F.Brooks@soton.ac.uk)

*Implementation Science 2024*, **19(1)**:O33

**Introduction**

The complexity of implementing innovations across health and social care has been compounded by Covid-19, resulting in rapid, multifactorial changes [1,2,3]. While many implementation models, frameworks and tools are available [4,5], issues with design, accessibility and being tailored to specific audiences have limited opportunities for adoption [6-11]. To address these limitations, we propose adoption of a co-produced web-based implementation toolkit (WIT). WIT offers helpful, accessible and usable tools for a range of user groups to facilitate adaptive implementation across health and social care [12].

**Methods**

A mixed method survey (*n*=31), with stakeholders including health and social care professionals, public contributors, academics and third sector organisation representatives confirmed there was a need for the toolkit. Online interactive workshops with stakeholders from across these sectors were held to co-produce WIT. An evaluation of WIT is currently underway.

**Results**

WIT is designed to support adaptive implementation; focusing on early consideration of implementation factors to afford a flexible and dynamic approach, prioritising both what needs to be considered and how to operationalise this. It comprises of three components; an interactive implementation wheel, checklist and webinars. Consistent to all are six domains (Figure 1). Preliminary evaluation findings demonstrate WIT’s potential to support implementation at an early stage within health and social care settings.

**Conclusion**

Given the complexity of implementation within health and social care settings, WIT offers valuable user-centred tools to afford a flexible and adaptive approach to support implementation in dynamic and rapidly changing health and social care contexts.

**Trial Registration:** Not applicable

**Consent to publish:** Not applicable

**References**

1. Braithwaite J. Changing how we think about healthcare improvement. BMJ. 2018; doi:10.1136/bmj.k2014
2. Wensing M, Sales A, Armstrong R, Wilson P. Implementation science in times of covid-19. Implementation Science. 2020;15(1). doi:10.1186/s13012-020-01006-x.
3. Bustos, TA and Sridhar, A. What is Implementation Science? How can it guide rapid responses to COVID-19? (2021). Available at: What Is Implementation Science? | Psychology Today, United Kingdom
4. Villalobos Dintrans P, Bossert TJ, Sherry J, Kruk ME. A synthesis of implementation science frameworks and application to Global Health Gaps. Global Health Research and Policy. 2019;4(1). doi:10.1186/s41256-019-0115-1.
5. Lau R, Stevenson F, Ong BN, Dziedzic K, Treweek S, Eldridge S, et al. Achieving change in primary care—causes of the evidence to practice gap: Systematic reviews of reviews. Implementation Science. 2015;11(1). doi:10.1186/s13012-016-0396-4.
6. Lennox L, Doyle C, Reed JE, Bell D. What makes a sustainability tool valuable, practical and useful in real-world healthcare practice? A mixed-methods study on the development of the long term success tool in northwest London. BMJ Open. 2017;7(9). doi:10.1136/bmjopen-2016-014417.
7. Shediac-Rizkallah MC, Bone LR. Planning for the sustainability of community-based health programs: Conceptual Frameworks and future directions for research, practice and policy. Health Education Research. 1998;13(1):87–108. doi:10.1093/her/13.1.87.
8. Doyle C, Howe C, Woodcock T, Myron R, Phekoo K, McNicholas C, et al. Making change last: Applying the NHS Institute for Innovation and Improvement Sustainability Model to healthcare improvement. Implementation Science. 2013;8(1). doi:10.1186/1748-5908-8-127.
9. Feldstein AC, Glasgow RE. A practical, robust implementation and sustainability model (PRISM) for integrating research findings into practice. The Joint Commission Journal on Quality and Patient Safety. 2008;34(4):228–43. doi:10.1016/s1553-7250(08)34030-6.
10. Aarons GA, Hurlburt M, Horwitz SM. Advancing a conceptual model of evidence-based practice implementation in public service sectors. Administration and Policy in Mental Health and Mental Health Services Research. 2010;38(1):4–23. doi:10.1007/s10488-010-0327-7.
11. Hull L, Goulding L, Khadjesari Z, Davis R, Healey A, Bakolis I, et al. Designing high-quality implementation research: Development, application, feasibility and preliminary evaluation of the Implementation Science Research Development (ImpRes) tool and guide. Implementation Science. 2019;14(1). doi:10.1186/s13012-019-0897-z.
12. Brooks, CF, Lund, S, Kryl, D, and Myall M. Web-based Implementation Toolkit (WIT). NIHR ARC Wessex and University of Southampton. (2023) Available at: https://implementationtoolkit.org.uk/.

**Fig. 1 (Abstract O33).**
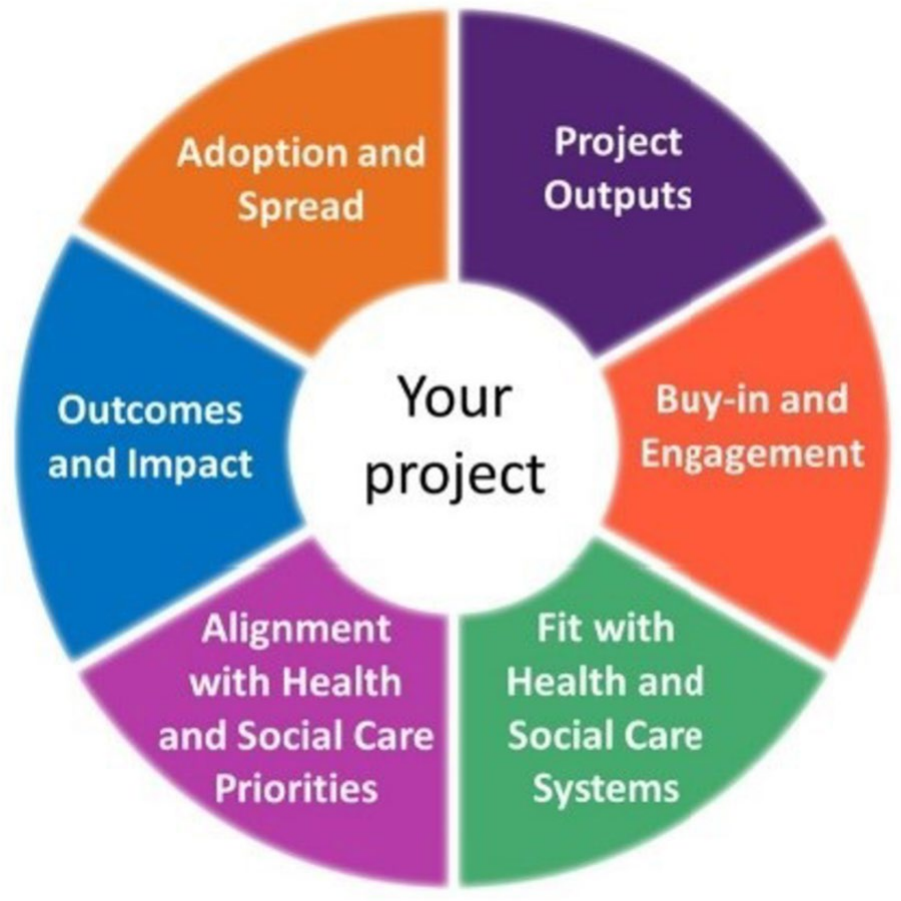
Six domains featuring in the web-based implementation toolkit

## P34: Navigating uncertainty in the implementation of a compassionate care initiative in a NHS mental health setting during Covid-19: findings from a case study with implications for sustainability

### Cindy Brooks^1,2^, Jackie Bridges^1,2^, Jane Frankland^1,2^, Michelle Myall^1,2^

#### ^1^NIHR ARC Wessex, University of Southampton; ^2^School of Health Sciences, University of Southampton

##### **Correspondence:** Cindy Brooks (C.F.Brooks@soton.ac.uk)

*Implementation Science 2024*, **19(1)**:P34

**Background**

This paper presents findings from a novel theoretically informed agency-structure study involving implementation of a Compassionate Care Initiative (CCI) in a NHS mental health setting during Covid-19 in the UK. We argue that implementation during Covid-19, not only compounded existing barriers to implementation identified in earlier studies reporting on implementation of CCI in acute hospital settings, such as staffing levels and working practices [1,2,3], but presented an unprecedented implementation landscape of uncertainty operating at micro, meso and macro levels with key implications for conceptualising sustainability.

**Method**

The study adopted a longitudinal case study design [4] in one NHS mental health setting in the UK involving semi-structured interviews with staff involved in the implementation of CCI (managers, facilitators and frontline care staff including registered nurses), alongside documentary analysis of key documents. A theoretically informed approach, involving a combination of structuration theory (ST) and Normalisation Process Theory (NPT) informed analysis [5,6,7,8].

**Results**

Findings demonstrate that the implementation of CCI during Covid-19 presented an unprecedented landscape for implementation, requiring staff navigation of complex and shifting micro, meso and macro dynamics and uncertainties. Tensions between the opportunity of CCI related activities to afford mechanisms of support to staff were counteracted with concerns over involvement through potential risks of contracting Covid-19 and wider infection control policies. Understanding staff perceptions and experiences of the complexities of implementation are pivotal to affording insight into this uncertainty.

**Conclusion**

Navigating uncertainty in the implementation of CCI during Covid-19 provides invaluable insight into the often contradictory dynamics of implementation in highly adaptive circumstances. It prioritises the importance of understanding the perceptions and experiences of those at the forefront of this agency and structure interface, conceptualising sustainability as a fluid and dynamic space to be continually revisited in accordance with these dynamics.

**Trial Registration:** Not applicable

**Consent to publish:** Not applicable

**References**

1. Bridges J, Fuller A. Creating Learning Environments for Compassionate Care (CLECC): a programme to promote compassionate care by health and social care teams. *Int J Older People Nurs,* 2015;10(1):48-58.
2. Bridges J, May CR, Fuller A, et al. Optimising impact and sustainability: a qualitative process evaluation of a complex intervention targeted at compassionate care. *BMJ Quality & Safety,* 2017;26(12):970-77.
3. Bridges J, Lee K, Griffiths P, et al. Creating Learning Environments for Compassionate Care (CLECC): The implementation and evaluation of a sustainable team-based workplace learning intervention. Southampton: University of Southampton, 2019.
4. Yin, R.K. Case study research and applications: design and methods. Thousand Oaks; 2018.
5. May, C and Finch, T. Implementing, embedding, and integrating practices: an outline of normalization process theory. Sociology. 2009: 43(3):535-54.
6. Giddens, A. The constitution of society: Outline of the theory of structuration. Cambridge: Polity Press; 1984.
7. Stones R. *Structuration Theory*. Basingstoke: Palgrave-Macmillan, 2005.
8. Brooks, C, Bridges, J, Frankland, J, Burchett, C and Myall, M. Advocating an agency-structure approach to examine implementation of a compassionate care initiative in mental health settings during Covid-19 in the UK. Proceedings of the 4th UK Implementation Science Research Conference. *Implementation Sci* 16 (Suppl 2), 104, 2021.

## P35: Content validity indexes assigned to the Brazilian version of the ImpRes-tool: a tool to improve the quality of implementation projects and research

### Carlos Alberto dos Santos Treichel^1^, Leidy Janeth Erazo Chavez^2^, Louise Hull^3^, Rosana Teresa Onocko Campos^2^

#### ¹Department of Maternal-Child and Psychiatric Nursing, School of Nursing, University of São Paulo, São Paulo-SP, Brazil; ^2^Department of Collective Health, School of Medical Sciences, State University of Campinas, Campinas-SP, Brazil; ^3^Centre for Implementation Science, Health Service and Population Research Department, Institute of Psychiatry, Psychology and Neuroscience, King’s College London, London, UK

##### **Correspondence:** Carlos Alberto dos Santos Treichel (treichelcarlos@gmail.com)

*Implementation Science 2024*, **19(1)**:P35

**Background**

Implementation Science is still very much a novel field in Brazil. No resources to guide the design of implementation research and real-world implementation projects exist that have been developed or adapted for use in Brazil. In addressing this gap, we translate and cross-culturally adapt and validate the Implementation Science Research development (ImpRes) tool and its complementary guide, previously developed in the United Kingdom. ImpRes contains ten domains that, according to the current literature and expert consensus, cover the core elements of implementation research that should be considered in the preparation of implementation research and projects [1]. The aim of this work was to appraise the content validity of the ImpRes-BR tool.

**Methods**

After the stages of translation, back-translation, and pilot testing, the ImpRes-BR tool and guide were reviewed by an expert panel, consisting of specialists in the field of applied health research composed of 10 members. Based on the experts’ responses, who rated the items on a four-point Likert scale, the content validity index at the item level (CVI-I) and at the scale level (CVI-E) was calculated using the mean calculation method (CVI-E/Med) [2]. A CVI-I of 0.78 and a CVI-E of 0.90 were defined as minimum acceptable indices [3,4].

**Results**

In addition to conceptual validity indices greater than 90%, a CVI-I of at least 0.90 was observed in all domains of the tool and its guide and an IVC-E of 0.98, thus exceeding the limits of CVI-I: 0.78 and CVI-E: 0.90 necessaries for its validity.

**Conclusion**

The Brazilian version of the ImpRes-tool and guide showed good content validity indices, both at the item level and at the scale level, thus demonstrating its usability to guide project design and implementation research in the Brazilian context.

**Acknowledgments**

This work was supported by the São Paulo Research Foundation (FAPESP) through the grants nº 2020/14309-7 and 2021/14248-0.

**Trial Registration** Not applicable

**Consent to publish** Not applicable

**References**

1. Hull L, Goulding L, Khadjesar Z, Davis R, Healey A, Bakolis I, Sevdalis N. Designing high-quality implementation research: development, application, feasibility and preliminary evaluation of the implementation science research development (ImpRes) tool and guide. Implementation Sci. 2019;14:80.
2. Sousa VD, Rojjanasrirat W. Translation, adaptation and validation of instruments or scales for use in cross‐cultural health care research: a clear and user‐friendly guideline. J Eval Clin Pract. 2011;17(2):268-74.
3. Lynn MR. Determination and quantification of content validity. Nursing Research. 1986;35 (6), 382– 385.
4. Waltz CF, Strickland OL, Lenz ER. Measurement in Nursing and Health Research, 3rd edn. New York: Springer Publishing Company, 2005.

## O36: How do teams tailor improvements in diabetes care: preliminary findings from a process evaluation study

### Elaine O’Halloran, Melissa Girling, Michael Sykes, Tracy Finch (on behalf of the EQUIPD team)

#### Department of Nursing, Midwifery & Health, Northumbria University, Newcastle upon Tyne, NE7 7XA, UK

##### **Correspondence:** Elaine O’Halloran (elaine.ohalloran@northumbria.ac.uk)

*Implementation Science 2024*, **19(1)**:O36

**Background**

NICE guidelines recommend insulin pump therapy for the treatment of Type 1 Diabetes patients with HbA1c above 69mmol/mol [1]. There are about 60,000 patients that meet these criteria but who do not use a pump [2] and significant variation by deprivation, ethnicity, sex, and location [1]. Much of this variation is likely to be attributable to staff and local organisational factors [3].

Diabetes services across England and Wales were invited to participate in a trial evaluating the effectiveness of a Quality Improvement Collaborative (QIC) aligned to the National Diabetes Audit to increase the use of insulin pumps. The QIC supports diabetes specialist teams to select, and generate commitment for, improvement actions aligned to their local influences and contexts. Within the QIC, the Theoretical Domains Framework [4] is used by clinical teams to identify influences upon care. Teams then undertake a virtual logic model exercise to align improvement strategies to these influences. We aim to describe how teams enact tailoring.

**Methods**

We use observations, documentary analysis and semi-structured interviews to explore how teams undertake tailoring work during the initial workshops and throughout the 15-month QIC. We categorise the selected and enacted improvement actions using the Expert Recommendations for Implementing Change (ERIC) [5].

**Results**

Preliminary findings from the QIC workshops describing the links between the diabetes care pathway, identified influences and proposed improvement strategies will be presented. Influences relate to patient (e.g., skills, emotion), staff (e.g., motivation, beliefs about capacity) and contextual factors (e.g., environmental context, social influences).

**Conclusions**

Exploring how teams identify the factors that influence their practice, and how and why these influences link to the strategies selected by teams to improve quality in their local contexts will support our understanding of the effectiveness of tailoring in complex interventions.

**Trial registration**: ISCRTN82176651

**Consent to publish:** Not applicable

**References**

1. National Diabetes Audit, 2019-20, Type 1 Diabetes. NHS Digital, Leeds. 2021. Available from: https://digital.nhs.uk/data-and-information/publications/statistical/national-diabetes-audit/national-diabetes-audit-2019-20-type-1-diabetes
2. National Diabetes Audit. Unpublished.
3. Llewellyn S, Procter R, Harvey G, Maniatopoulos G, Boyd A. The insulin pump therapy case study. In Facilitating technology adoption in the NHS: negotiating the organisational and policy context – a qualitative study. Health Services and Delivery Research. 2014 Jul;2(23):49-60.
4. Atkins L, Francis J, Islam R, O’Connor D, Patey A, Ivers N, et al. A guide to using the Theoretical Domains Framework of behaviour change to investigate implementation problems. Implementation Science. 2017 Dec;12(1):1-18.
5. Powell BJ, Waltz TJ, Chinman MJ, Damschroder LJ, Smith JL, Matthieu MM, et al. A refined compilation of implementation strategies: results from the Expert Recommendations for Implementing Change (ERIC) project. Implementation Science. 2015 Dec;10(1):1-14.

## O37: Hearing for all: design and delivery of a sustainable auditory implant programme

### Andile L Sibiya^1,2^, Ayanda Gina^3^, Bianca Birdsey^4^, Zandile Shezi^3^

#### ^1^Discipline of Otorhinolaryngology, School of Clinical Medicine, College of Health Sciences, University of KwaZulu-Natal, Durban, South Africa; ^2^Department of Health, KwaZulu-Natal, South Africa; ^3^Discipline of Audiology, School of Health Sciences, College of Health Sciences, University of KwaZulu-Natal, Durban, South Africa; ^4^THRIVE Parent Support & Advocacy Group, South Africa

##### **Correspondence:** Andile L Sibiya (sibiyaL1@ukzn.ac.za)

*Implementation Science 2024*, **19(1)**:O37

**Background**

The sustainability of cochlear implant programmes in LMIC’s is threatened by contextual and ecological system factors. Hearing loss is the most common sensory disability and the third greatest contributor to the global burden of disease [1,2]. Communication disorders resulting from untreated hearing loss significantly contribute to poverty in LMICs [2]. KwaZulu-Natal (KZN) is the second most populous province in South Africa and the third poorest province in the country [3]. Until 2021, KZN was the only large province without a public sector implant programme.

**Materials and methods**

This paper uses the Dynamic Sustainability Framework (DSF) to describe this programmes design and initial delivery; namely, the fit between Intervention, Practice Setting and Ecological System [4]. A task team consisting of an ENT surgeon, rehabilitationist, audiologist, and Activist for Deaf children, designed a contextually relevant model for service delivery within KZN. Qualitative methods using a case study approach were adopted. Unstructured interviews were conducted with purposively selected existing state programmes affiliated with the South African Cochlear Implant Group (SACIG). Data was analysed using thematic analysis.

**Results**

Pre-emptive and iterative consideration of the intervention, context and ecological characteristics enabled rapid delivery of the programme. Within one year of launch, the programme had developed a team with three implanting surgeons, three specialized Audiologists, as well as a network of radiologists, psychologists, social workers and paediatricians.  In the first year alone, the team had successfully implanted 8 patients, with a growing number on the waiting list.

**Conclusions**

The programme remains a sustainable entity despite staff and mentor emigration; hospital management turnover; prohibitive exchange rate fluctuations; and even major changes in the political landscape. Rational use of limited public health resources was considered at all stages of design and delivery. The sustainability rests in the intentional design that took place where change was considered inevitable, and ongoing responsible patient care was non-negotiable.

**Trial Registration:** Not applicable

**Consent to publish:** This study uses data from the data from the Kwazulu-Natal Otorhinolaryngology Database and has UKZN BREC ethics approval [Class Approval (BREC/00002826/2021)]

**References**

1. Haile LM, Kamenov K, Briant PS, Orji AU, Steinmetz JD, Abdoli A, Abdollahi M, Abu-Gharbieh E, Afshin A, Ahmed H, Rashid TA. Hearing loss prevalence and years lived with disability, 1990–2019: findings from the Global Burden of Disease Study 2019. The Lancet. 2021 Mar 13;397(10278):996-1009.
2. World Health Organization. World report on hearing. World Health Organization; 2021 Mar 3.
3. KwaZulu-Natal Department of Health. KZN DOH Strategic Plan 2020-2025 Natalia; 2020. Available from: https://www.kznhealth.gov.za/Strategic-plan-2020-2025-rev.pdf
4. Chambers DA, Glasgow RE, Stange KC. The dynamic sustainability framework: addressing the paradox of sustainment amid ongoing change. Implementation science. 2013 Dec;8(1):1-1.

## O38: Implementation of the ‘Continuity of Care’ concept through team-based care: lessons learned

### Chirk Jenn Ng^1,2^, Swetha S Kumar^1^, Prawira Oka^1,2^, Loke Chui Yee^1^, Ng Lok Pui^1^

#### ^1^SingHealth Polyclinics, Singapore 150167; ^2^Duke-NUS Medical school, Singapore 169857

##### **Correspondence:** Chirk Jenn Ng (ng.chirk.jenn@singhealth.com.sg)

*Implementation Science 2024*, **19(1)**:O38

**Background**

Continuity of care (COC) has been proven to be effective in improving the patient-doctor relationship and patient health outcomes. [1] This study was based on a pilot study that aimed to enhance COC in a Singapore public primary care setting by transforming the clinics from a ‘one patient, one clinic’ to a ‘one patient, one team’ model. The study aimed to identify the barriers and facilitators to implementing this new model of care.

**Methods**

A qualitative study was conducted among 15 doctors, 6 nurses, 6 health pals, 12 management team members and 7 patients in two polyclinics between January and April 2023. A total of 46 in-depth interviews were conducted using interview guides. The interviews were audio-recorded, transcribed verbatim and analysed using a thematic approach. The NVivo software was used to manage the data.

**Results**

This study found that while patients, healthcare providers and management recognised the importance of COC, incorporating the concept into the existing clinical care pathway was found to be challenging. Three main themes emerged: team size, team stability and information technology (IT) support. Manpower shortages and the provision of concurrent services resulted in difficulties in implementing the initial planned smaller team size and composition of 4 doctors, 2 nurses and 2 care coordinators. Additionally, COC was further impacted by the lack of stability within the care teams, due to the manpower movement across clinics and leaves. Finally, backend IT restructuring required significant time and user familiarisation to proficiently tag patients to a team and displaying it clearly on the electronic records.

**Conclusion**

Institutional support and prioritization of the new model of care are critical in ensuring its successful implementation, as this requires the institution to address existing systemic challenges, such as IT restructuring as well as increase or reshuffling of manpower.

**Trial Registration:** Not applicable

**Consent to publish:** We have obtained consent from all authors to publish.

**Reference**

1. Cabana MD, Jee SH. Does continuity of care improve patient outcomes? J Fam Pract. 2004 Dec;53(12):974-80.

## O39: Process evaluation of the implementation of the ‘Health Pals’ concept in delivering preventive care: a qualitative study

### Swetha S Kumar^1^, Prawira Oka^1,2^, Ng Lok Pui^1^, Loke Chui Yee^1^, Ng Chirk Jenn^1,2^

#### ^1^SingHealth Polyclinics, Singapore 150167; ^2^Duke-NUS Medical school, Singapore 169857

##### **Correspondence:** Swetha S Kumar (saravana.kumar.swetha@singhealth.com.sg)

*Implementation Science 2024*, **19(1)**:O39

**Background**

Preventive care is often neglected in primary care due to high patient volume and limited consultation time. [1] A proven model to overcome these challenges is to train non-clinical staff (‘health pal’) to deliver preventive care. [2] Since July 2022, a Singapore public primary care institution pilot tested the ‘health pal’ model by training existing patient service associates to provide preventive care for patients with chronic diseases. As part of process evaluation, this study aimed to explore challenges faced by health pals when performing this new role.

**Methods**

A qualitative study was conducted in two polyclinics from January to April 2023. A total of 6 health pals, 12 management team members and 7 patients participated in 25 in-depth interviews. Two researchers conducted interviews using semi-structured guides, which were audio-recorded, transcribed verbatim and checked. The data was managed using NVivo software and analysed thematically. Additionally, detailed field notes were taken during direct observation of four consultations between health pals and patients.

**Results**

While health pals welcomed their new clinical role, they faced some challenges in task execution. Despite their initial training, the health pals expressed the need for initial on-site “hand-holding”, and refresher courses as new patient queries emerged during implementation. Care delivery was hampered by their limited access to electronic medical records, resulting in an inability to obtain the patient’s full medical history. Direct observation revealed that although health pals were confident in assessing patients’ needs and offering screening and immunization, they were less confident in explaining the procedures when asked by patients.

**Conclusion**

This study highlights the importance of continuous training and support when transitioning non-clinical staff to undertake a clinical role. Determining the level of access to the electronic medical record is essential to empower the health pal to deliver appropriate preventive care while ensuring patient confidentiality.

**Trial Registration:** Not applicable

**Consent to publish:** Not applicable

**References**

1. Dexheimer JW, Talbot TR, Sanders DL, Rosenbloom ST, Aronsky D. Prompting clinicians about preventive care measures: a systematic review of randomized controlled trials. J Am Med Inform Assoc. 2008 May-Jun; 15(3):311-20.
2. Fowler T, Garr D, Mager NDP, Stanley J. Enhancing primary care and preventive services through Interprofessional practice and education. Isr J Health Policy Res. 2020 Mar 23; 9(1):12.

## O40: Facilitating the implementation of unscheduled care coordination hubs using tests of change and a rapid, relevant and responsive approach to evaluation

### Kristian Hudson^1^, Zuneera Khurshid^2^

#### ^1^The Yorkshire Humber Applied Research Collaboration, Bradford Institute for Health Research, Temple Bank House, Bradford Royal Infirmary, BD9 6RJ, UK; ^2^The Improvement Academy, Bradford Institute for Health Research, Temple Bank House, Bradford Royal Infirmary, BD9 6RJ, UK

##### **Correspondence:** Kristian Hudson (kristian.hudson@yhia.nhs.uk)

*Implementation Science 2024*, **19(1)**:O40

**Background**

Unscheduled care coordination hubs could be a potential solution to overburdened ambulance demand and pressures on accident and emergency (A&E) departments in the UK. Implementation science principles along with a ‘tests of change’ improvement practice was used to implement a care hub. A rapid, relevant and responsive evaluation was carried out to evaluate implementation, generate transportable findings and facilitate ‘within system learning’.

**Methods**

A process of engagement was followed by an initial 5-day test of change. This was then followed by three one-month tests of change. For the evaluation rapid qualitative analysis techniques (Stanford Lightning reports) were used to capture ‘within system learning’ that occurred across the test of change period. Baseline and end-of-study interviews were also conducted. Results were consolidated into transportable findings and the Consolidated Framework for Implementation Research was used to understand multi-level contextual determinants of implementation.

**Results**

The initial engagement period and the tests of change proved to be an effective approach to implementation. The evaluation proved to be useful in capturing ‘within system learning’ and producing transportable findings. It also facilitated the implementation effort. High tension for change, external change agent, key stakeholder engagement and having an ambulance member present in the care hub were strong facilitators of implementation. Commitments, ownership and governance, learning environment, reflecting and evaluation and political drivers had mixed or negative effects on implementation. Lightning reports proved useful to both researchers and the unscheduled care coordination hub team.

**Conclusion**

The combination of a ‘test of change’ and implementation science evaluation offered an effective approach to implementing and evaluating unscheduled care provision. Engagement seems to be an important precursor to this approach. Implementation researchers should move from traditional, top-down research approaches to participatory and embedded implementation research evaluations in order to close the gap between implementation research and implementation practice.

**Trial Registration**: Not applicable

**Consent to publish:** Not applicable

## O41: Using the Exploration, Preparation, Implementation, Sustainment (EPIS) framework to adapt a sexual and reproductive health intervention for Latina teens and female caregivers

### Katherine G. Merrill^1^, Jacqueline Silva^1^, Jacqueline Fuentes^1^, Angela Sedeño^2^, Susana Salgado^3^, Sara Vargas^2^, Jennifer Cano^3^, Veronica Nabor^3^, Jamison Merrill^4^, Jeff DeCelles^5^, Kate Guastaferro^6^, Geri Donenberg^1^

#### ^1^Center for Dissemination and Implementation Science, University of Illinois, Chicago, USA; ^2^The Kedzie Center, Chicago, USA; ^3^Centro Romero, Chicago, USA; ^4^School of Public Health, University of Illinois Chicago, Chicago, USA; ^5^Grassroot Soccer, Norwich, USA; ^6^New York University, New York. USA

##### **Correspondence:** Katherine G. Merrill (kgm@uic.edu)

*Implementation Science 2024*, **19(1)**:O41

**Background**

Latina teens are disproportionately impacted by adverse outcomes of risky sexual behavior. IMARA (Informed, Motivated, Aware, and Responsible Adolescents and Adults) is an evidence-based sexual health program for Black teen girls and their mothers. We set out to adapt IMARA for Latina teens and their female caregivers (FCs) (e.g., mothers, aunts) using the Exploration, Preparation, Implementation, Sustainment (EPIS) and Escoffery’s intervention adaptation frameworks.

**Methods**

In the *Exploration* phase, we conducted 6 focus groups (2 with Latina teens, 2 with FCs, 2 with staff from community partner organizations (CPOs)) and a scoping review of evidence-based sexual and reproductive health (SRH) programs for Latina teens and families to assess the potential fit of IMARA for Latinas and identify key curriculum constructs to include. In the *Preparation* phase, we conducted 6 additional focus groups (1 with Latina teens, 1 with FCs, 3 with CPO staff, 1 with original IMARA facilitators) and 7 key informant interviews to determine how to implement and sustain the adapted program. Lastly, we theater-tested the adapted program with 5 Latina teen-FC dyads over two days.

**Results**

*Exploration* phase findings revealed positive perceptions among all stakeholders of a SRH program to help Latina teens and FCs communicate about “taboo” topics in Latino culture. The scoping review identified 17 evidence-based SRH programs out of 3,970 studies screened. None targeted Latina teens and FC, reinforcing our decision to adapt IMARA. The 17 programs informed content to address in the adapted program (e.g., unplanned pregnancy). *Preparation* phase findings revealed how, when, and where to implement the program and sustainability ideas. Latina teens and FCs provided detailed feedback on curriculum content during theater testing.

**Conclusions**

Findings from the *Exploration* and *Preparation* phases will inform *Implementation* of the adapted intervention in a pilot optimization trial using the multiphase optimization strategy (MOST) framework and plans for *Sustainment*.

**Trial Registration:** Not applicable

**Consent to publish:** Not applicable

## P43: Stakeholder’s experiences of tailoring implementation of the DAFNE structured education programme for type 1 diabetes

### Jane Murphy^1^, Fiona Riordan^1^, Claire Kerins^2^, Laura-Jane McCarthy^1^, Luke Wolfenden^3^, Sheena M. McHugh^1^

#### ^1^School of Public Health, University College Cork, Cork, Ireland; ^2^Health Promotion, University of Galway, Galway, Ireland; ^3^School of Medicine and Public Health, College of Health, Medicine, and Wellbeing, the University of Newcastle, Callaghan, NSW, Australia

##### **Correspondence:** Fiona Riordan (fiona.riordan@ucc.ie)

*Implementation Science 2024*, **19(1)**:P43

**Background**

Tailored implementation strategies effectively support implementation of interventions in healthcare. However, it is unknown which tailoring approaches are most feasible and acceptable to stakeholders and which outcomes they consider important. Dose Adjustment for Normal Eating (DAFNE) is an evidence-based patient education programme recommended for type 1 diabetes management, however its implementation and how best to support delivery are underexplored. Using DAFNE as a case study, we evaluated clinical stakeholder’s experiences of the process to tailor strategies to support programme implementation.

**Methods**

DAFNE clinical teams participated in a tailoring process involving three group discussions to prioritise determinants and select implementation strategies. Employing a mixed methods convergent design, participants’ experiences of tailoring are evaluated using multiple data sources (observation notes, surveys, interviews). Findings are integrated using a triangulation protocol. Data are combined using joint displays for within and cross-case analysis.

**Results**

In total 8 DAFNE centres in Ireland comprising 40 clinicians have participated to date in the tailoring process. Teams prioritised determinants important to address now, including lack of available resources (administration support), access to knowledge and information (familiarity with course content), and networking and communication (long-standing relationships). A total of 27 clinicians from 7 centres have completed post-tailoring evaluation interviews to date. Findings from these interviews suggest the process is acceptable and feasible to clinicians, facilitating a dedicated opportunity to discuss DAFNE. However, additional guidance and evidence were not often used when prioritising determinants.

**Conclusion**

The findings will inform best-practices for developing tailoring approaches which are feasible and acceptable to clinical stakeholders, and which incorporate the guidance and evidence they use and value to make decisions during tailoring.

**Trial Registration:** Not applicable

**Consent to publish:** Not applicable

## P44: Supporting vulnerable families online – exploring the experiences of service users and practitioners and the development of a best practice framework to support the implementation of digital social care services

### Gráinne Hickey, Niamh McCarthy, Lauren Maguire, Siobhan Greene

#### Barnardos Ireland, Christchurch Square, Dublin 8, D08 DT63, Ireland

##### **Correspondence:** Gráinne Hickey (grainne.hickey@barnardos.ie)

*Implementation Science 2024*, **19(1)**:P44

**Background**

Digital technology is an increasing feature of social care practice, and its use has accelerated greatly in response to the Covid-19 pandemic. Yet there remains much that we need to learn regarding the implementation of digital interventions in social care settings. We explored service user and practitioner experiences of working online during the pandemic and outline the development of a digital practice framework. This work was conducted in the Republic of Ireland as part of an evaluation of web-based services delivered by Barnardos Ireland during the Covid-19 pandemic.

**Methods**

A mixed methods study combining survey and qualitative research was conducted. In total, 139 parent/adult service users and 102 practitioners took part in online surveys. Nineteen focus groups with 106 practitioners were also conducted. The findings informed the development of a best practice framework that includes guidance documents, protocols, and assessment tools to support staff and service users working online.

**Results**

Survey results indicated that more than half of participating parents identified a blended approach of online and face-to-face meetings as their preferred option for receiving services. Results from the survey and focus groups with practitioners indicted they generally felt confident and comfortable engaging in digital service delivery. Benefits of digital practice included perceived positive impacts on participation rates, ease of access and removal of barriers to engagement. Challenges included lack of access to technology/WiFi, inadequate spaces to engage in digital intervention, concerns regarding privacy and safeguarding and developmental considerations in direct work with children online.

**Conclusion**

The findings highlight benefits and challenges within the implementation of digital social care supports. The digital practice framework developed in response to these findings provides implementation guidance for web-based social care services including: session planning guidance; safeguarding and risk assessments; maintaining programme fidelity; building therapeutic relationships; and evaluation and reflection following intervention delivery.

**Trial Registration:** Not applicable

**Consent to publish:** Not applicable

## P45: Applying an implementation science lens to Ireland’s national care experience programme

### Lisa Ann Kennedy^1^, Conor Foley^2^, Tracy O’Carroll^1^, Rachel Flynn^2^

#### ^1^Health information and Quality Authority, George’s Court, George’s Lane, Dublin 7, D07 E98Y, Ireland; ^2^Health information and Quality Authority, Unit 1301, City Gate, Mahon, Cork, T12 Y2XT, Ireland

##### **Correspondence:** Lisa Ann Kennedy (lakennedy@hiqa.ie)

*Implementation Science 2024*, **19(1)**:P45

**Background**

Ireland’s National Care Experience Programme (NCEP) is a joint initiative from the regulator (Health Information and Quality Authority, HIQA), the provider (the Health Service Executive, HSE) and the policy-maker (the Department of Health). The Programme has conducted surveys of people’s experiences in Irish care settings since 2017, including experiences of inpatient, maternity, maternity bereavement, nursing home, and end of life care. Survey findings help to identify areas requiring improvement at local and national levels. Implementation science provides frameworks for understanding how the findings can be used to inform meaningful changes in care settings.

**Method**

Five of the dominant Implementation Science frameworks were adopted in the analysis: the Consolidated Framework for Implementation Research (CFIR)[1], Reach Effectiveness Adoption Implementation and Maintenance (RE-AIM)[2], Active Implementation Framework (AIF)[3], Normalisation Process Theory (NPT)[4], and Promoting Action on Research Implementation in Health Services (PARIHS)[5]. The aims, objectives and operationalisation of the NCEP were mapped to key elements of each framework using a framework-based approach with a view to understanding how each could contribute to the impact of the NCEP.

**Results**

The frameworks identified were potentially useful at the practice and policy levels, and could assist in identifying barriers and facilitators to implementing change, developing appropriate implementation strategies, and evaluating implementation success. The frameworks also provided insights into the design and development of the NCEP surveys themselves.

**Conclusion**

Insights from implementation science can aid the development and implementation of care experience surveys, as well as facilitating the utilisation of the survey findings to optimise their overall impact. An implementation science lens can support the translation of the findings of care experience surveys into meaningful improvements in the delivery of care.

**Trial Registration:** Not applicable

**Consent to publish:** Yes

**References**

1. Damschroder LJ, Reardon CM, Widerquist MA, Lowery J. The updated Consolidated Framework for Implementation Research based on user feedback. Implementation Science. 2022 Dec;17(1):1-6.
2. Glasgow RE, Vogt TM, Boles SM. Evaluating the public health impact of health promotion interventions: the RE-AIM framework. American journal of public health. 1999 Sep;89(9):1322-7
3. Fixsen, D. L., Naoom, S. F., Blase, K. A., Friedman, R. M. & Wallace, F. (2005). Implementation Research: A Synthesis of the Literature. Tampa, FL: University of South Florida, Louis de la Parte Florida Mental Health Institute, The National Implementation Research Network (FMHI Publication #231).
4. May CR, Mair F, Finch T, MacFarlane A, Dowrick C, Treweek S, Rapley T, Ballini L, Ong BN, Rogers A, Murray E. Development of a theory of implementation and integration: Normalization Process Theory. Implementation Science. 2009 Dec;4(1):1-9.
5. Rycroft-Malone J. The PARIHS framework—a framework for guiding the implementation of evidence-based practice. Journal of nursing care quality. 2004 Oct 1;19(4):297-304.

## P46: Using Geographic Information System (GIS) to understand service user’s patterns of accessing primary healthcare facilities in Goa, India

### Luanna Fernandes^1^, Bijayalaxmi Biswal^1^, Shanu Usgaokar^1^, Yashi Gandhi^1^, Urvita Bhatia^1^, Abhijit Nadkarni^1,2^, Chris Grundy^3^

#### ^1^Addictions and related-Research Group, Sangath, Goa, India; ^2^Department of Population Health, London School of Hygiene and Tropical Medicine, London, UK; ^3^Department of Social and Environmental Health Research, London School of Hygiene and Tropical Medicine, London, UK

##### **Correspondence:** Luanna Fernandes (luanna.fernandes@sangath.in)

*Implementation Science 2024*, **19(1)**:P46

**Background**

Geographic Information Systems (GIS) have been used for planning and monitoring implementation programs [1,2,3]. However, this is not the case for implementation planning in India [4,5]. Goa is the smallest state in India, with a high-density public health system with supposed catchment areas based on administrative boundaries. The aim of the current GIS mapping exercise was to define designated catchment areas for 30 public healthcare facilities in the state of Goa, India for a cluster hybrid type 2 implementation-effectiveness Randomised Cluster Trial (RCT) on depression care and understand the patterns of service use.

**Methods**

A base map of Goa was uploaded on the QGIS creating a map of administrative boundaries. Coordinate data for 30 healthcare facilities in Goa was collected using Google Maps. Two methods were used to identify service usage patterns: one retrospective through facility registers and second, self-reported data for a week to fill gaps in poorly maintained facility registers.

**Results**

Of the 425 villages, people from only 17.41% of villages attended just one health facility. The overlap of people from a given village visiting multiple facilities was large, the rationales for these were mainly access to public transport and better services. There was also differential usage based on gender with women preferring to visit facilities closer to their maternal homes. Geographical proximity and administrative boundaries do not appear to play the largest role in service usage.

**Conclusion**

Through the process, we defined 28 catchment areas for the cluster RCT. Using GIS mapping to define accurate catchment areas of healthcare facilities is an essential step in designing and implementing health system-wide programs as this provides a comprehensive and visual representation of access to care. Other programs should also consider understanding the same as part of their program set-up procedures.

**Trial Registration:** Not applicable

**Consent to publish:** Not applicable

**References**

1. Mahler H, Searle S, Plotkin M, Kulindwa Y, Greenberg S, Mlanga E, et al. Covering the last kilometer: Using GIS to scale-up voluntary medical male circumcision services in Iringa and Njombe Regions, Tanzania. Glob Health Sci Pract. 2015;3:503–15.
2. Noor AM, Alegana VA, Gething PW, Snow RW. A Spatial National Health Facility Database for Public Health Sector Planning in Kenya in 2008. Int J Health Geogr. 2009;8:13.
3. Robin TA, Khan MA, Kabir N, Rahaman ST, Karim A, Mannan II, et al. Using spatial analysis and GIS to improve planning and resource allocation in a rural district of Bangladesh. BMJ Glob Health. 2019;4(Suppl 5).
4. Sharma AK, Ruiz MOH. Application of GIS in Public Health in India: A literature-based review, analysis, and recommendations. Indian Journal of Public Health. 2016;60:51.
5. Sharma AK. Role of GIS in Health Management Information System and Medical Plan: A case study of gangtok area, Sikkim, India. International Journal of Environment and Geoinformatics. 2015;2:16–24.

## O47: Tailored strategies to address determinants of practice: a systematic review protocol

### Sheena M. McHugh^1^, Fiona Riordan^1^, Jane Murphy^1^, Laura-Jane McCarthy^1^, Claire Kerins^2^, Eimear Morrissey^3^, Danielle Adams^4^, Siobhan O’Connor^5^, Éilis J. O’Reilly^1^, Rosemary Meza^6^, Cara C. Lewis^7^, Byron J. Powell^4,8,9^, Michel Wensing^10^, Signe Flottorp^11^, Luke Wolfenden^12^

#### ^1^School of Public Health, University College Cork, Cork, Ireland; ^2^Health Promotion Research Centre, University of Galway, Galway, Ireland; ^3^School of Psychology & School of Medicine, University of Galway, Galway, Ireland; ^4^Center for Mental Health Services Research, Brown School of Social Work and Public Health, Washington University in St. Louis, St Louis, USA; ^5^Division of Nursing, Midwifery and Social Work, School of Health Sciences, The University of Manchester, Manchester, UK; ^6^Kaiser Permanente Washington Health Research Institute, Seattle, Washington, USA; ^7^NHLBI's Center for Translation Research and Implementation Science (CTRIS), Maryland, USA; ^8^Division of Infectious Diseases, John T. Milliken Department of Medicine, Washington University School of Medicine, Washington University in St. Louis, St Louis, USA; ^9^Center for Dissemination and Implementation, Institute for Public Health, Washington University in St. Louis, St Louis, USA; ^10^Dept. of General Practice and Health Services Research, University Hospital Heidelberg, Germany; ^11^Norwegian Institute of Public Health, Oslo, Norway; ^12^School of Medicine and Public Health, College of Health, Medicine, and Wellbeing, the University of Newcastle, Newcastle, UK

##### **Correspondence:** Fiona Riordan (fiona.riordan@ucc.ie)

*Implementation Science 2024*, **19(1)**:O47

**Background**

Tailoring has generally been described as a prospective process for selecting and modifying strategies to address contextual determinants of implementation to increase implementation success. A Cochrane review (2015) reported a small to moderate effect of a tailored strategy compared to no strategy or a non-tailored strategy, concluding that methods of tailoring are not yet well developed or described in published studies. Since 2015, several new studies of tailored strategies have been published. Therefore, we aim to update this review to determine whether tailored strategies are effective in improving professional practice and healthcare outcomes.

**Methods**

We conducted searches of The Cochrane Library, MEDLINE, EMBASE, PubMed, CINAHL, and the British Nursing Index, two grey literature databases, and three trial registers. Studies were eligible for inclusion if they were randomised controlled trials of tailored strategies which reported either professional practice or patient healthcare outcomes and where at least one group received a tailored strategy. Title/abstract and full texts were screened independently in Covidence by two authors. Two authors will independently assess quality and extract data.

**Results**

Overall, 6772 papers were identified from database searches and 2479 from trial registers. Full text screening (*n*=788) is underway. For each comparison for each outcome, where feasible we aim to conduct a pooled quantitative synthesis, otherwise we will present a narrative synthesis approach in line with Synthesis Without Meta-analysis guidance. We will conduct the following subgroup analyses: sample size; study setting (high/middle/low-income countries); use of theory, evidence, and stakeholders in the tailoring process.

**Conclusion**

Since the last revision of this review several new studies of tailored strategies have been published partly owing to the legitimization of the field with the flagship journal, Implementation Science (2006), and subsequent field-specific journals. This review update will identify additional evidence on the effectiveness of tailoring or on how it can be undertaken most effectively.

**Trial Registration:** Not applicable

**Consent to publish:** Not applicable

## O48: Implementation-minded policy making: an evidence synthesis

### Jane Lewis^1^, Anne-Marie Baan^1^, Emma Wills^1^, Amy Lloyd^2^, Dan Bristow^2^

#### ^1^Centre for Evidence and Implementation, London, England, UK; ^2^Wales Centre for Public Policy, Cardiff, Wales, UK

##### **Correspondence:** Jane Lewis (Jane.lewis@ceiglobal.org)

*Implementation Science 2024*, **19(1)**:O48

**Background**

Across policy fields, there is recurrent evidence that policies often fail to achieve their objectives, explained in part by implementation challenges. Features of government-led policy raise particular challenges, including that such policy is developed by individuals and groups distant from implementing settings; mandated or regulated; intended to be applied widely; and driven in part by political interests that may not reflect sectoral interests. Our study analyses the features of policy implementation that are associated with success and failure, looking across policy fields. We synthesise policy implementation barriers and facilitators, and the strategies used or recommended to address them in policy development and policy implementation.

**Methods**

We identified policy resources (e.g. guides and toolkits) that make recommendations for policy implementation. Through a systematic organisational website search, we identified and screened 113 resources and selected 10. We searched seven databases for systematic and other reviews of studies and evaluations of policy implementation and that identify associated barriers, facilitators and strategies. We screened 4043 potentially relevant texts, identified 50 as eligible, and prioritised 15 for inclusion. These covered a range of policy domains, forms of government and implementation settings.

**Results**

The degree to which policies are aligned with their implementation contexts (e.g. social, institutional, political) creates potential barriers and facilitators which can be addressed in policy development or delivery or both, with mutually reinforcing and compensating mechanisms at play. Successful implementation requires justified and clear policy objectives, selection of comprehensive and tailored change strategies, stakeholder engagement, leadership, implementation planning, resource allocation, and monitoring and evaluation.

**Conclusion**

Policy effectiveness calls for approaches that embed implementation thinking in policy development, rather than viewing implementation as a discrete phase of policy execution or delivery.

**Trial Registration:** Not applicable

**Consent to publish:** Yes

## P49: Implementing a new ‘Test, Evidence, Transition’ programme to accelerate the effective and equitable adoption of cancer pathway innovations

### Luke Weaver^1^, Alexandra Feast^1^, Brian Knowles^1^, Natalie Masento^1^, Naser Turabi^2^, Kate Hamilton-West^1^

#### ^1^Social and Behavioural Research, Cancer Research UK, London, E20 1JQ, United Kingdom; ^2^Evidence & Implementation, Cancer Research UK, London, E20 1JQ, United Kingdom

##### **Correspondence:** Luke Weaver (luke.weaver@cancer.org.uk)

*Implementation Science 2024*, **19(1)**:P49

**Background**

Cancer Research UK has launched a new programme of commissioned activity, ‘Test Evidence Transition’, which aims to accelerate the effective adoption of innovations whilst reducing inequalities. The objective is to drive the transition of effective interventions from innovation into mainstream NHS practice, addressing the ‘implementation gap’ to improve the experience and outcomes of those affected by cancer.

**Methods**

The programme closely supports frontline NHS teams working to achieve three objectives: *Test* innovations to support optimal cancer pathways that transform clinical practice; *Evidence* the process, outcome, and impact of implementation; and work with strategic partners to ensure the *Transition* of evidence-based approaches into effective and equitable adoption across the NHS.

The programme combines top-down ‘push’ approaches (system levers) with bottom-up ‘pull’ approaches (real-time learning and collaboration) to stimulate sustained pathway improvements. As an active commissioner, we provide strategic oversight, creating a community of stakeholder expertise, including academic and clinical partners who will co-design resources, informed by relevant clinical science frameworks, to support implementation, evaluation and scalability.

**Results**

We present emerging findings and insights from the first phase of the programme, commenced in April 2023 and concluding in November 2024, providing funding and support to three frontline NHS teams exploring pathway innovations for cancer. Evaluation plans cover clinical impact, acceptability and cost effectiveness, including analysis of health economic and patient reported outcomes. Projects will report on programme inputs, outputs, outcomes, and factors influencing implementation, sustainability, scalability and evaluability.

**Conclusion**

In delivering a focused model to pioneer health system transformational change, the programme provides a test bed for innovations that transform clinical practice and optimise the cancer pathway, triangulating and interpreting evidence and evaluation to enable acceleration into mainstream practice. The programme will provide high-quality evidence to decision-makers on how best to address the challenges of translation, aiding the implementation and spread of identified best practice.

**Trial Registration:** Not applicable

**Consent to publish:** Not applicable

## P50: What is needed to implement self-management support (SMS) in cancer care delivery? A qualitative study of SMS implementation in Ireland

### Nickola Pallin^1^, Sheena McHugh^1^, Roisin Connolly^2^, Josephine Hegarty^3^, John Browne^1^

#### ^1^School of Public Health, University College Cork, Cork, Ireland; ^2^Cancer Research @UCC, College of Medicine and Health, University College Cork, Cork, Ireland; ^3^School of Nursing and Midwifery, University College Cork, Cork, Ireland

##### **Correspondence:** Nickola Pallin (npallin@ucc.ie)

*Implementation Science 2024*, **19(1)**:P50

**Background**

Self-management support (SMS) is a key component of quality cancer survivorship delivery [1]. However, implementation is a problem internationally. In response to national policy an evidence-based SMS programme ‘Cancer Thriving and Surviving’ (CTS) has been rolled out in Ireland [2]. However, implementation is not uniform across contexts. We report the main barriers, facilitators, and contextual factors relevant to implementing SMS across cancer organisations in Ireland.

**Methods**

The Consolidated Framework for Implementation Research (CFIR) informed the topic guide and analysis [3]. Transcripts were analysed inductively by the interview guide and the research questions. Categories were then coded deductively to the CFIR constructs. Analysis of the interviews were further sensitised by Normalisation Process Theory to help uncover how and why CTS is implemented [4].

**Results**

Interviews were conducted with 47 stakeholders (nurses, physiotherapists, occupational therapists, dietitians, oncologists, psychologists, psychiatrists, social workers, and programme deliverers living with and beyond cancer) from 19 organisations. Findings highlight that when stakeholders believe in the benefit of CTS on patient outcomes and when these outcomes are aligned with personal and organisational goals implementation becomes a priority. When aligned with organisational goals leadership had stronger buy-in and secured resources to enable implementation. The need for policy support; regulatory and professional guidelines highlighting CTS for implementation may secure buy in. Enablers included a positive organisational culture of deliverer-centeredness, with performance feedback and incentives. As well as collaboration among stakeholders, characterised by close working relationships and communication processes across and within organisations.

**Conclusion**

These findings highlight theoretically based factors that influence implementation of SMS, which can be used to inform tailoring of implementation strategies. Strategies that improve awareness regarding the positive impact of SMS, align SMS with organisational goals, secure buy-in and support a culture of delivered centeredness and collaboration may be needed to implement SMS.

**Trial Registration:** Not applicable

**Consent to publish:** yes

**References**

1. Howell D, Mayer DK, Fielding R, Eicher M, Verdonck-de Leeuw IM, Johansen C, Soto-Perez-de-Celis E, Foster C, Chan R, Alfano CM, Hudson SV. Management of cancer and health after the clinic visit: a call to action for self-management in cancer care. Jnci: journal of the national cancer institute. 2021;113(5):523-31.
2. Gibbons M, Love D, Hanan T, Mullen L. Implementing Cancer Thriving and Surviving. A Stanford Model Self-Management Programme. 2020. Dublin.
3. Damschroder LJ, Reardon CM, Widerquist MA, Lowery J. The updated Consolidated Framework for Implementation Research based on user feedback. Implementation Science. 2022;17(1):1-6.
4. May C, Finch T. Implementation, embedding, and integration: an outline of Normalization Process Theory. Sociology. 2009.43(3):535–54.

## P51: Exploring outcomes from a novel shared professional training and wrap around support package on sustaining complex intervention fidelity: lessons from LISTEN

### Fiona J. Leggat¹, Nick Sevdalis², Fiona Jones¹^,^³

#### ¹Population Health Research Institute, St George’s, University of London, London, England, UK; ² Centre for Behavioural and Implementation Science Interventions, Yong Loo Lin School of Medicine, National University of Singapore, Singapore; ³ Centre for Applied Health and Social Care Research, Faculty of Health, Social Care and Education, Kingston University, London, England, UK

##### **Correspondence:** Fiona J. Leggat (fleggat@sgul.ac.uk)

*Implementation Science 2024*, **19(1)**:P51

**Background**

Healthcare professionals (HPs) play primary roles in delivering complex rehabilitation interventions. However, when delivering a complex intervention within a trial context, additional training and support is critical to enhance HP’s ability to ensure fidelity and deliver as intended. Intervention delivery is recognised within implementation science frameworks, yet the appropriateness and influence of training on fidelity and adherence to core principles is often not evaluated. Developed for a clinical trial to co-design and evaluate self-management support for people with Long Covid (LISTEN), we report on the design and impact of a novel training and support package on HPs’ knowledge, confidence, and skills.

**Methods**

Underpinned by the Consolidated Framework for Implementation Research, the co-designed LISTEN training was formatively evaluated using an online self-report survey. All HPs (e.g., physiotherapists [PTs], occupational therapists [OTs], psychologists, nurses, and other practitioners) who undertook the 8-hour interactive group-based training took part in the survey. The survey asked HPs to separately score knowledge and confidence across 9 intervention fidelity criteria (37 items). 3-point Likert scales (1-3) were used for each item. Impacts from the training and support package will be assessed using focus groups and fidelity observations. Data were analysed using descriptive statistics.

**Results**

57 HPs completed the survey and subsequently delivered intervention sessions. Average knowledge (94%) and confidence (90%) varied across skills and between professions. Psychologists’ self-assessment of overall knowledge (97%) and confidence (92%) post-training was higher than those of OTs (94%, 90%) and nurses (93%, 91%). PTs reported lowest levels of knowledge (88%) and confidence (81%), although for some skills (e.g., attentive listening, curiosity) scores mirrored other professions.

**Conclusion(s)**

HPs participating in shared professional training to deliver complex interventions require tailored support to address profession specific needs. The influence of the support package on intervention fidelity continues to be evaluated within the ongoing LISTEN trial.

**Trial Registration:** LISTEN Trial (ISCTRN 36407216)

**Consent to publish:** Not applicable

## O52: Evaluation of implementation of the GLA:D® Ireland programme for hip and knee osteoarthritis across public and private healthcare settings in the first year

### Clodagh M. Toomey^1,2,3^, Avantika Bhardwaj^1,2^, Norelee Kennedy^1,2^, Anne MacFarlane^3,4^

#### ^1^School of Allied Health, University of Limerick, Limerick, Ireland V94 T9PX; ^2^Health Research Institute, University of Limerick, Limerick, Ireland V94 T9PX; ^3^Public and Patient Involvement Research Unit, University of Limerick, Limerick, Ireland V94 T9PX; ^4^School of Medicine, University of Limerick, Limerick, Ireland V94 T9PX

##### **Correspondence:** Clodagh M. Toomey (Clodagh.toomey@ul.ie)

*Implementation Science 2024*, **19(1)**:O52

**Background**

The Good Life with osteoArthritis Denmark (GLA:D®) non-profit initiative is a bottom-up approach to deliver evidence-based care, including exercise and education, to people with hip or knee osteoarthritis. GLA:D® Ireland commenced in October 2021, using a participatory approach to co-design implementation strategies that would ensure optimal and equitable access to the evidence-based programme. The objective is to determine the adoption, acceptability, appropriateness, feasibility and penetration of GLA:D® Ireland across different healthcare settings in Ireland, in the first year of implementation.

**Methods**

Quantitative implementation outcomes collected from the GLA:D® Ireland Registry were analysed from November 2021–2022. Physiotherapists who completed a two-day training course were asked to register patients with osteoarthritis who underwent the intervention, using REDCap™ electronic data capture form. Patients were subsequently sent questionnaires to complete. Table 1 lists implementation evaluation methods and results.

**Results**

In the first year, 71 physiotherapists attended one of three GLA:D® training courses (41% primary care, 38% public hospital, 21% private practice). Of 130 patients screened, 41% were from the three sites with more than one physiotherapist trained (one primary care (*n*=2) and two public hospital sites (*n*=4 each)).

**Conclusions**

While GLA:D® was found to be acceptable, appropriate and feasible, and was adopted by many primary care settings in the first year, penetration was more successful in acute hospital settings, with more resources, physiotherapists trained and consultant referrals. A greater understanding of enablers to implementation in primary care settings may help to ensure timely and equitable access to the programme across Ireland.

**Trial Registration:** Not applicable

**Consent to publish:** Not applicable

**Table 1 (Abstract O52):** Evaluation of implementation outcomes of the GLA:D® Ireland programme

Implementation Outcome	Method and Result
Adoption	23/71 (32%) of trained physiotherapists implemented at least one programme across 15 distinct sites (*n*=10 primary care, *n*=2 public hospital, *n*=3 private practice) across all four provinces in Ireland (*n*=6 Munster, *n*=4 Connacht, *n*=3 Leinster, *n*=2 Ulster).
Acceptability	*Acceptability of intervention measure (AIM)* – completed by physiotherapists following training, with maximum score of five. Mean 4.7 (Standard Deviation (SD) 0.5), range 4-5
Appropriateness	*Intervention appropriateness measure (IAM)* – completed by physiotherapists following training, with maximum score of five. Mean 4.5 (SD 0.5), range 3-5
Feasibility	*Feasibility of intervention measure (FIM)* – completed by physiotherapists following training, with maximum score of five. Mean 4.1 (SD 0.6), range 3-5
Penetration	No. patients screened *n*=130 (47% primary care, 35% public hospital, 18% private practice) No. patients refused consent to share data *n*=2 No. patients completed baseline questionnaires *n*=94 (72%) No. patients completed follow-up questionnaires *n*=51 (39%) Referral sources – GP referral (*n*=50) Advanced practice physiotherapist referral (*n*=23), Orthopaedic consultant referral (*n*=22) Other healthcare professional (*n*=20) Clinic waitlist (*n*=12), Patient self-referral (*n*=3)

## O53: A process evaluation of a quality improvement collaborative (QIC) to improve the uptake of insulin pumps for people with type 1 diabetes (EQUIPD Study)

### Melissa Girling, Elaine O’ Halloran, Michael Sykes, Tracy Finch (on behalf of the EQUIPD team)

#### Department of Nursing, Midwifery & Health, Northumbria University, Newcastle upon Tyne, UK

##### **Correspondence:** Melissa Girling (melissa.girling@northumbria.ac.uk)

*Implementation Science 2024*, **19(1)**:O53

**Background**

People with type 1 diabetes and raised blood sugar levels are at greater risk of health complications [1]. NICE recommends continuous subcutaneous 'insulin pump' therapy for people with type 1 diabetes and high blood sugar levels [2]. The National Diabetes Audit (NDA) has identified over 90,000 who meet these criteria but who are not using an insulin pump. Increasing the capabilities of healthcare providers to respond to feedback from national audits may improve care. The EQUIPD study is an efficient cluster randomised trial of a quality improvement collaborative (QIC) aligned to the National Diabetes Audit that seeks to enhance the improvement capabilities of feedback recipients to increase the uptake of insulin pumps in line with NICE guidance.

**Materials and methods**

Over a trial period of 34 months, we are undertaking a process evaluation to understand intervention implementation, engagement, fidelity and tailoring of actions. The evaluation includes observations of QIC virtual workshops, theory-informed interviews with intervention participants, and documentary analysis (e.g., Jamboards). The analytic process will draw upon: organisational readiness to change theory [3] to describe the target behaviours undertaken by intervention recipients; normalisation process theory (NPT) [4] to explore how teams implement the target behaviours and, behaviour change techniques (BCTs) to describe delivery [5].

**Results**

The process evaluation is ongoing. Initial stages have focused on coding behaviour change techniques within the intervention materials and conducting fidelity assessment of their delivery within virtual workshops. Next steps will include analysis of semi-structured interviews with intervention participants.

**Conclusions**

The process evaluation alongside an effectiveness trial provides an opportunity to describe how implementers engage with the QIC intervention overall to support improvement activity and how context influences this work (implementation and engagement); assess fidelity of delivery, receipt, and enactment of the QIC intervention (fidelity) and describe how teams enact tailoring (tailoring).

**Consent to publish:** Not applicable

**Trial registration**: ISCRTN82176651

**References**

1. National Diabetes Audit, 2019-20, Type 1 Diabetes. NHS Digital, Leeds. 2021. Available from: https://digital.nhs.uk/data-and-information/publications/statistical/national-diabetes-audit/national-diabetes-audit-2019-20-type-1-diabetes
2. National Institute for health and Care Excellence. Continuous subcutaneous insulin infusion for the treatment of diabetes mellitus Technology appraisal guidance [TA151]; 2008. Available from: https://www.nice.org.uk/guidance/ta151
3. Weiner BJ. A theory of organizational readiness for change. Implementation science. 2009 Dec;4(1):1-9.
4. May C, Finch T. Implementing, embedding, and integrating practices: an outline of normalization process theory. Sociology. 2009 Jun;43(3):535-54.
5. Michie S, Richardson M, Johnston M, Abraham C, Francis J, Hardeman W, et al. The behaviour change technique taxonomy (v1) of 93 hierarchically clustered techniques: Building an international consensus for the reporting of behaviour change interventions. Annals of Behavioural Medicine. 2013 Aug;46(1):81-95.

## O54: Using logic models to advance the implementation of complex genomics sequencing within a complex care pathway

### Joseph Elias^1^, Rona Weerasuriya^2,3^, Melissa Martyn^3,4,5^, Sophie O’Haire^6^, Kortnye Smith^6^, Clara Gaff^3,4,5^, Natalie Taylor^1^

#### ^1^School of Population Health, UNSW, Sydney, NSW, Australia; ^2^The Burnet Institute, Melbourne, Victoria, Australia; ^3^Murdoch Children’s Research Institute, Melbourne, Victoria, Australia; ^4^Melbourne Genomics Health Alliance, Melbourne, Victoria, Australia; ^5^Department of Paediatrics, University of Melbourne, Victoria, Australia; ^6^Peter MacCallum Cancer Centre, Melbourne, Victoria, Australia

##### **Correspondence:** Joseph Elias (Joseph.Elias1@unsw.edu.au)

*Implementation Science 2024*, **19(1)**:O54

**Background**

Advances in complex genomic sequencing (CGS) raise the possibility of personalised care for advanced cancer patients [1]. However, oncologists report many challenges to use of CGS, particularly outside academic centres of excellence [2]. Implementation science methods can inform the design of service interventions to improve the incorporation of CGS within care pathways. Our study aimed to develop an Implementation Research Logic Model (IRLM) to represent the optimal pathways for CGS implementation [3].

**Methods**

*Phase 1:* Interviewed oncologists (*n*=11) who delivered CGS to advanced cancer patients. Barriers were coded to the CFIR, and implementation strategies were matched using the CFIR/ERIC tool [4,5]. Three service model interventions emerged through intuitive coding (centralised experts, local superusers, and point of care resources), and were well-aligned with ERIC strategies. *Phase 2:* Conducted virtual focus groups with oncologists (*n*=10), facilitated by an online quantitative data collection tool, to gather preferences for the operationalisation of each service model. CFIR/ERIC was used to generate a suite of service model-specific implementation strategies. Data collected across both phases was inputted into an IRLM.

**Results**

The IRLM represents a number of hypothesised relationships between implementation factors for each service intervention. For example, the IRLM describes the local superuser (LSU) *(ERIC: identify/prepare champions)* as a service intervention that can address oncologists’ low confidence to discuss germline findings during patient consenting *(CFIR: self-efficacy)*. The IRLM also represents operational challenges such as difficulties recruiting superusers at regional/rural sites *(CFIR: available resources)* and proposes identifying site-specific barriers/facilitators *(ERIC: assess for readiness)* to enable sites to train appropriate LSU’s and plan for continuity/redundancy *(hypothesised mechanism)*.

**Conclusions**

IRLMs offer a framework for describing causal pathways and complex relationships between implementation determinants, interventions, and outcomes. Ultimately, these assumed relationships can be theoretically or empirically evaluated to aid in the development of more effective implementation/service interventions.

**Trial Registration:** Not applicable

**Consent to publish:** Not applicable

**References**

1. O’Haire S, Degeling K, Franchini F, Tran B, Luen SJ, Gaff C, et al. Comparing Survival Outcomes for Advanced Cancer Patients Who Received Complex Genomic Profiling Using a Synthetic Control Arm. Target Oncol. 2022;17:539–48.
2. Taylor N, Best S, Martyn M, Long JC, North KN, Braithwaite J, et al. A transformative translational change programme to introduce genomics into healthcare: a complexity and implementation science study protocol. BMJ Open. British Medical Journal Publishing Group; 2019;9.
3. Smith JD, Li DH, Rafferty MR. The Implementation Research Logic Model: a method for planning, executing, reporting, and synthesizing implementation projects. Implement Sci. 2020;15:84.
4. Damschroder L. Fostering implementation of health services research findings into practice: a consolidated framework for advancing implementation science. Implement Sci IS. 2009;4:50.
5. Powell BJ, Waltz TJ, Chinman MJ, Damschroder LJ, Smith JL, Matthieu MM, et al. A refined compilation of implementation strategies: Results from the Expert Recommendations for Implementing Change (ERIC) project. Implement Sci. 2015;10:21.

## P55: Barriers and enablers to participating in self-management support among those living with and beyond cancer: a qualitative study informed by the TDF and COM-B

### Nickola Pallin^1^, Sheena McHugh^1^, Roisin Connolly^2^, John Browne^1^

#### ^1^School of Public Health, University College Cork, Cork, Ireland; ^2^Cancer Research @UCC, College of Medicine and Health, University College Cork, Cork, Ireland

##### **Correspondence:** Nickola Pallin (npallin@ucc.ie)

*Implementation Science 2024*, **19(1)**:P55

**Background**

National policy in Ireland has recommended that cancer services implement survivorship programmes which includes self-management with support. Self-management support (SMS) programmes have been adopted but implemented with varying levels of reach (uptake) among people living with and beyond cancer (LWBC) [1,2]. This study aimed to identify the enablers and barriers to participating in SMS among those LWBC in Ireland to inform tailoring of implementation strategies to increase reach of SMS.

**Methods**

This is a qualitative study. Semi-structured interviews were conducted with people LWBC. The Theoretical Domains Framework (TDF) informed the topic guide and analysis [3]. Inductive thematic analysis was conducted to identify categories relating to barriers and enablers to participating in SMS. These were then deductively mapped onto the TDF and capability, opportunity, motivation and behaviour (COM-B) model [3,4].

**Results**

Twenty-eight interviews were conducted. Eleven had taken part in a SMS programme. Emotion and identity (illness perception) shaped participants’ beliefs about whether they would choose to participate in a programme. A lack of knowledge of available programmes and how to access were commonly reported barriers, with participants describing limited information received from their healthcare providers. Inaccessible programmes due to timing and place of delivery (environmental context and resources) was also a common barrier. Social influences which include healthcare professionals and peer support groups were identified as key enablers. As well as supportive reminders (memory, attention, and decision-making).

**Conclusion**

We identified key factors that influence the capability, opportunity and motivation among those LWBC to participate in SMS. Findings suggest implementation strategies aiming to improve reach of SMS could target knowledge, memory, identity, emotion, environmental context and social influences. The results will be used to inform tailoring of implementation strategies to increase reach of a SMS programme currently adopted in Ireland for people LWBC.

**Trial Registration:** Not applicable

**Consent to publish:** yes

**References**

1. Gibbons M, Love D, Hanan T, Mullen L. Implementing Cancer Thriving and Surviving. A Stanford Model Self-Management Programme. 2020. Dublin.
2. van Der Hout A, van Uden-Kraan CF, Holtmaat K, Jansen F, Lissenberg-Witte BI, Nieuwenhuijzen GA, Hardillo JA, De Jong RJ, Tiren-Verbeet NL, Sommeijer DW, De Heer K. Role of eHealth application Oncokompas in supporting self-management of symptoms and health-related quality of life in cancer survivors: a randomised, controlled trial. The Lancet Oncology. 2020;21(1):80-94.
3. Cane J, O’Connor D, Michie S. Validation of the theoretical domains framework for use in behaviour change and implementation research. Implement Science. 2012;7: 37.
4. Michie S, van Stralen MM, West R. The behaviour change wheel: a new method for characterising and designing behaviour change interventions. Implement Science. 2011;6:42.

## O56: Implementation of a national programme for people presenting to emergency departments with self-harm and suicide-related ideation: a qualitative study of implementation determinants

### Selena O’Connell^1,2^, Grace Cully,^1,2^, Sheena McHugh^1^, Margaret Maxwell^3^, Anne Jeffers^4^, Katerina Kavalidou^2,4^, Sally Lovejoy^4^, Eve Griffin^1,2^

#### ^1^School of Public Health, University College Cork, Cork, Ireland; ^2^National Suicide Research Foundation, Cork, Ireland; ^3^NMAHP Research Unit, University of Stirling, Stirling, UK; ^4^Health Service Executive, Dublin, Ireland

##### **Correspondence:** Selena O’Connell (Selena.oconnell@ucc.ie)

*Implementation Science 2024*, **19(1)**:O56

**Background**

A national clinical programme (NCP) was first introduced in Ireland in 2014 to standardise the assessment, care planning and follow-up of people presenting to the emergency department (ED) with self-harm or suicidal ideation. This study aimed to explore the process and determinants of implementation of the NCP.

**Method**

The Consolidated Framework for Implementation Research (CFIR) [1,2] and documentary analysis were used to inform the interview topic guide. Semi-structured interviews (*n*=30) were conducted with staff involved in delivering the programme, primarily Clinical Nurse Specialists, Consultant Leads, Nursing Management and Emergency Medicine representatives. Participants were asked about the factors affecting implementation in early years (approx. 2015-2017) and in later years (2019-2022). Thematic analysis was used with primarily deductive coding based on CFIR and additional codes developed inductively. A second researcher independently coded 20% of transcripts. Findings were reviewed by the research team and are in the process of being finalised following review and feedback by NCP staff.

**Results**

All five CFIR domains were influential. Prominent factors were the perceived relative advantage of the NCP and clarity of key pillars of the programme as delivered in ED (innovation); links with community and primary care providers, financing and national-level governance (outer setting); relationships between members of the implementation team, availability of resources and work infrastructure within the ED (including out-of-hours cover) (inner setting); and processes of recording data and feeding back to sites (implementation process).

**Conclusion**

This study highlights the range of factors influencing a programme rolled out at a national level across ED’s. The context of existing services within hospitals strongly influenced the process of implementing the programme. Strategies that facilitated implementation included audit and feedback, promoting networking between sites, as well as supporting staff through regular meetings, training and career progression.

**Trial Registration:** Not applicable

**Consent to publish:** Not applicable

**References**

1. Damschroder LJ, Aron DC, Keith RE, Kirsh SR, Alexander JA, Lowery JC. Fostering implementation of health services research findings into practice: a consolidated framework for advancing implementation science. Implement Sci. 2009 Aug 7;4(1):50.
2. Damschroder LJ, Reardon CM, Widerquist MAO, Lowery J. The updated Consolidated Framework for Implementation Research based on user feedback. Implement Sci. 2022 Oct 29;17(1):75.

## O57: The evaluation and adaptation of the KwaZulu-Natal Auditory Implant Programme(KZN-AIP), South Africa

### Zandile Shezi^1^, Andile L. Sibiya^2^, Ayanda Gina^3^

#### ^1^Discipline of Audiology, School of Health Sciences, College of Health Sciences, University of KwaZulu-Natal, Durban, South Africa; ^2^Discipline of Otorhinolaryngology, School of Clinical Medicine, College of Health Sciences, University of KwaZulu-Natal, Durban, South Africa; ^3^Discipline of Audiology, School of Health Sciences, College of Health Sciences, University of KwaZulu-Natal, Durban, South Africa

##### **Correspondence:** Zandile Shezi (blosez@ukzn.ac.za)

*Implementation Science 2024*, **19(1)**:O57

**Background**

The KwaZulu-Natal Auditory Implant Programme (KZN-AIP) was launched in 2021, and an ongoing evaluation is recognized to ensure programme success. Considering the infancy of the KZN-AIP in providing a specialized service within the public sector as well as adopting a newly designed model not previously used within the health sector across South Africa, the effectiveness of this programme is unknown. This study aims to use the re-aim extension programme to evaluate and adapt an auditory implant programme in KZN, South Africa.

**Methods**

Post the launch of the KZN-AIP, the following dimensions were considered: Reach: considers the programmes promotion efforts, and number of referrals to measure growth. Effectiveness: considers the number of approved patients and patients implanted successfully, measurement of setting level and staff level. Implementation and Maintenance of the programme.

**Results**

Reach: in promoting the study, a launch of the programme that included 87 participants was facilitated. Detailed radio interviews and meetings with various stakeholders within the Department of Health, University of KwaZulu-Natal and private sector were conducted. In 2021 and 2022, twenty-six and eighteen referrals were received respectively from 9 different districts of KwaZulu-Natal. Effectiveness: To date, a total of 23 patients have been approved for implantation and a total of 11 patients (3 children, 8 adults) have been implanted. A significant growth is observed with staff development, as the programme began with 1 surgeon and 1 audiologist and currently has 3 surgeons, 3 audiologists and 1 speech therapist. Implementation: Includes referrals of candidates into the programme, assessments, regular candidacy discussions and management. Maintenance: This is an ongoing process that is inclusive of an internal and external programme audit.

**Conclusion**

The re-aim framework has provided the structure to systematically plan, implement, maintain and evaluate the KZNAIP while effectively progressing and with funding cited as the biggest challenge.

**Trial Registration:** Not applicable

**Consent to publish:** Not applicable

## P58: Perceived sustainability of a community paramedicine model to reduce acute care utilization: a case study in two settings

### Jennifer L. Ridgeway^1^, Olivia A. Smith^1^, Michelle A. Lampman^1^, Terri Menser^1^, Rozalina G. McCoy^1,2,3^

#### ^1^Division of Health Care Delivery Research, Robert D. and Patricia E. Kern Center for the Science of Health Care Delivery, Mayo Clinic, Rochester, MN, 55905, USA; ^2^Division of Community Internal Medicine, Geriatrics, and Palliative Care, Department of Medicine, Mayo Clinic, Rochester, Minnesota, 55905, USA; ^3^Community Paramedic Service, Mayo Clinic Ambulance, Rochester, Minnesota, 55905, USA

##### **Correspondence:** Jennifer L. Ridgeway (ridgeway.jennifer@mayo.edu)

*Implementation Science 2024*, **19(1)**:P58

**Background**

Community paramedics (CPs) provide services to patients with intermediate acuity needs in community settings [1]. The Care Anywhere with Community Paramedics (CACP) program was evaluated in a pragmatic trial of CACP versus usual care on days alive outside the hospital or emergency department within 30 days [2]. This study reports key informant perspectives of program sustainability at trial completion.

**Materials and methods**

The trial was conducted in an academic medical center and affiliated rural health system (Midwest USA). A purposive sample of CPs, clinicians and other care team members, and administrators were invited to complete a survey, including items from the Clinical Sustainability Assessment Tool (CSAT) [3]. A subset of participants was also invited to complete an individual interview. Surveys were analyzed descriptively by group, and interview transcripts were analyzed using methods of applied thematic analysis.

**Results**

Between January and March 2023, 63 individuals completed surveys and 21 completed interviews. CSAT scores were highest in clinician and care team perspectives of outcomes and effectiveness and lowest in CP perspectives of organizational readiness and among CPs overall (Table 1). Interview findings highlighted that clinicians and other members of the care team viewed the program as addressing persistent acute care gaps, especially in rural communities, and as providing them with important information to manage care and keep patients stable at home. However, CP interviews suggested that perceived sustainability hinges on improved staffing and better criteria for referrals so that they are not a “catch all” resource for referral of very complex patients.

**Conclusions**

CP programs may provide critically needed options for patients with intermediate acuity needs, but sustainability will require a balance between filling gaps in the healthcare system and CP capacity to address them.

**Trial registration**

ClinicalTrials.gov NCT05232799. Registered on 10 February 2022.

**Consent to publish**

This study was approved by the Mayo Clinic Institutional Review Board; participants provided informed consent.

**References**

1. van Vuuren J, Thomas B, Agarwal G, MacDermott S, Kinsman L, O’Meara P, et al. Reshaping healthcare delivery for elderly patients: the role of community paramedicine; a systematic review. BMC Health Serv Res. 2021;21(1):1-5. doi: 10.1186/s12913-020-06037-0.
2. Ridgeway JL, Gerdes EO, Dodge A, Liedl CP, Juntunen MB, Sundt WJ, et al. Community paramedic hospital reduction and mitigation program: study protocol for a randomized pragmatic clinical trial. Trials. 2023;24(1):1-6. doi: 10.1186/s13063-022-07034-w.
3. Malone S, Prewitt K, Hackett R, Lin JC, McKay V, Walsh-Bailey C, et al. The Clinical Sustainability Assessment Tool: measuring organizational capacity to promote sustainability in healthcare. Implement Sci Commun. 2021;2(1): 77. doi: 10.1186/s43058-021-00181-2.

**Table 1 (Abstract P58):** Descriptive statistics of clinical sustainability sub-domains

Domain	Community paramedics (***n***=7)	Clinicians and members of the care team (***n***=50)	Administrators (***n***=6)	All respondents
	Mean (SD)	Mean (SD)	Mean (SD)	Mean (SD)
**Organizational Readiness**	**4.45 (1.15)**	**5.22 (0.96)**	**4.97 (1.04)**	**5.11 (0.99)**
*Organizational systems are in place to support the various needs of the CACP program*	3.25 (1.26)	5.05 (1.25)	5.17 (1.94)	4.91 (1.41)
*The CACP program fits in well with the culture of the team*	6.00 (0.82)	6.36 (0.84)	6.50 (0.55)	6.35 (0.81)
*The CACP program has feasible and sufficient resources (e.g., time, space, funding) to achieve its goals*	4.00 (1.63)	4.74 (1.51)	3.67 (1.21)	4.51 (1.50)
*The CACP program has adequate staff to achieve its goals*	4.50 (1.00)	4.59 (1.32)	3.67 (1.22)	4.45 (1.29)
*The CACP program is well integrated into the operations of the organization*	4.50 (2.65)	5.11 (1.49)	5.83 (0.98)	5.15 (1.49)
**Workflow Integration**	**5.30 (0.71)**	**5.70 (0.93)**	**5.52 (1.03)**	**5.66 (0.93)**
*The CACP program is built into the clinical workflow*	4.50 (1.00)	4.89 (1.49)	5.00 (1.41)	4.87 (1.42)
*The CACP program is easy for clinicians to use*	4.00 (0.82)	5.68 (1.33)	6.00 (1.23)	5.57 (1.36)
*The CACP program integrates well with established clinical practices*	5.25 (1.71)	5.92 (1.04)	5.67 (1.37)	5.83 (1.13)
*The CACP program aligns well with other clinical systems (e.g., electronic health record)*	5.25 (1.71)	6.11 (0.99)	5.67 (1.86)	5.98 (1.19)
*The CACP program is designed to be used consistently*	4.75 (1.71)	5.68 (1.27)	5.50 (0.84)	5.57 (1.26)
**Outcomes & Effectiveness**	**4.35 (1.10)**	**6.18 (0.88)**	**5.56 (1.25)**	**5.91 (1.09)**
*The practice has evidence of beneficial outcomes*	5.00 (0.00)	6.21 (1.08)	5.67 (1.75)	6.02 (1.19)
*The practice is associated with improvement in patient outcomes that are clinically meaningful*	4.75 (0.50)	6.27 (0.90)	6.50 (0.84)	6.17 (0.96)
*The practice is clearly linked to positive health or clinical outcomes*	4.00 (1.16)	6.11 (0.94)	6.33 (1.21)	5.96 (1.14)
*The practice is cost-effective*	4.00 (1.83)	6.00 (1.12)	5.00 (1.55)	5.67 (1.38)
*The practice has clear advantages over alternatives*	4.00 (2.31)	6.43 (0.73)	4.60 (1.14)	6.02 (1.27)
**Overall Mean by Group**	**4.30**	**5.64**	**5.42**	**5.49**

7-point scale: 1= To little or no extent to 7= To a very great extent. Red text indicates the score for that item/domain is less than the overall mean for all CSAT domains by group

## O59: Developing an intervention and implementation strategy to improve delivery of evidence based care for knee pain attributed to degenerative meniscal tears in the primary care setting

### Helen O’Leary^1^, Elaine Toomey^2^, Karen McCreesh^1^

#### ^1^School of Allied Health, University of Limerick, Limerick, Ireland; ^2^School of Psychology, National University of Ireland, Galway, Ireland

##### **Correspondence:** Helen O’Leary (helen.oleary@ul.ie)

*Implementation Science 2024*, **19(1)**:O59

**Background**

Non-surgical approaches such as exercise therapy are recommended as first-line therapy for a degenerative meniscal tear (DMT)[1]; a common knee pain presentation in Irish orthopaedic clinics [2]. Despite strong recommendations against surgery, arthroscopy remains a common orthopaedic procedure for DMTs [3,4]. We aimed to develop an intervention and implementation strategy to improve non-surgical management of DMTs in the primary care setting that would target both health care practitioners (HCPs) and patient barriers to evidence based care.

**Materials and methods**

The Behaviour Change Wheel (BCW) was used to guide the intervention development process [5]. First, we identified target behaviours through a review of current evidence. Next, we drew on baseline qualitative data with patients (*n* = 10), GPs (*n* = 30) and physiotherapists (*n*=12) to identify determinants of behaviour using the Theoretical Domains Framework [6], mapping these to behaviour change techniques (BCTs) to develop intervention content. Finally, we carried out stakeholder consultation with groups of patients (*n* = 6) and HCPs (*n*=12) regarding the feasibility, acceptability, and local relevance of intervention components.

**Results**

The final intervention, targeting both HCPs and patients, incorporated a range of BCTs. The implementation strategy compromised of an outreach visit with GP training, provision of a GP resource pack for patient consultations, and support from a bespoke online resource. This strategy also facilitated early access to a physiotherapy session, focused on boosting patients’ self-efficacy and self-management skills. Patient behaviors were also targeted with a non-surgical management plan agreed at the first consult, and provision of extra supports around exercise adherence.

**Conclusions**

This study used a systematic theory based approach, incorporating multiple stakeholder perspectives, to develop an intervention for DMT. Implementing evidence based approaches, and thereby reducing low value surgical care, could help sustain a health system under increasing strain to provide care for chronic musculoskeletal conditions.

**Trial Registration:** Not applicable

**Consent to publish:** Not applicable

**References**

1. Siemieniuk RAC, Harris IA, Agoritsas T, Poolman RW, Brignardello-Petersen R, Van de Velde S, et al. Arthroscopic surgery for degenerative knee arthritis and meniscal tears: a clinical practice guideline. BMJ. 2017 May 10;j1982.
2. Ashmore K, Smart K, O’Toole G, Doody C. Triage of knee pain by an Extended Scope Physiotherapist (ESP) in an orthopaedic clinic: A clinical audit. Physiother Pract Res. 2014 Jan 1;35(1):25–32.
3. LaPrade MD, Camp CL, Krych AJ, Werner BC. Analysis of Charges and Payments for Outpatient Arthroscopic Meniscectomy From 2005 to 2014: Hospital Reimbursement Increased Steadily as Surgeon Payments Declined. Orthop J Sports Med. 2021 Jun 1;9(6):23259671211010480.
4. Chan EW, Chaulk RC, Cheng Y, Shin J. No decrease in incidence of arthroscopic meniscectomy in a Canadian province. Knee Surg Sports Traumatol Arthrosc. 2021 Dec 1;29(12):4223–31.
5. Michie S, van Stralen MM, West R. The behaviour change wheel: A new method for characterising and designing behaviour change interventions. Implement Sci. 2011 Dec;6(1):42.
6. Cane J, O’Connor D, Michie S. Validation of the theoretical domains framework for use in behaviour change and implementation research. Implement Sci. 2012 Dec;7(1):37.

## P61: Implementing a web-based application for men’s health screening in a primary care setting during the Covid-19 pandemic: a mixed-methods pilot study

### Chor Yau Ooi^1,2^, Chirk Jenn Ng^3^, Anne E. Sales^4^, Chin Hai Teo^1^

#### ^1^Department of Primary Care Medicine, University of Malaya, 50603, Kuala Lumpur, Malaysia; ^2^Department of Family Medicine, Universiti Malaysia Sarawak, 94300, Kota Samarahan, Malaysia; ^3^Duke-NUS Medical School, 8 College Rd, Singapore 169857; ^4^Sinclair School of Nursing and Department of Family and Community Medicine, University of Missouri, Columbia, MO 65211, USA

##### **Correspondence:** Chor Yau Ooi (cyooi@unimas.my)

*Implementation Science 2024*, **19(1)**:P61

**Background**

Men’s use of health screening remains low globally [1]. This was more evident during the Covid-19 pandemic as most non-urgent services in the clinic were halted, including health screening. Technology can be used to overcome barriers to screening by improving accessibility, motivating and reminding individuals to get screened. ScreenMen is a web-based application that was developed to increase the uptake of men's health screening. This study was a process evaluation of the implementation of ScreenMen in a primary care setting.

**Methods**

This study was conducted in a government health clinic using a mixed-method explanatory sequential design driven by the RE-AIM (Reach, Effectiveness, Adoption, Implementation, Maintenance) framework [2]. We used a tailored intervention including: mandate change, provide education and training, identify and prepare champions, use of information and communication technology, and audit and provide feedback. Participants were staff and patients. We used Google Analytics to monitor patient uptake of ScreenMen for 5 months and conducted staff interviews to understand the implementation process. We used template analysis based on the RE-AIM framework [3].

**Results**

A total of 73 patients accessed the app. Access was higher as implementation started but subsequently dropped and increased again towards the end of the period. The majority (41%) of patients accessed the app through QR codes. In qualitative analysis we found that access was lower than expected because of decreased patients in the clinic during the pandemic. The later increase in access was related to champion activity. Bunting promotes access due to its size and strategic placement. Staff found that mandated change was not useful as an implementation strategy.

**Conclusions**

Making patients access the app in the clinic and using bunting were reported to be effective in implementing ScreenMen while mandate change was found to be least helpful.

**Trial Registration:** Non applicable

**Consent to publish:** Non applicable

**References**

1. Teo CH, Ling CJ, Ng CJ. Improving Health Screening Uptake in Men: A Systematic Review and Meta-analysis. American journal of preventive medicine. 2018;54(1):133-43.
2. Glasgow RE, Vogt TM, Boles SM. Evaluating the public health impact of health promotion interventions: the RE-AIM framework. Am J Public Health. 1999;89(9):1322-7.
3. QualRIS. Qualitative Methods in Implementation Science 2019 [Available from: https://cancercontrol.cancer.gov/IS/docs/NCI-DCCPS-ImplementationScience-WhitePaper.pdf]

## P62: Developing a theory of change for implementing the novel UK Children’s Palliative care Outcome Scale (C-POS:UK) into routine paediatric palliative care

### Hannah M Scott^1^, Debbie Braybrook^1^, Daney Harðardóttir^1^, Inez Gaczkowska^1^, Clare Ellis-Smith^1^, Lorna K Fraser^1^, Fliss EM Murtagh^2^, Richard Harding^1^, on behalf of C-POS

#### ^1^Florence Nightingale Faculty of Nursing Midwifery and Palliative Care, Cicely Saunders Institute, King’s College London, London, SE59PJ, United Kingdom; ^2^Hull York Medical School, Wolfson Palliative Care Research Centre, Hull, HU6 7RX, United Kingdom

##### **Correspondence:** Hannah M Scott (hannah.m.scott@kcl.ac.uk)

*Implementation Science 2024*, **19(1)**:P62

**Background**

To ensure a newly developed measure (C-POS:UK) can be successfully implemented, it is important to draw together existing evidence and work collaboratively with key stakeholders to develop an experience-informed, evidence-based Theory of Change.

**Methods**

The STRiDE guidance [1] for Theory of Change Workshops was used to inform the workshops. Parents of children with life-limiting or life-threatening conditions and health and social care professionals working in the NHS were recruited through social media and networks. Theory of Change Maps were developed and refined based on stakeholder contributions using a template adapted from Stories for Impact [2].

**Results**

Eight multi-disciplinary professionals and four parents took part in the workshops. Both parents and professionals felt the long-term goal of implementing a measure would be improving care and comfort for children and their families. Professionals emphasised the importance of having adequate, staffing, time and monetary resources, as well as the importance of education and training on using the measure, and reminders or prompts to help them remember to use it. Parents felt a pre-requisite to the successful implementation of a measure was for all professionals to have an understanding of what palliative care is. Parents wanted a trusted professional to support completion of the measure and nurses were identified as most appropriate due to the relationship they had with families. Parents also highlighted a need to improve communication and information sharing to avoid children and families having to answer distressing questions or share their stories multiple times and professionals also felt the measure may help facilitate this.

**Conclusion**

These workshops have supported the development of a UK context-specific, evidence-based and experience-informed Theory of Change and will inform the development of an implementation plan for C-POS:UK. Future work will involve the review of the Theory of Change as part of a pilot study to test the Implementation Plan in practice.

**Trial Registration:** Not applicable

**Consent to publish:** Yes

**References**

1. Breuer E, Comas-Herrera A, Docrat S, Freeman E, Schneider M, the STRiDE team. STRiDE Theory of Change Workshops: Guidance and Resources. London: Care Policy and Evaluation Centre; 2019.
2. Stories for Impact. Theory of Change. Online; 2023 [Accessed 01 April 2023; Available from: https://storiesforimpact.com/toolbox/theory-of-change/].

## O63: An approach for developing a tailored implementation intervention to implement a web-based application for men’s health screening in a primary care setting during the Covid-19 pandemic

### Chor Yau Ooi^1,2^, Chirk Jenn Ng^3^, Anne E. Sales^4^, Chin Hai Teo^1^

#### ^1^Department of Primary Care Medicine, University of Malaya, 50603, Kuala Lumpur, Malaysia; ^2^Department of Family Medicine, University Malaysia Sarawak, 94300, Kota Samarahan, Malaysia; ^3^Duke-NUS Medical School, 8 College Rd, Singapore 169857; ^4^Sinclair School of Nursing and Department of Family and Community Medicine, University of Missouri, Columbia, MO 65211, USA

##### **Correspondence:** Chor Yau Ooi (cyooi@unimas.my)

*Implementation Science 2024*, **19(1)**:O63

**Background**

A systematic review reported that tailoring implementation strategies to address the determinants of practice is effective [1]. In this study, we propose an approach to develop a package of tailored strategies to implement a web-based application (ScreenMen) for men’s health screening in Kuala Lumpur, Malaysia.

**Methods**

The development process of the tailored implementation intervention is described in Figure 1.

**Results**

A total of 58 approaches were generated and mapped to ERIC strategies. Subsequently, we selected 9 strategies based on their appropriateness and feasibility: involve executive boards, mandate change, provide education and training, create new clinical teams, identify and prepare champions, the use of information and technology, remind clinician, audit and provide feedback, and alter incentives/allowance structures. Following the evaluation using APEASE criteria, we removed 3 implementation strategies. The final tailored implementation intervention consisted of 6 implementation strategies: involve executive boards, mandate change, provide education and training, identify and prepare champions, use of information and communication technology, and audit and provide feedback.

**Conclusions**

Using a systematic method enabled the development of a tailored implementation intervention to implement a web-based application for screening, even during a pandemic.

**Trial Registration:** Non applicable

**Consent to publish:** Non applicable

**References**

1. Baker R, Camosso‐Stefinovic J, Gillies C, Shaw EJ, Cheater F, Flottorp S, et al. Tailored interventions to address determinants of practice. Cochrane Database of Systematic Reviews. 2015(4).
2. Grol R, Wensing M, Eccles M, Davis D. Improving Patient Care: The Implementation of Change in Health Care, Second Edition. 2nd ed: John Wiley & Sons, Ltd; 2013 28 June 2013.
3. Powell BJ, Waltz TJ, Chinman MJ, Damschroder LJ, Smith JL, Matthieu MM, et al. A refined compilation of implementation strategies: results from the Expert Recommendations for Implementing Change (ERIC) project. Implementation Science. 2015;10(1):21.
4. Proctor EK, Powell BJ, McMillen JC. Implementation strategies: recommendations for specifying and reporting. Implement Sci. 2013;8:139.
5. Michie S, Ashford S, Sniehotta FF, Dombrowski S, Bishop A, French D. The Behaviour Change Wheel: A Guide to Designing Interventions: Great Britain: Silverback Publishing; 2014.

**Fig. 1 (Abstract O63).**
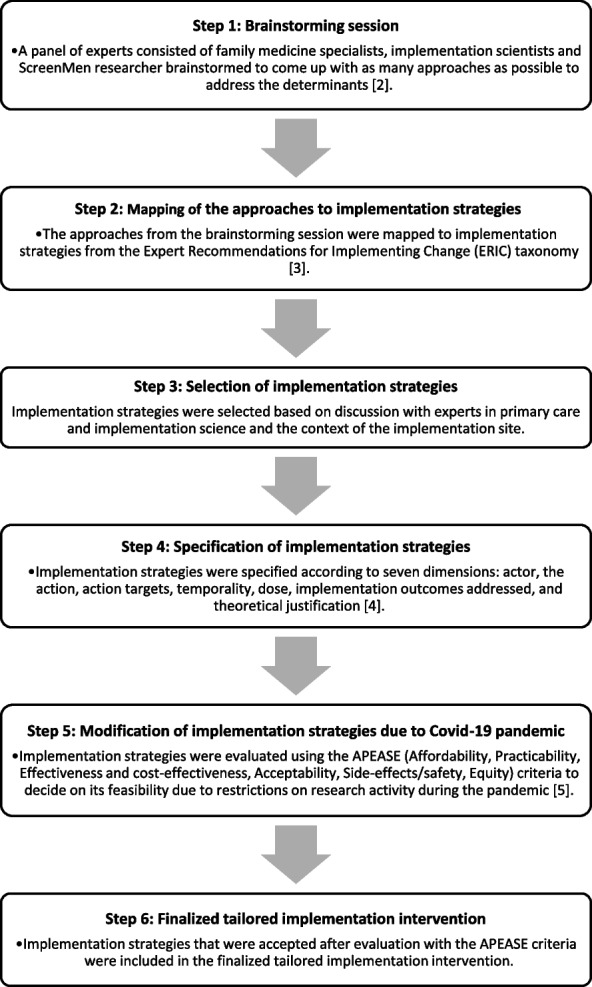
An approach for the development of a tailored implementation intervention for ScreenMen

## O64: Scaling -up a virtual culturally tailored diabetes self-management programme for African and Caribbean communities (HEAL-D Online) across NHS regions in England: a qualitative study using the EPIS framework

### Sophie Lowry^1^, Joseph T S Low^1^, Louise Goff^2^, Sally Irwin^1^, Oliver Brady^1^, Natasha Curran^1^, Nick Sevdalis^3^, Andrew Walker^1^

#### ^1^Health Innovation Network, London, UK; ^2^Department of Health Sciences, University of Leicester, Leicester, UK; ^3^Centre for Behavioural and Implementation Science Interventions, National University of Singapore, Singapore

##### **Correspondence:** Sophie Lowry (Sophie.lowry2@nhs.net)

*Implementation Science 2024*, **19(1)**:O64

**Background**

Healthy Eating and Active Lifestyles for Diabetes (HEAL-D) is a culturally tailored self-management education programme co-designed with, and for, African and Caribbean adults with Type 2 diabetes. Developed as a face-to-face intervention, it is now delivered virtually as ‘HEAL-D Online’.

This study explores the implementation and adoption of HEAL-D Online in other English regions by understanding the factors affecting scale-up from operational delivery and commissioning perspectives.

**Method**

We conducted focus groups with 15 members of the public of African and Caribbean heritage, and interviews with 6 commissioners and 3 diabetes service providers in three Integrated Care Systems outside of London.

Data was analysed using thematic analysis. The Exploration-Preparation-Implementation-Sustainment’ (EPIS) framework informed the analysis approach, focusing on the ‘Exploration’ stage to consider how HEAL-D Online can address a clinical need whilst considering the contextual factors supporting or hindering implementation.

**Results**

Focus group findings identified most participants were accustomed to using online platforms, with individuals requesting education on topics covered by HEAL-D Online, suggesting that scaling HEAL-D Online would be acceptable.

Commissioners and service providers highlighted a lack of existing culturally tailored services, and a clear understanding of the benefits that HEAL-D Online, or a similar virtual, culturally tailored programme could offer. Commissioning processes and service capacity varied, though all wanted to understand more around local demand and the clinical and cost-effectiveness of the intervention.

Using EPIS, ‘Client Advocacy’ (patient needs) was identified as a key implementation enabler, whilst ‘Funding’ (cost of the intervention vs. available funding), ‘Interorganisational Networks’ (system priorities and relationships) and ‘Patient/Client Characteristics’ (size of target population) were potential barriers.

**Conclusion**

There is strong interest in further exploring population need and scaling of HEAL-D Online in other areas of England, but a key challenge to any virtual scale-up is digital poverty. Addressing this will be required to ensure successful implementation.

**Trial Registration:** Not applicable

**Consent to publish:** Yes

## P65: HEAL-D online – is it feasible to implement a virtual culturally tailored diabetes self-management programme for African and Caribbean communities in south London

### Joseph T S Low^1^, Sophie Lowry^1^, Louise Goff^2^, Sally Irwin^1^, Oliver Brady^1^, Natasha Curran^1^, Nick Sevdalis^3^, Andrew Walker^1^

#### ^1^Health Innovation Network, London, UK; ^2^Department of Health Sciences, University of Leicester, Leicester, UK; ^3^Centre for Behavioural and Implementation Science Interventions, National University of Singapore, Singapore

##### **Correspondence:** Joseph T S Low (joseph.low1@nhs.net)

*Implementation Science 2024*, **19(1)**:P65

**Background**

Type 2 diabetes is a major health concern for UK African and Caribbean people. To tackle ethnic inequalities in diabetes healthcare access, a virtual culturally tailored diabetes self-management education programme (HEAL-D Online) was rolled out in South London for this community. We present findings from our evaluation assessing the acceptability and feasibility of implementing this new programme.

**Method**

Mixed methodology. Quantitative: Service activity data assessed service user engagement, acceptability and benefit (self-reported weight loss and diabetes-related emotional distress). Data was analysed using frequencies and percentages. Qualitative: Semi-structured interviews were conducted with 14 service users and 7 service delivery staff to explore their perceptions of the feasibility and acceptability of HEAL-D Online, and data collected was analysed using Framework Methodology. Fidelity was measured through observations using a fidelity checklist.

**Results**

Service activity data showed that initial uptake of HEAL-D Online was low but once patients attended their first session, there was a completion rate of 77%, demonstrating high adherence. A high fidelity was observed, and qualitative findings showed that staff and service users were satisfied with all aspects of course delivery. Both service activity and qualitative data indicated that attendees felt more confident in controlling their diet and managing their diabetes post-HEAL-D, with many reporting a reduction both in weight and diabetes-related psychological distress.

**Conclusion**

HEAL-D Online was well received by attendees with a high completion rate. It was successful in its goals of providing attendees with the knowledge and necessary skills to elicit behavioural change to support their diabetes management, ultimately leading to weight loss in some attendees. Challenges were identified around the identification, recruitment and referral of eligible patients into the programme and these need to be addressed for successful implementation of this programme on a wider scale.

**Trial Registration:** Not applicable (not an RCT)

**Consent to publish:** Yes

## P66: A qualitative analysis of experiences, enablers and barriers to implementing nationally endorsed health and social care standards in Ireland

### Yvonne Kelly^1^, Niamh O’Rourke^2^, Rachel Flynn^1^, Josephine Hegarty^3^, Laura M Keyes^1^

#### ^1^Health Information and Standards Directorate, Health Information and Quality Authority, Unit 1301, Citygate, Mahon, Cork, T12 Y2XT, Ireland; ^2^Health Information and Standards Directorate, Health Information and Quality Authority, George's Court, George's Lane, Dublin 7, D07 E98Y, Ireland; ^3^Catherine McAuley School of Nursing and Midwifery, Brookfield Health Sciences Complex, University College Cork, College Road Cork, T12 AK54, Ireland

##### **Correspondence:** Yvonne Kelly (ykelly@hiqa.ie)

*Implementation Science 2024*, **19(1)**:P66

**Background**

Health and social care standards are complex interventions that describe safe, high-quality care. They require multiple collective actions from multiple stakeholders across diverse services in health systems [1]. Standards are typically enforced or encouraged through statutory requirements or quality improvement initiatives. Limited evidence exists on appropriate implementation strategies to enhance their implementation [2,3]. We aimed to explore experiences of implementing nationally endorsed standards from stakeholders working at multiple levels in the health system, and to identify enablers and barriers to effective implementation. This exploration will inform implementation strategies that can optimise standards implementation.

**Methods**

Using a descriptive qualitative design, six focus groups and eight individual interviews were conducted with stakeholders at individual-level (*n*=10), organisational-level (*n*=14) and system-level (*n*=14). Discussions were audio-recorded with consent, transcribed verbatim and analysed using Braun & Clarke’s reflexive thematic analysis [4]. Interpretation of data was underpinned by social constructionism [5]. Collective reflexivity and Lincoln & Guba’s criteria of trustworthiness was used to enhance rigour [6].

**Results**

Six themes were generated from patterns of shared meanings across participants’ stories. Participants reported that implementation should incorporate: a “top-down, bottom-up approach”, accessible “bite-size” support tools, “meaningful” language, and “leaders at every single level.” An enabler to implementing standards was collegial support from the regulatory body that included reassurance that services were “doing the right thing.” A barrier was a “tired, worn out” workforce.

**Conclusions**

Themes generated described intervention and organisational characteristics that are reflected in existing implementation determinant frameworks. A novel finding was how an external organisation such as a regulatory body can influence implementation of standards. Few determinant frameworks address external organisational influences [7]. Adopting organisational theory may help to better understand these external influences on implementation in health and social care services. Findings from this exploration can be used by researchers to inform implementation strategies that can optimise safe, high-quality care delivery.

**Trial Registration:** Not applicable

**Consent to publish:** All study participants gave written informed consent for the publication of research outputs relating to this study.

**Acknowledgements**

This work was conducted as part of the Structured Population Health, Policy and Health-services Research Education (SPHeRE) programme (Grant No. SPHeRE/2019/1). Yvonne Kelly has conducted this work as part of a PhD studentship that is funded by the Health Information and Quality Authority (HIQA).

**References**

1. Kelly Y, O'Rourke N, Flynn R, Hegarty J, O'Connor L. Definitions of health and social care standards used internationally: A narrative review. Int J Health Plann Manage. 2023;38(1):40-52.
2. Proctor EK, Powell BJ, McMillen JC. Implementation strategies: recommendations for specifying and reporting. Implement Sci. 2013;8(1):139.
3. Powell BJ, Beidas RS, Lewis CC, Aarons GA, McMillen JC, Proctor EK, et al. Methods to Improve the Selection and Tailoring of Implementation Strategies. J Behav Health Serv Res. 2017;44(2):177-94.
4. Braun V, Clarke V. One size fits all? What counts as quality practice in (reflexive) thematic analysis? Qual. Res. Psychol. 2021;18(3):328-52.
5. Burr V. An Introduction to Social Constructionism. Routledge; 1995.
6. Lincoln Y, Guba E. Naturalistic inquiry. Beverly Hills: Sage Publications. 1985.
7. Nilsen P, Bernhardsson S. Context matters in implementation science: a scoping review of determinant frameworks that describe contextual determinants for implementation outcomes. BMC Health Serv. Res. 2019;19(1):189-99.

## O67: Pathfinding, peace-making, power, and passion: exploring the lived experience of facilitation during implementation of Canada’s mental health recovery guidelines

### Myra Piat^1^, Marie-Pier Rivest^2^, Ian Graham^3^, Helene Albert^2^, Lucy Melville^4^, Megan Wainwright^5^, Eleni Sofouli^6^, Kanwar Singh^6^, Stephanie Vasko^7^

#### ^1^Department of Psychiatry, McGill University, Montreal, Canada; ^2^Douglas Mental Health University Institute, Montreal, Canada; ^3^Social Work Department, Université de Moncton, Moncton, Canada; ^4^School of Epidemiology & Public Health, University of Ottawa, Ottawa, Canada; ^5^Bangor University, Wales, UK; ^6^Durham University, Durham, UK; ^7^York University, Toronto, Canada

##### **Correspondence:** Lucy Melville (l.melville-richards@bangor.ac.uk)

*Implementation Science 2024*, **19(1)**:O67

**Background**

We build on a 5-year project to implement Canada’s mental health recovery guidelines using the co-produced Walk the Talk Toolkit (https://walkthetalktoolkit.ca). Facilitation is explored from multiple stakeholder perspectives to embed lived experience within the Toolkit, enhancing inclusivity.

**Methods**

This pan-Canadian qualitative study explores facilitation as an active and ongoing process. 40 interviews with those who use and deliver services across 7 mental health organisations, alongside facilitators were conducted. Ways of improving facilitation from each stakeholder’s perspective, during planning, implementation, and coaching were elicited. Thematic analysis reveals what is important to stakeholders during facilitation, and how this can be used to enhance the experience and outcomes of future implementation efforts.

**Results**

Emergent findings revolve around themes of people, process, pitfalls, and payoff. A safe space for those in recovery to engage in implementation is necessary. Conviction, cultural competence, and a nurturing approach are valued facilitator attributes. Establishing parity amongst stakeholders, striking a ‘sweet spot’ between being directive and enabling, alongside resilience and mediation, are helpful during coaching. Momentum and motivation are improved via the prospect of tangible outcomes. Despite efforts to gamify the CFIR, the language of implementation science remains baffling to many. Questions about whether the Toolkit, recovery principles, and implementation science itself represent boundary objects are surfacing.

**Conclusions**

Co-producing implementation toolkits needs meaningful engagement at all levels with all stakeholders. Generating ownership during coaching improves success of recovery-oriented interventions, but a shift in leadership can be challenging. Engaging in successful implementation can initiate a legacy of change at an individual and collective level. Work with equity deserving groups including indigenous and LGBTQ+ communities to improve cultural inclusivity is underway. Scaling up across international health and social care is planned.

**Trial Registration:** Not applicable

**Consent to publish:** Not applicable

## O68: Minding the gap: the importance of active facilitation in moving boundary objects from in-theory to in-use as a tool for knowledge mobilisation

### Shaima M. Hassan^1,2^, Lucy Melville-Richards^3^, Adele Ring^1,2^, Jane Cloke^1,2^, Sandra Smith^2^, Pooja Saini^4^, Mark Goodall^1,2^, Ana Porroche-Escudero^5^, Jennie Popay^5^, Mark Gabbay^1,2^

#### ^1^Institute of Population Health Sciences, University of Liverpool, Liverpool, UK; ^2^NIHR Applied Research Collaboration ARC NWC, Liverpool, UK; ^c^School of Health Science, Bangor University, UK; ^4^Department of Psychology, Faculty of Health, Liverpool John Moores University, UK; ^5^Lancaster university, Lancaster, UK

##### **Correspondence:** Jane Cloke (jcloke@liverpool.ac.uk)

*Implementation Science 2024*, **19(1)**:O68

**Link to publication**

## O69: The Mental Health Care in Primary healthcare project (SMAPS) implementation assessment research: Stakeholders analysis protocol

### llana Eshriqui^1,2^, Luciana Cordeiro^1,2^, Ana Alice Freire de Sousa^1,2^, Daiana Bonfim^1,2^, Letícia Yamawaka de Almeida^1,2^

#### ^1^Hospital Israelita Albert Einstein, São Paulo, São Paulo, Brazil; ^2^Albert Einstein Center for Studies Research and Practice in PHC (CEPPAR), São Paulo, São Paulo, Brazil

##### **Correspondence:** llana Eshriqui (ilana.eoliveira@einstein.br)

*Implementation Science 2024*, **19(1)**:O69

**Background**

Stakeholders play a central role in an implementation intervention [1]. SMAPS is developed in three Brazilian states using the Health Care Planning (HCP) [2] methodology and the mi-mhgap trainning [3] to support the Mental Health Care in Primary Health Care (PHC). This study aims to present a protocol to determine stakeholders’ analysis concerning their relevance and influence on SMAPS.

**Method**

We developed a standard script to guide focus groups that will be composed of SMAPS’s: i. proponents and ii. local stakeholders, including high and mid-level leaders. The Power/Interest Matrix [4] will be used to enable stakeholder analysis.

**Results**

Three focus groups scripts will be virtually developed with a mean duration of 90 minutes. Focus groups will be composed of five moments, allowing the construction of a mental map at the end of the activity: i. presentations; ii. Stakeholders concept comprehension and discussion; iii) Power and interest concepts discussion; iv) Silent moment to individually fill in the power/interest matrix; v) Group discussion and consensus. Content analysis will be carried out from each group through the mental maps (Figure 1), audio recording and researchers’ observation.

**Conclusion**

The health care field has not yet systematized the stakeholder analysis methods. The Power/Interest Matrix can be a relevant tool in health interventions implementation research and can be used to plan the intervention by its proponents, aiming stakeholders’ engagement and implementation success.

**Trial Registration:** Non applicable

**Consent to publish:** Non applicable

**References**

1. Brugha R, Varvazovszk Z. Stakeholder analysis: a review. Health Policy Plan. 2000;15(3):239-246.
2. Evangelista MJO, Guimarães AMDN, Dourado EMR, Vale FLBD, Lins MZS, Matos MAB, Silva RBMDPM, Schwartz SA. Planning and building Health Care Networks in Brazil’s Federal District. Cien Saude Colet. 2019;24(6):2115-2124.
3. World Health Organization. MhGAP training manuals for the mhGAP intervention guide for mental, neurological and substance use disorders in non-specialized health settings, version 2.0 (for field testing). 2017. [Avaiable at: https://apps.who.int/iris/handle/10665/259161]. Licença: CC BY-NC-SA 3.0 IGO.
4. Johnson G, Scholes K, Wittington R. Exploring Corporate Strategy. 8ed. England: Pearson Education; 2008.

**Fig. 1 (Abstract O69).**
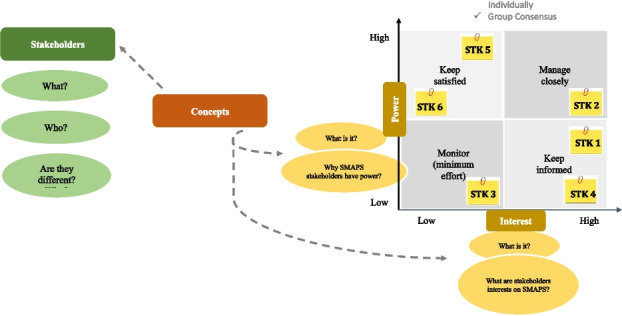
Mental map to SMAPS stakeholder analysis

## P70: Using the consolidated framework of implementation research and implementation outcomes to develop interview scripts: methodological insights from the Mental Health in Primary Health Care project (SMAPS) implementation assessment research

### llana Eshriqui^1,2^, Luciana Cordeiro^1,2^, Ana Alice Freire de Sousa^1,2^, Daiana Bonfim^1,2^, Letícia Yamawaka de Almeida^1,2^

#### ^1^Hospital Israelita Albert Einstein, São Paulo, São Paulo, Brazil; ^2^Albert Einstein Center for Studies Research and Practice in PHC (CEPPAR), São Paulo, São Paulo, Brazil

##### **Correspondence:** llana Eshriqui (ilana.eoliveira@einstein.br)

*Implementation Science 2024*, **19(1)**:P70

**Background**

The SMAPS is developed in three Brazilian states aiming to support mental health care in Primary Health Care (PHC), using the Health Care Planning method (HCP) [1] and MHGap training as central implementation strategies [2]. This work aims to present the process of preparing qualitative data collection scripts used to interview multiple levels of SMAPS stakeholders in an implementation assessment research.

**Methods**

The value of the collected data depends on the strength of the interview questions to capture meanings and experiences [3]. Thus, scripts to different levels of stakeholders were constructed through a collaboration between researchers and SMAPS proponents, considering the potential of each question to inform implementation outcomes [4] and determinants according to Consolidated Framework of Implementation Research (CFIR) [5].

**Results**

Scripts were constructed considering four levels of SMAPS stakeholders, who perform different roles in state and municipality government levels (Figure 1). When asked to different stakeholder levels, the same question shows potential to inform different implementation outcomes. However, questions for Innovation delivers and receivers show potential to capture more implementation outcomes then for high and mid-leaders’ levels. All questions show potential to inform the same CFIR determinants independently of stakeholder level.

**Conclusion**

Beyond being useful to guide research analysis, implementation outcomes and CFIR are useful to construct implementation research scripts and to training interviewers.

**Trial Registration:** Not applicable

**Consent to publish:** Not applicable

**References**

1. Evangelista MJO, Guimarães AMDN, Dourado EMR, Vale FLBD, Lins MZS, Matos MAB, Silva RBMDPM, Schwartz SA. Planning and building Health Care Networks in Brazil's Federal District. Cien Saude Colet. 2019; 27;24(6):2115:2124.
2. World Health Organization. MhGAP training manuals for the mhGAP intervention guide for mental, neurological and substance use disorders in non-specialized health settings, version 2.0 (for field testing). 2017; https://apps.who.int/iris/handle/10665/259161. Licença: CC BY-NC-SA 3.0 IGO
3. Roberts, R. E. Qualitative Interview Questions: Guidance for Novice Researchers. The Qualitative Report. 2020; 25(9), 3185:3203
4. Proctor E, Silmere H, Raghavan R, Hovmand P, Aarons G, Bunger A, Griffey R, Hensley M. Outcomes for implementation research: conceptual distinctions, measurement challenges, and research agenda. Adm Policy Ment Health. 2011; 38(2):65:76.
5. Damschroder LJ, Aron DC, Keith RE, Kirsh SR, Alexander JA, Lowery JC. Fostering implementation of health services research findings into practice: a consolidated framework for advancing implementation science. Implement Sci. 2009; 7;4:50.

**Fig. 1 (Abstract P70).**
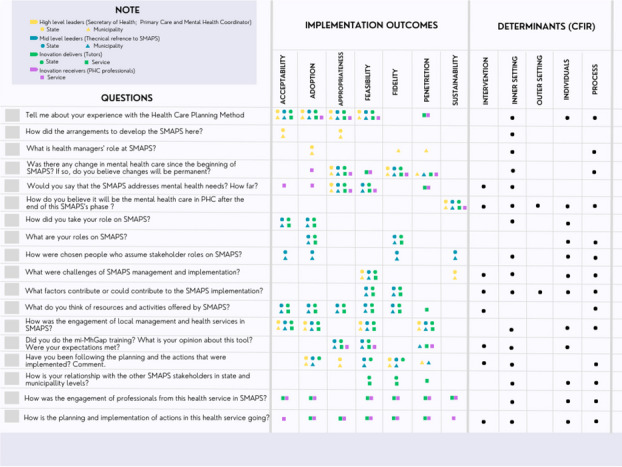
Examples of scripts’ questions and their relation with CFIR determinants and implementation outcomes of interest

## P71: Design and implementation of an online older person/student nurse intergenerational café using CFIR

### Dympna Tuohy^1^, Irene Cassidy^1^, Eileen Carey^1^, Margaret Graham^1^, Jan McCarthy^1^, Kellie Morrissey^2^, Jill Murphy^1^, Jacinta Shanahan^3^, Teresa Tuohy^1^

#### ^1^Department of Nursing and Midwifery, University of Limerick, Limerick, Ireland; ^2^School of Design, University of Limerick, Limerick, Ireland; ^3^Office of the President, University of Limerick, Limerick, Ireland

##### **Correspondence:** Dympna Tuohy (dympna.tuohy@ul.ie)

*Implementation Science 2024*, **19(1)**:P71

**Background**

Most older people live independently but at times may require nursing care either at home, in primary and community care, in hospital or in nursing homes. Most nurses will care for older people during their career. It is crucial that older people and nurses can work together in caring partnerships. Student nurses need to be supported to develop this knowledge and skill. In effort to break down barriers, promote respect, build links and promote understanding between generations, it is useful to develop ways to increase intergenerational learning and connections. The aim of this research was to determine the feasibility of using intergenerational discussion cafés as an implementation strategy.

**Method**

Ethically approved research guided by the Consolidated Framework for Implementation Research (CFIR)[1]. Online intergenerational discussion cafés were held for 3^rd^ year-student nurses and older people. Participants were invited post café to participate in an anonymous online survey with student nurses (*n*=50) older people (*n*=49) and facilitators (*n*=9) responding. Data were collected through survey questionnaires (descriptive statistical and thematic data analysis ) and facilitator reflections. Post hoc ‘CFIR’ analysis using adapted codebooks was undertaken to evaluate the café implementation.

**Results**

Organisational factors (e.g. clear instructions, being organised and sufficient time) are important for the effective implementation. More students than older people felt that the purpose, topics and online running of the café were clear and organised. More older people than students wanted more time in the discussion groups and some of this cohort experienced technical difficulties. All were positively disposed to the cafés as a way of increasing intergenerational learning. Facilitator teamwork enable smooth running of the cafés.

**Conclusion**

This intervention was worthwhile as it facilitated mutual learning and understanding. Intergenerational cafés are now embedded in the BSc Nursing (General, Intellectual Disability and Mental Health) curricula.

**Trial Registration:** Not applicable

**Consent to publish:** Not applicable

**Reference**

1. Damschroder, L.J., Aron, D.C., Keith, R.E., Kirsh, S.R., Alexander, J.A. & Lowery, J.C. Fostering implementation of health services research findings into practice: a consolidated framework for advancing implementation science, Implementation Science*,* 2009: 4,50, available: doi:10.1186/1748-5908-4-50.

## O73: Exploring MECC implementation within the North East and North Cumbria region (NENC) in England

### Angela Rodrigues^1^, Bethany Nichol^2^, Rob Wilson^3^, Caroline Charlton^1^, Beckie Gibson^1^, Tracy Finch^4^, Catherine Haighton^2^, Gregory Maniatopoulos^5^, Emma L. Giles^6^, Deborah Harrison^7^, Denise Orange^8^, Craig Robson^9^, Jill Harland^9^

#### ^1^Department of Psychology, Northumbria University, Northumberland Building, Newcastle upon Tyne, NE1 8ST, UK; ^2^Department of Social Work, Education and Community Wellbeing, Northumbria University, Coach Lane Campus West, Newcastle Upon Tyne, NE7 7XA; ^3^Newcastle Business School, Northumbria University, Newcastle-upon-Tyne, NE1 8ST, UK; ^4^Department of Nursing, Midwifery and Health, Northumbria University; ^5^University of Leicester School of Business, University of Leicester, Leicester, United Kingdom; ^6^Teesside University, School of Health and Life Sciences, Middlesborough, TS1 3BX, UK; ^7^Newcastle University Business School, Newcastle University, Newcastle Upon Tyne, NE1 4AX, UK; ^8^Office for Health Improvement & Disparities, Newcastle upon Tyne, NE15 8NY, UK; ^9^Northumbria Healthcare NHS Foundation Trust, North Tyneside General Hospital, NE29 8NH, UK

##### **Correspondence:** Angela Rodrigues (angela.rodrigues@northumbria.ac.uk)

*Implementation Science 2024*, **19(1)**:O73

**Background**

The Making Every Contact Count (MECC) programme provides training and materials to support public-facing workers to encourage health-promoting behaviour change by utilising the day-to-day interactions between organisations and individuals[1]. The project aimed to analyse MECC implementation, delivery models, service reach and system-level relationships within the North East and North Cumbria region (NENC) in England.

**Methods**

A four-part multi-method process evaluation was conducted. MECC programme documents were reviewed and mapped against specific criteria (e.g implementation strategies[2]; MECC implementation guide). An online mapping survey was conducted to establish current implementation/delivery of MECC within NENC settings (e.g local authority, NHS, and voluntary sector). Qualitative research, using individual interviews and group discussions, was conducted to establish further understanding of MECC implementation. A realist approach was utilised[3], applying Normalisation Process Theory[4], Theoretical Domains Framework[5], and Consolidated Framework for Implementation Research[6].

**Results**

Our findings were informed by reviewing five documents, survey participants (*n* = 19), interviews (*n* = 18), and three group discussions. Overall, the implementation of MECC within the region was in an early stage, with training mostly delivered between rather than within organisations. The qualitative findings highlighted factors that encourage stakeholders to implement MECC (e.g organisational goals that were facilitated by MECC implementation, including the prevention agenda), supporting resources that facilitate the implementation MECC (e.g logic models), and enabling factors that promote MECC sustainability across the region (e.g buy-in from leadership and management).

**Conclusion**

The NENC MECC programme is built around regional leadership that supports the implementation process. This process evaluation of the implementation of MECC identified multi-level barriers and facilitators to MECC implementation across the region. Our recommendation for policy and practice can be taken forward to develop targeted strategies to support future MECC implementation. For example, a standardised infrastructure and strategy is needed to combat delivery and implementation issues identified.

**Trial Registration:** Not applicable

**Consent to publish:** Not applicable

**References**

1. Public Health England (2016). *Making Every Contact Count (MECC): evaluation framework*. March 2016, PHE. Publication number 2015744.
2. Powell BJ, Waltz TJ, Chinman MJ, Damschroder LJ, Smith JL, Matthieu MM, Proctor EK, Kirchner JE. A refined compilation of implementation strategies: results from the Expert Recommendations for Implementing Change (ERIC) project. Implementation science. 2015 Dec;10(1):1-4.
3. Pawson R, Tilley N. An introduction to scientific realist evaluation. Evaluation for the 21st century: A handbook. 1997 Jan 28;1997:405-18.
4. May C, Finch T. Implementing, embedding, and integrating practices: an outline of normalization process theory. Sociology. 2009 Jun;43(3):535-54.
5. Cane J, O’Connor D, Michie S. Validation of the theoretical domains framework for use in behaviour change and implementation research. Implementation science. 2012 Dec;7:1-7.
6. Birken SA, Powell BJ, Presseau J, Kirk MA, Lorencatto F, Gould NJ, Shea CM, Weiner BJ, Francis JJ, Yu Y, Haines E. Combined use of the Consolidated Framework for Implementation Research (CFIR) and the Theoretical Domains Framework (TDF): a systematic review. Implementation science. 2017 Dec;12:1-4.

## P74: The application of logic models and theories of change to inform healthcare policy: a scoping review

### Maria VM Karadimova-Watts^1^, Andy Bradshaw^2^, Katherine Sleeman^2^

#### ^1^GKT School of Medical Education, King’s College London, London, SE1 1UL, UK; ^2^Cicely Saunders Institute of Palliative Care, Policy & Rehabilitation, King’s College London, London, SE5 9RS, UK

##### **Correspondence:** Maria VM Karadimova-Watts (maria.karadimova-watts@kcl.ac.uk)

*Implementation Science 2024*, **19(1)**:P74

**Background**

Theories of Change and Logic Models are policy-mapping tools to aid understanding of policy. A Theory of Change is ‘a theory of how and why an initiative works’ [1]. Similarly, a Logic Model ‘illustrates how a program is designed to meet its intended outcomes’ [2]. The objectives of this study were to: (i) examine how Theories of Change and Logic Models are currently used in healthcare policy, and (ii) to explore trends in use across time, countries, and research fields.

**Materials and methods**

A scoping review was conducted, using PubMed. Inclusion criteria included mentioning Theories of Change or Logic Models in the context of informing policy, and primary research published within the last 10 years. Exclusion criteria included absence of explanation of ‘policy’, non-healthcare focus and absence of a methods section. A data extraction form was used to extract data on seven outcomes: primary research type, Theory of Change or Logic Model approach, extent of integral use of Theory of Change or Logic Model, revision of the Theory of Change or Logic Model, date of publication, country of publication and topic.

**Results**

346 initial studies were identified, with 25 being included. Results demonstrated 64% of studies implemented a model prior to their research, with 40% of studies revising this initial model after their findings. The highest frequency of models was seen across Africa (28%), although other countries were utilising these (such as America and Australia). 68% of papers focussed on public health.

**Conclusions**

The focus on public health could be potentially attributable to Governmental requirements in some countries to include a Theory of Change or Logic Model in community-based health intervention applications. Further research is recommended to understand the public health focus, as well as how varying the timepoint of model employment may affect the healthcare policy outcome.

**Consent to Publish:** Not applicable

**Trial registration**: Not applicable

**References**

1. De Silva MJ, Breuer E, Lee L, Asher L, Chowdhary N, Lund C, et al. Theory of Change: a theory-driven approach to enhance the Medical Research Council’s framework for complex interventions. Trials. 2014 Jul 5;15:267.
2. Anderson LM, Petticrew M, Rehfuess E, Armstrong R, Ueffing E, Baker P, et al. Using logic models to capture complexity in systematic reviews: Logic Models in Systematic Reviews. Res Syn Meth [Internet]. 2011 Mar [cited 2023 Apr 8];2(1):33–42. Available from: https://onlinelibrary.wiley.com/doi/10.1002/jrsm.32

## O75: Scaling out: spreading the delivery of an advance care planning digital intervention from nursing homes to community care

### Kevin Brazil^1^, Roisin O’Neill^1^, Olivia Jamison^1^, Alice Coffey2, Julie Doherty^1^, Owen Doody^2^, Anne Finucane^3^, Julie Green^4^, Karen Harrison Dening^5^, Gary Mitchell^1^, Nancy Preston^6^

#### ^1^School of Nursing and Midwifery, Queen’s University Belfast, County Antrim, BT9 7BL, Northern Ireland, UK; ^2^Department of Nursing Studies and Midwifery, University of Limerick, County Limerick, Ireland; ^3^School of Health and Social Science, University of Edinburgh, Edinburgh, EH8 9AG, Scotland, UK; ^4^School of Nursing and Midwifery, Keele University, Staffordshire, ST5 5BG, England, UK; ^5^School of Nursing and Midwifery De Montford University, Leicester, LE1 9BH, England, UK; ^6^Department of Health Research, Lancaster University, Lancaster, LA1 4YG, England, UK

##### **Correspondence:** Kevin Brazil (k.brazil@qub.ac.uk)

*Implementation Science 2024*, **19(1)**:O75

**Background**

A goal of implementation science is to expand the use of evidence informed interventions as broadly as possible. ‘Scaling-up’ has clear meaning in implementation science where an intervention designed for one setting is expanded to other health delivery units within the same or very similar settings under which it has been developed. ‘Scaling-out’ is a deliberate effort to deliver an intervention to a new population and /or delivery setting. The present project represents an effort to adapt a proven effective COVID-19 centric advanced care planning (ACP) digital intervention for nursing homes to a community nursing setting. The primary objective of this project includes co-developing an ACP digital education resource for community nurses, patients and their family carers. Facilitators and barriers to implementing the ACP digital intervention will also be identified to develop implementation and evaluation guidelines.

**Methods**

This study employs a 2-phase co-design approach. Phase 1 includes four co-design workshops to seek recommendations from nurses, patients and family carers about content and design of the ACP community digital intervention. We also conducted interviews with a subset of patients, family carers and community nurses to explore experiences of ACP and decision support needs. Phase 2 will include the development of the ACP digital intervention, engaging with community nurses and patients/family carers to complete and evaluate the intervention and its impact.

**Results**

At the time of the conference Phase 1 of the project will be complete. Strategies that represent participatory adaptation of the ACP digital intervention will be reviewed on their merit for applying ‘scale out’ evolution.

**Conclusion**

Rapid deployment of effective interventions to populations experiencing service disparity requires methodological options that is underpinned with an ecological and social perspective.

**Trial Registration:** Not applicable

**Consent to publish:** Yes

## P76: Access to healthcare in Covid-19: evidence from a specialist acute service

### Rona Inniss^1,2^, Natalie Smith^1^, Susie M.D. Henley^1^

#### ^1^Neurofibromatosis Centre, Guy's Hospital, London, SE1 9RT, UK; ^2^Florence Nightingale Faculty of Nursing, Midwifery & Palliative Care, King's College London, London, WC2R 2LS, UK

##### **Correspondence:** Rona Inniss (r.inniss@gstt.nhs.uk)

*Implementation Science 2024*, **19(1)**:P76

**Background**

The Neurofibromatosis Service provides lifelong care to patients with nerve tumour predisposition syndromes [1,2]. Attendance is typically face-to-face outpatient appointments with consultant neurologists, clinical nurse specialists, physiotherapists, psychologists, and a social worker.

Covid-19 forced a severe reduction in face-to-face appointments, with remote offered instead [3,4]. The NHS long-term sustainability plan [5] aims to avert up to 1/3 of face-to-face consultations by 2029. Evidence from patients who had remote appointments during the pandemic can help us shape the roll-out of this long-term plan.

**Methods**

Paper questionnaires were sent to all patients with an appointment within the same 2-week period during August 2020, 2021, 2022.

Response rates were similar: 26% (32/122, 2020), 22% (23/106, 2021), and 25% (26/104, 2022).

**Results**

In 2020 most patients had an appointment by telephone/video. In 2021 and 2022, less than 25% did.

In 2020 just over half of people wanted their next appointment to be remote. By 2021 / 2022 just under half wanted their next appointment to be remote.

Patients report positive and negative experiences of remote appointments but over all three years the most popular choice was still a face-to-face appointment.

Positive experiences included reduced language barriers and improved accessibility (less time off work / childcare). Negative experiences included lack of access to / confidence with technology, and perceived negative impact on relationship with professional.

**Conclusion**

To meet the UN Sustainable Development Goal of good health and wellbeing [6], and the NHS long-term plan [5] to use technology to reduce face-to-face appointments, our research demonstrates that the UK needs to improve access to and confidence in technology, whilst acknowledging the importance the patient places on the in-person relationship with their health professional.

**Trial Registration:** Non applicable

**Consent to publish:** Yes

**References**

1. NHS Commissioning Board. 2013/14 NHS STANDARD CONTRACT FOR COMPLEX NEUROFIBROMATOSIS TYPE 1 SERVICE (ALL AGES). NHS Commissioning Board; 2013. Report No.: B13/S(HSS)/a.
2. NHS Commissioning Board. 2013/14 NHS STANDARD CONTRACT FOR NEUROFIBROMATOSIS TYPE 2 SERVICE (All AGES). NHS Commissioning Board; 2013. Report No.: B13/S(HSS)/b.
3. Thorlby R, Fraser C, Gardner T. Non-COVID-19 NHS care during the pandemic: Activity trends for key NHS services in England [Internet]. The Health Foundation; 2020 Dec [cited 2023 Mar 1]. Available from: https://www.health.org.uk/news-and-comment/charts-and-infographics/non-covid-19-nhs-care-during-the-pandemic
4. Vas V, North S, Rua T, Chilton D, Cashman M, Malhotra B, et al. Delivering outpatient virtual clinics during the COVID-19 pandemic: early evaluation of clinicians’ experiences. BMJ Open Qual. 2022 Jan;11(1):e001313.
5. NHS. The NHS long term plan [Internet]. NHS; 2019. Available from: https://www.longtermplan.nhs.uk/
6. The United Nations Department of Economic and Social Affairs. The Sustainable Development Goals Report 2023: Special Edition. S.l.: United Nations; 2023.

## O77: Investigating a role for implementation science in Irish national environmental policy

### Cáit Ní Chorcora, John O’Neill

#### Institute of Public Administration, 57-61 Lansdowne Road, Dublin 4, D04 TC62, Ireland

##### **Correspondence:** Cáit Ní Chorcora (cnichorcora@ipa.ie)

*Implementation Science 2024*, **19(1)**:O77

**Background**

The mission of the Institute of Public Administration (IPA) in Ireland is to assist the public service with the challenges they face across governance and implementation. This research is looking to address the challenge faced by Ireland’s Environmental Protection Agency (EPA) in unlocking implementation of key polices/programmes at national and local level.

Specifically, the research aims to assess the potential of applying implementation science in wider policy domains (i.e. environmental policy) so as to facilitate better policy coherence and implementation in the fields of environmental research and climate change.

**Method**

The initial element of this research is a comprehensive review of the implementation science literature, focusing on clearly defined areas within health and social care sectors, but also covering wider policy implementation and building on the work by Hering (2018) in assessing relevance of concepts, tools and approaches that are transferable to other sectors such as environmental policy.

The second step will involve consideration of relevant implementation science frameworks for direct applicability in policy areas which are well established (climate adaptation) but also where policy development is still evolving (land use).

**Results**

Our key findings to date include:

- A wide spectrum of approaches to implementation science identified - from the very controlled and confined environment of a fixed community response (i.e. drug intervention scenarios) to approaches where wider policy decisions need to be considered at national/regional or local levels.
- Within this wide spectrum outlined above, it becomes more challenging to define exact applicability of implementation science frameworks when encountering more general evidence for policy considerations.

**Conclusion**

Hybrid possibilities exist to apply implementation science across other disciplines/sectors, such as the environment. Within this context, potential exists to facilitate more efficient and effective public administration processes, thus potentially creating far-reaching benefits for wider society in complex policy areas such as climate.

**Trial Registration:** Not applicable

**Consent to publish:** Not applicable

**Reference**

1. Hering, J. Implementation Science for the Environment. Environ Sci Technol. 2018; 15:52

## P78: Costing digital health implementation efforts in hospitals: a qualitative framework analysis of semi-structured interviews

### Thomasina Donovan^1^, Hannah E Carter^1^, Steven M McPhail^1,2^, Bridget Abell^1^

#### ^1^Australian Centre for Health Services Innovation and Centre for Healthcare Transformation, School of Public Health and Social Work, Faculty of Health, Queensland University of Technology, Brisbane, QLD, Australia; ^2^Digital Health and Informatics, Metro South Health, Brisbane, QLD, Australia

##### **Correspondence:** Thomasina Donovan (thomasina.donovan@hdr.qut.edu.au)

*Implementation Science 2024*, **19(1)**:P78

**Background**

Economic evaluations determine the relative value for money of health innovations and are important for decision makers when allocating scarce resources [1]. However, implementation strategies require additional resourcing which is typically not accounted for in published economic evaluations [2, 3]. This study sought to understand current practices for capturing the costs associated with implementing digital health initiatives in hospital settings, where the complexities of technology and systems present unique challenges for implementation efforts.

**Method**

A qualitative study of semi-structured interviews with 16 purposefully sampled experts in implementation science, health economics and/or digital health was conducted. The interview guide was informed by a literature review and was pilot tested. Interviews were digitally recorded and transcribed. A hybrid inductive/deductive framework analysis was conducted using thematic analysis to elicit key concepts related to the research question.

**Results**

Interviews were conducted with eight implementation scientists, six health economists, and eleven digital health specialists. Four participants were experienced in more than one field. Five key themes were elicited from the data: types of costs, why implementation is costed, how to cost implementation, implementation phases, and barriers and enablers to costing implementation. Broadly, interviewees recognised implementation costs as important but only some costs were considered in practice due to inconsistencies in terminology and the perceived ill-defined boundaries of implementation. Implementation costs were typically recorded to support the delivery of high value care. A variety of methods were used to collect and analyse implementation costs in practice. Multidisciplinary collaboration facilitated this process, but the burden of collecting the necessary data was highlighted.

**Conclusion**

Understanding current practices for capturing implementation costs of digital health initiatives provides opportunities to improve practice and progress research in this space. Ongoing research should establish appropriate methodology for costing implementation efforts within digital health, and healthcare settings more broadly.

**Trial Registration:** Not applicable

**Consent to publish:** Not applicable

**References**

1. Drummond MF, Sculpher MJ, Torrance GW, Stoddart GL. Methods for the economic evaluation of healthcare programs. 3 ed. USA: Oxford university press; 2005.
2. Barnett ML, Stadnick NA, Proctor EK, Dopp AR, Saldana L. Moving beyond Aim Three: a need for a transdisciplinary approach to build capacity for economic evaluations in implementation science. Implementation Science Communications. 2021;2(1):133.
3. Roberts SLE, Healey A, Sevdalis N. Use of health economic evaluation in the implementation and improvement science fields—a systematic literature review. Implementation Science. 2019;14(1):72.

## O79: Sustaining and scaling-up best practices to improve nutrition care in Canadian hospitals using a mentor-champion program

### Katherine L. Ford^1^, Celia Laur^2,3^, Roseann Nasser^4^, Rupinder Dhaliwal^5^, Johane P. Allard^6^, Leah Gramlich^7^, Heather H. Keller^1,8^

#### ^1^Department of Kinesiology and Health Sciences, University of Waterloo, Waterloo, Ontario, N2L 3G1, Canada; ^2^Women’s College Hospital Institute for Health System Solutions and Virtual Care (WIHV), Toronto, Ontario, M5S 1B2, Canada; ^3^Institute of Health Policy, Management and Evaluation, University of Toronto, Toronto, Ontario, M5T 3M6, Canada; ^4^Clinical Nutrition Services, Saskatchewan Health Authority, Regina, Saskatchewan, S4P 1C4, Canada; ^5^Canadian Nutrition Society, Kemptville, Ontario, K0G 1J1, Canada; ^6^Department of Nutritional Sciences, University of Toronto, Toronto, Ontario, M5S 1A4, Canada; ^7^Department of Medicine, University of Alberta, Edmonton, Alberta, T6G 2R3, Canada; ^8^Schlegel-UW Research Institute for Aging, Waterloo, Ontario, N2J 0E2, Canada

##### **Correspondence:** Heather H. Keller (hkeller@uwaterloo.ca)

*Implementation Science 2024*, **19(1)**:O79

**Background**

Up to half of Canadians admitted to hospital are malnourished [1]. There is a need to implement, sustain, and scale-up best practices for malnutrition care in Canada. The More-2-Eat project focused on implementing (Phase 1) and sustaining (Phase 2) an evidenced-based nutrition care pathway [2]. Advancing Malnutrition Care (AMC) aims to scale this success across Canada through a mentor-champion program.

**Methods**

More-2-Eat Phase 1 included implementing a nutrition care pathway in 5 hospital units for 12 months. Phase 2 aimed to sustain the improvements in 4 original hospitals and spread to 6 new hospitals over 18 months. The Capability, Opportunity, and Motivation for Behaviour (COM-B) model guided implementation. To scale across Canada, AMC uses a mentor-champion model with Phase 1 and 2 champions becoming AMC mentors that guide new champions. Baseline audits are underway along with COM-B-based experience questionnaires for mentors and champions. Likert scales were used to assess champions’ preliminary confidence and commitment (1:not; 10:very), and understanding (1:low; 10:high) of changing practice.

**Results**

Champions were key to implementation and sustainability of the nutrition care pathway in Phases 1 and 2, and the AMC mentor-champion model shows promise in continuing this impact. To date, AMC has recruited *n*=8 mentors (*n*=6 from Phase 1 and/or 2), and *n*=8 new champions, from 3 provinces across Canada. Preliminary results found that champions felt confident (mean±SD: 7±1) in their role and committed (9±1) to applying learnings. Understanding of practice change strategies was highest for data collection to track change (8±1) and lowest for changing behaviour (6±2). All champions had experience working with teams to make unit improvements.

**Conclusion**

Champions are confident and committed to changing practice. AMC shows promise in continuing to support sustainable implementation of a nutrition care pathway in Canadian hospitals using a mentor-champion model. Audits and experience surveys will monitor impact.

**Trial Registration:** Not applicable

**Consent to publish:** Not applicable

**References**

1. Allard JP, Keller H, Jeejeebhoy KN, et al. Malnutrition at Hospital Admission-Contributors and Effect on Length of Stay: A Prospective Cohort Study From the Canadian Malnutrition Task Force. JPEN J Parenter Enteral Nutr. 2016;40(4):487-97.
2. Keller H, Laur C, Valaitis R, et al. More-2-Eat: Evaluation Protocol of a Multi-site Implementation of the Integrated Nutrition Pathway for Acute Care. BMC Nutr. 2017;3:13.

## P80: Culturally-appropriate end-of-life care: do and don’t review findings to inform healthcare and other service providers

### Donna M. Wilson^1,2^, Begoña Errasti-Ibarrondo^3^, Brooklyn Grainger^1^, Jean Triscott^1^

#### ^1^University of Alberta, Edmonton, Canada; ^2^University of Navarra, Limerick, Ireland; ^3^ University of Navarra, Pamplona, Spain

##### **Correspondence:** Donna M. Wilson (donna.wilson@ualberta.ca)

*Implementation Science 2024*, **19(1)**:P80

**Background**

End-of-life (EOL) care practices vary considerably between cultures, based on social and religious norms or taboos. As more highly diverse people immigrate to Canada and to other countries, it becomes increasingly important for family physicians, nurses, and many other providers to plan for and provide culturally-appropriate EOL care. What happens before, during, and after dead is extremely important to family members, terminally-ill people, and every society.

**Method**

A scoping review of grey and published material identified preferred practices and also practices to avoid as death nears, at the time of death, and following death for 10 different cultural groups who are relatively new immigrants to Canada: The Philippines, India, China/Hong Kong, Pakistan, Vietnam, Mexico, Korea, Nigeria, Ethiopia, and Lebanon.

**Results**

Significant differences were noted across these groups, often related to religious or spiritual beliefs and social customs. For example, people from Muslim cultures recite verses from the Holy Koran or have it read to them when dying; however, people who are not Muslim should not read this holy text to the dying person. Openly talking about death and dying is another subject that varied between cultural groups, with this related to social norms. It is taboo to talk about death for 8/10 groups; yet open conversations about death and dying is an accepted and encouraged practice in Mexico and Pakistan. Some similarities were noted across all 10 groups, including the importance of involving family members in EOL decision-making and enabling them to care for their dying loved one.

**Conclusion**

Although there were some similarities, many differences were noted. Case-by-case individualized care may be essential for appropriate EOL care, as EOL practices not only differ by culture, but they also can change over time and they can also vary between families.

**Trial Registration:** Not applicable

**Consent to publish:** Not applicable

## O83: Causal loop diagramming to model, tailor, and test sustainment strategies in multi-level, cross-context implementation efforts

### Erika L. Crable^1,2,3^, Thomas Engell^4^, Ryan Kenneally^1,2^, Teresa Lind^2,5^, Gregory A. Aarons^1,2,3^

#### ^1^Department of Psychiatry, University of California San Diego, La Jolla, CA, 92093, USA; ^2^Child and Adolescent Services Research Center, San Diego, CA, 92123, USA; ^3^UC San Diego ACTRI Dissemination and Implementation Science Center, La Jolla, CA, 92093, USA; ^4^Centre for Child and Adolescent Mental Health, Eastern and Southern Norway, Gullhaugveien 1-3, 0484, Oslo, NOR; ^5^Department of Child and Family Development, San Diego State University, San Diego, CA, 92182, USA

##### **Correspondence:** Erika L. Crable (ecrable@health.ucsd.edu)

*Implementation Science 2024*, **19(1)**:O83

**Background**

Approaches to tailor/test sustainment strategies are needed to ensure that service delivery and population health benefits gained during implementation persist over time [1]. Causal loop diagramming (CLD) is a mixed methods, systems science approach to model causal relationships and feedback loops in complex dynamic health systems [2]. This presentation describes CLD’s utility for understanding complex health systems interrelationships that influence implementation and sustainment. CLD methods are illustrated using a National Institutes of Health-funded study that aims to identify causal relationships critical to successful implementation and sustainment of a quality assurance tool (Lyssn) and evidence-based practice (motivational interviewing) for substance use treatment across a statewide behavioral health system in the U.S.

**Methods**

The Exploration, Preparation, Implementation, Sustainment (EPIS) framework guided identification of multi-level outer (state government, service system) and inner (provider organization/clinic) system variables (e.g., agencies/organizations, multi-level actors, competing priorities, policies, money) and their causal interrelationships across implementation phases (Figure 1) [3]. Variable data for the CLD was generated by surveys, qualitative interviews, and document review. Member checking with policy, payor, and provider partners aided in confirming or adjusting causal relationships.

**Results**

CLD revealed reinforcing causal relationships for sustainment within the inner context. However, system dynamics across outer-inner contexts balanced the effects on sustainment in the inner context. The CLD revealed potential bridging factors to support inner-outer context alignment and sustainment [4] and were refined with systems partners.

**Conclusion**

Future system dynamics simulations will test model behavior over time and optimize strategies for sustainment [5]. CLD is a useful mixed methods approach to design sustainment strategies across EPIS phases.

**Trial Registration:** ClinicalTrials.gov identifier: NCT05344534

**Consent to Publish**: N/A no patient/participant level data are presented

**References**

1. Powell BJ, Beidas RS, Lewis CC, Aarons GA, McMillen JC, Proctor EK, Mandell DS. Methods to improve the selection and tailoring of implementation strategies. *J Behav Health Serv Res*. 2017;44(2):177-194.
2. Littlejohns LB, Hill C, Neudorf C. Diverse approaches to creating and using causal loop diagrams in public health research: recommendations from a scoping review. *Public health reviews.* 2021;42:1604352.
3. Crable EL, Lengnick-Hall R, Stadnick NA, Moullin JC, Aarons GA. Where is “policy” in dissemination and implementation science? Recommendations to advance theories, models, and frameworks: EPIS as a case example. *Implement Sci.* 2022;17:80.
4. Lengnick-Hall R, Stadnick NA, Dickson KS, Moullin JC, Aarons GA. Forms and functions of bridging factors: specifying the dynamic links between outer and inner contexts during implementation and sustainment. *Implement Sci.* 2021;16(1):34.
5. Zimmerman L, Lounsbury DW, Rosen CS, Kimerling R, Trafton JA, Lindley SE. Participatory system dynamics modeling: increasing stakeholder engagement and precision to improve implementation planning in systems. *Adm Policy Ment Health.* 2016;43:834–849.

**Fig. 1 (Abstract O83).**
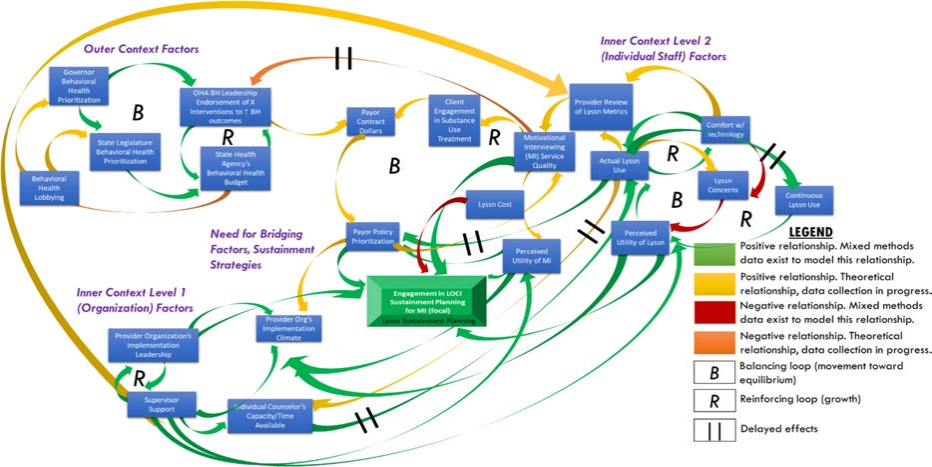
Causal loop diagram identifying relationships between outer and inner context factors/entities necessary for sustainment across implementation phases (in progress)

## P84: NICO: narrative intervention for long COVID pilot feasibility and acceptability study

### Rachel Johnson-Koenke^1,2^, Emma Baker^1^, Jacqueline Jones^1^

#### ^1^University of Colorado, College of Nursing, Aurora, CO, USA; ^2^Department of Veteran Affairs, Eastern Colorado Healthcare System, Aurora, CO, USA

##### **Correspondence:** Rachel Johnson-Koenke (rachel.a.johnson@cuanschutz.edu)

*Implementation Science 2024*, **19(1)**:P84

**Background**

People living with Long COVID frequently experience high symptom burden and trouble with activities of daily living (ADLs)[1]. COVID impacted not only those people living with Long COVID but also put further stress on the mental health system in the US [2,3]. The NICO research study aimed to establish the acceptability and feasibility of implementing an asynchronous narrative intervention for people living with Long COVID.

**Materials and methods**

People with self-reported Long COVID were recruited through social media. Measures were administered at both baseline and 3 months. Program satisfaction semi-structured interviews were conducted with intervention participants. Results were analyzed using conventional content analysis. Descriptive statistics were also used to describe the population and measures.

**Results**

Seventeen study participants consented and enrolled, while eleven completed the intervention (65%). Multiple participants reported that they enjoyed the asynchronous program because it allowed them to engage with it when they had time (Table 1). Results suggest NICO is feasible and acceptable.

**Conclusions**

The NICO research study provides evidence to support the feasibility and acceptability of this asynchronous narrative intervention for people living with chronic illnesses like Long COVID. Many chronic illnesses impact a person’s ability to engage with traditional in-person talk therapy. Combined with limited mental health availability, flexible mental health intervention implementation will be an essential part of helping the increasing number of people living with chronic illnesses. Additional research is needed to refine and implement the NICO intervention to help the many people living with Long COVID and other life-limiting chronic illnesses.

**Trial Registration:** NCT06091293

**Consent to publish:** Yes

**References**

1. Vanichkachorn G, Newcomb R, Cowl CT, Murad MH, Breeher L, Miller S, et al. Post COVID-19 Syndrome (Long Haul Syndrome): Description of a Multidisciplinary Clinic at the Mayo Clinic and Characteristics of the Initial Patient Cohort. In: Mayo Clinic Proceedings. Elsevier; 2021.
2. Fiorillo A, Gorwood P. The consequences of the COVID-19 pandemic on mental health and implications for clinical practice. Eur Psychiatry. 2020;63(1).
3. Usher K, Durkin J, Bhullar N. The COVID‐19 pandemic and mental health impacts. Int J Ment Health Nurs. 2020;29(3):315.

**Table 1 (Abstract P84):** Instrument table

Instrument	Baseline	3 months
Generalized Anxiety Disorder 7 item scale (GAD-7) assessment	9.6 (mild to moderate anxiety)	4.1 (minimal anxiety)
Personal Health Questionnaire (PHQ9)	9.3 (high end of mild depressive symptoms)	5.5 (low end of mild depressive symptoms)
Program Satisfaction Interview		All participants reported satisfaction with the program and that it was helpful (*n*=11)

## O85: Findings from the health champions study

### Julie Williams^1,10^, Ray McGrath^2,10^, Karen Ang^2,10^, Ioannis Bakolis^3^, Andy Healey^1,4^, Jorge Arias de la Torre^1,5,6,7^, Isabel Mdudu^2^, Fiona Gaughran^2,8^, Euan Sadler^9^, Mariana Pinto de Costa^2^, Errol Green^2^, Natalia Stepan^10^, Gracie Tredget^2,10^, Zarnie Khadjesari^11^, Sean Cross^2,10^, Nick Sevdalis^1^

#### ^1^Centre for Implementation Science, King’s Collge London, SE5 8AF, UK; ^2^South London and Maudsley NHS Foundation Trust, London, SE5 8AZ, UK; ^3^Department of Biostatistics and Health Informatics, Institute of Psychiatry, Psychology and Neuroscience, King’s College London, London, SE5 8AF, UK; ^4^Kings Health Economics, Health Service and Population Research Department, Institute of Psychiatry, Psychology and Neuroscience, King's College London, London, SE5 8AF, UK; ^5^Care for Long Term Conditions Research Division. Florence Nightingale Faculty of Nursing, Midwifery & Palliative Care, King’s College London, London, UK; ^6^CIBER Epidemiology and Public Health (CIBERESP). Barcelona, Spain ^7^ Institute of Biomedicine (IBIOMED), Universidad de León. León, Spain; ^8^Psychosis Studies, Institute of Psychiatry, Psychology and Neuroscience, King's College London, London, UK; ^9^Department of Nursing, Midwifery and Health, School of Health Sciences, Faculty of Environmental and Life Sciences, University of Southampton, Southampton, UK; ^10^King’s Health Partners Mind and Body Programme, Ground Floor, Counting House, Guy’s Hospital St Thomas St, London, UK; ^11^Behavioural and Implementation Science (BIS) research group, School of Health Sciences, University of East Anglia, Norwich Research Park, Norwich, UK

##### **Correspondence:** Julie Williams (julie.williams@kcl.ac.uk)

*Implementation Science 2024*, **19(1)**:O85

**Background**

People with severe mental illness (SMI) such as schizophrenia experience inequalities with their physical health including having more physical health comorbidities than the general population. One way to address this to provide individual support for people. Volunteers have been shown to be able to provide support and bring a valued perspective to supporting people with SMI. In this presentation we report the findings on the implementation of a feasibility hybrid trial of an intervention called ‘Health Champions’ where volunteers supported individuals with SMI with their physical health.

**Method**

The study was a feasibility randomised Hybrid II trial in which Health Champions provided one to one support for up to nine months. We assessed clinical, implementation economic effectiveness.

This presentation will focus on implementation effectiveness. To assess this, we conducted interviews with participants and Health Champions at the end of the intervention to understand their experience of the intervention and to evaluate the implementation challenges. We assessed acceptability, feasibility, appropriateness, fidelity, barriers and facilitators and unintended consequences. We used thematic analysis to analyse the data and are mapping this to the Consolidated Framework of Implementation Research (CFIR) v2 to understand the data.

**Results**

We recruited 48 participants-27 in the intervention arm and 21 in the control arm. We interviewed 18 participants and 18 Health Champions. Overall participants and Health Champions found the intervention acceptable, feasible and appropriate. Facilitators for participants included the relationship they built with the Health Champion. Barriers included the impact of the COVID pandemic. The mapping to the CFIR will be discussed in the presentation.

**Conclusion**

We were able to implement the intervention and most participants and Health Champions considered it acceptable, feasible and appropriate. We have a better understanding of the implementation challenges and how these can be addressed.

**Trial Registration:** NCT04124744

**Consent to publish:** This has been given

## P87: Evaluating the implementation of a national programme for transforming mental health in schools across south west London: the Children and Young People (CYP) emotional wellbeing in schools programme

### Helen Sheldon, Phoebe Blackney, Lydia Davies, Andrew Walker

#### Health Innovation Network, Floor 10 Becket House, 1 Lambeth Palace Rd, London SE1 7EU

##### **Correspondence:** Helen Sheldon (helen.sheldon3@nhs.net)

*Implementation Science 2024*, **19(1)**:P87

**Background**

In 2017, the UK Government provided a vision and funding for the transformation of mental health (MH) for children and young people (CYP)[1]. South West London (SWL) secured funding to develop and implement a transformation programme. The creation of school clusters with a Mental Health Support Team (MHST), is a key feature of the programme. MHSTs deliver targeted evidence-based interventions in schools/colleges to CYP, their parents, and staff. The programme aims to support schools/colleges develop a Whole School Approach (WSA)[2] to improving emotional wellbeing. The aim was to understand the implementation and impacts of the programme. Here we report the case study element only.

**Method**

A case study approach was used with four school clusters (*n*=51 schools/colleges) comprising interviews/focus groups (*n*=196), surveys (*n*=226), school cluster meetings (*n*=8). Perspectives were captured from school/college staff, MH service providers, CYP and parents/carers. Staff from schools not involved in the programme were interviewed (*n*=8) to provide a counterfactual. The eight WSA principles were used as an analytical framework.

**Results**

There is evidence of positive change associated with the development and implementation of interventions across the eight WSA domains. There has been increased activity around emotional wellbeing for CYP in schools/colleges, especially interventions delivered by MHSTs, access to self-help resources, and direct support via an online platform. Factors influencing successful implementation functioned at two levels: (1) Schools clusters - additional resources, leadership and workforce development, relational connections; and (2) System - effective governance, leadership, partnerships, national funding. Tensions between the different programme levels linked to priorities and autonomy and differences between education and health sectors (e.g. culture, priorities) impeded implementation.

**Conclusion**

Implementation of a complex system-wide programme has improved emotional wellbeing provision for the whole school community across SWL. Factors operating at multiple levels – school, school clusters and the system – interacted to influence implementation.

**Trial Registration:** Not applicable

**Consent to publish:** Not applicable

**References**

1. Public Health England. *Promoting children and young people’s mental health and wellbeing: A whole school or college approach.* 2021. https://assets.publishing.service.gov.uk/government/uploads/system/uploads/attachment_data/file/1020249/Promoting_children_and_young_people_s_mental_health_and_wellbeing.pdf [Accessed 4th June 2023].
2. HM Government. *Government Response to the Consultation on Transforming Children and Young People’s Mental Health Provision: a Green Paper and Next Steps*. 2018. https://assets.publishing.service.gov.uk/government/uploads/system/uploads/attachment_data/file/728892/government-response-to-consultation-on-transforming-children-and-young-peoples-mental-health.pdf [Accessed 4th June 2023].

## P88: Evaluating the early implementation of digitally enabled virtual wards: a case study approach

### Camille Aznar

#### Health Innovation Network, London, UK

##### **Correspondence:** Camille Aznar (camille.aznar@nhs.net)

*Implementation Science 2024*, **19(1)**:P88

**Background**

Virtual wards (VWs) support patients to receive the acute care, monitoring and treatment in their own home, which would otherwise be provided in hospital. The rapid implementation of VWs was a direct response to the pandemic; however, there is an ambition in England for the expansion of the model i.e. 40–50 ‘virtual ward beds’ per 100,000 population by December 2023[1]. There still is limited evidence about all aspects of VWs[1,2]. This evaluation explores the early implementation of VWs in 2021-22.

**Methods**

A mixed-methods case study approach was used of three NHS VWs across South-West London (SWL) using in-depth interviews with clinical staff in/working directly with VWs (*n*=14), patients admitted and treated on VW (*n*=14), documentation, and routinely collected demographic, activity and outcome data.

**Results**

VWs were used for a range of conditions beyond COVID-19 (e.g. exacerbation of a long-term condition). Patients tended to >65 years old, white and female. Although discharge outcomes varied between the VW models, patients across the three services were able to be cared for at home. Patients felt they were being kept out of hospital whilst receiving the same standard of care as they would in a hospital environment. Clinical VW staff highlighted positive experiences of working on the ward.

Factors influencing the successful implementation of VW models were:

- Offering continuous monitoring to all patients
- Referring a small but targeted cohort of patients
- Developing established clinical and referral pathways
- In-reach virtual ward staff based in acute settings
- Strengthening relationships with acute trust and VW staff
- Building multi-disciplinary teams
- Clear clinical governance arrangements in place

**Conclusion**

Early evidence in SWL shows VWs have been successfully implemented and expanded to care pathways beyond patients with COVID-19 with patients being treated safely and comfortably at home. Common factors enabling implementation were identified across three different VWs models.

**Trial Registration:** Not applicable

**Consent to publish:** Not applicable

**References**

1. Enablers for success: virtual wards including hospital at home [Internet]. NHS England; 2022. Available from: https://www.england.nhs.uk/wp-content/uploads/2022/04/B1382_supporting-information-for-integrated-care-system-leads_enablers-for-success_virtual-wards-including-hos.pdf
2. Best J. The virtual wards aiming to ease hospital pressures. BMJ. 2022 Jul 6;378:o1603.

## P90: 2YoungLives: changing mindsets with a million conversations

### Mangenda Kamara^1,2^, Lucy November^3^, Prince T Williams^4^, Philemon Kamara^4^, Jane Sandall^3^, Cristina Fernandez Turienzo^3^

#### ^1^Welbodi Partnership, Freetown, Sierra Leone; ^2^University of Sierra Leone, Faculty of Social Sciences, Freetown, Sierra Leone; ^3^King’s College London, Department of Women and Children’s Health, UK, SE1 7EH; ^4^Lifeline Nehemiah Projects, Freetown, Sierra Leone

##### **Correspondence:** Lucy November (lucy.november@kcl.ac.uk)

*Implementation Science 2024*, **19(1)**:P90

**Background**

2YoungLives is a community-based mentoring scheme for pregnant adolescents in Sierra Leone developed by community-based organisation Lifeline Nehemiah Projects (LNP) [1] after exploring contributing factors to high adolescent maternal mortality [2]. Women are trained to mentor pregnant girls to; start a small business, reconcile with families, take up maternity care and postpartum contraception, breastfeed exclusively, and re-engage in education. A pilot cluster-randomised trial is underway to assess feasibility and implementation strategies in new communities and to inform future scale-up [3].

**Method**

We discuss the strategy for meaningful and comprehensive community engagement and involvement (CEI), a core component of the 2YoungLives programme, essential for scale-up and sustainability. The strategy included three CEI visits to each intervention site: 1) introductions and paying respects to town chiefs; 2) meeting key stakeholders and conducting open community-wide meetings to share local beliefs and voice concerns; 3) identify mentors in collaboration with community stakeholders. Listening, discussing and connecting is imperative to building trusting relationships, mitigating issues which inevitably arise during implementation.

**Results**

Important barriers were raised and discussed (i.e. cultural/ religious, historical, political), and time given for co-development of bespoke solutions. For example, in one community, mentees were reluctant to attend the government health facility for fear of a practice of reporting pregnant under-18s to the police. The LNP team engaged facility staff and community stakeholders, and invited the midwife to attend the monthly 2YoungLives meeting, building a trusting relationship and giving girls confidence to attend. There are many examples of discussions about gender-based issues such as child-marriage or FGM leading to wider socio-cultural changes to attitudes and practices beyond the 2YoungLives intervention.

**Conclusion**

The CEI showcased in this case-study is not a tick-box exercise but a vital component of successful implementation and sustainability with many lessons learned for others implementing complex interventions in similar contexts.

**Trial Registration:** Not applicable

**Consent to publish:** Not applicable

**References**

1. 2YoungLives. Saving lives in Sierra Leone. Available from: https://2younglives.org (Accessed 8 December 2022)
2. November L, Sandall J. ‘Just because she’s young, it doesn’t mean she has to die’: exploring the contributing factors to high maternal mortality in adolescents in Eastern Freetown; a qualitative study. Reproductive Health. 2018 Dec;15(1):1-8.
3. Turienzo CF, November L, Kamara M, Kamara P, Goodhart V, Ridout A, Sam B, Thomas S, Williams PT, Sandall J, Shennan AH; CRIBS Collaborative Group. Innovations to reduce maternal mortality and improve health and wellbeing of adolescent girls and their babies in Sierra Leone. Lancet Child Adolesc Health. 2023 Mar;7(3):151-153.

## P91: Barriers and facilitators to scale-up of hospital-at-home: an observational cohort study protocol

### Stephanie Q Ko^1^, Nick Sevdalis^2^

#### ^1^Division of Advanced Internal Medicine, Department of Medicine, National University Hospital, Singapore; ^2^Centre of Behavioural and Implementation Science Interventions (BISI), Yong Loo Lin School of Medicine, National University of Singapore

##### **Correspondence:** Stephanie Q Ko (stephanie_ko@nuhs.edu.sg)

*Implementation Science 2024*, **19(1)**:P91

**Background**

Hospital-at-home interventions [1] have been shown to be clinically and cost-effective [2–5], and many healthcare systems internationally are investing in scaling-up such interventions [6–9]. However, most existing studies are focusing on how effective the intervention is, rather than how to successfully scale it up. We report a study protocol for a theory-driven investigation of a hospital-at-home intervention. We propose a novel combination of two established implementation science frameworks – the EPIS framework [10,11] and the Scale-Up framework [12] – and apply it to a planned scale-up of a hospital-at-home intervention in Singapore.

**Method**

EPIS offers a useful macro-framework by identifying contextual influences across the phases of Exploration, Preparation, Implementation, and Sustainment. The macro approach of EPIS needs to be further supported by an action-orientated framework of the scale-up process. The Scale-Up framework breaks down scaling-up into 4 phases: set-up, develop the scalable unit, test of scale-up, and go to full-scale.

We will conduct an observational cohort study across 24 months (May 2022 to April 2024) to evaluate the association of outer and inner contextual factors on key implementation outcomes – the volume of patients admitted, operational efficiency and levels of adoption. Statistical process control graphs will be used to examine variation in the implementation outcomes over time. Linear regression will be applied to assess associations of outcomes with contextual factors that are continuous variables; logistic regression will be applied to assess the associations of outcomes with binary/descriptive contextual factors. To supplement these, qualitative methods will be applied using a content analysis of monthly meeting minutes and focus groups of the implementation team to understand and explain the outcomes of the observational cohort study.

**Results:**

NA (study protocol)

**Conclusions**

This study protocol applies implementation frameworks to systematically evaluate the scale-up process and identifying barriers and facilitators towards going to full scale.

**Trial Registration:** Non applicable

**Consent to publish:** Non applicable

**References**

1. Leff B. Defining and disseminating the hospital-at-home model. CMAJ Can Med Assoc J J Assoc Medicale Can. 2009 Jan 20;180(2):156–7.
2. Shepperd S, Iliffe S, Doll HA, Clarke MJ, Kalra L, Wilson AD, et al. Admission avoidance hospital at home. Cochrane Effective Practice and Organisation of Care Group, editor. Cochrane Database Syst Rev [Internet]. 2016 Sep 1 [cited 2021 Jan 20]; Available from: http://doi.wiley.com/10.1002/14651858.CD007491.pub2
3. Gonçalves-Bradley DC, Iliffe S, Doll HA, Broad J, Gladman J, Langhorne P, et al. Early discharge hospital at home. Cochrane Effective Practice and Organisation of Care Group, editor. Cochrane Database Syst Rev [Internet]. 2017 Jun 26 [cited 2021 Jan 20]; Available from: http://doi.wiley.com/10.1002/14651858.CD000356.pub4
4. Caplan GA. A meta-analysis of “hospital in the home”. Med J Aust. 2013;198(4):195–6.
5. Leong MQ, Lim CW, Lai YF. Comparison of Hospital-at-Home models: a systematic review of reviews. BMJ Open. 2021 Jan 1;11(1):e043285.
6. Gorbenko K, Baim-Lance A, Franzosa E, Wurtz H, Schiller G, Masse S, et al. A national qualitative study of Hospital-at-Home implementation under the CMS Acute Hospital Care at Home waiver. J Am Geriatr Soc. 2022 Oct 5;
7. Chong C. Some patients can now be hospitalised at home and be cared for via teleconsultations, home visits. The Straits Times [Internet]. 2022 Sep 29 [cited 2022 Dec 28]; Available from: https://www.straitstimes.com/singapore/some-patients-can-now-be-hospitalised-at-home-and-be-cared-for-via-teleconsultations-home-visits
8. NHS UK. Virtual wards [Internet]. [cited 2023 Jun 3]. Available from: https://www.england.nhs.uk/virtual-wards/
9. Digital Health. Government plans 500% expansion of virtual wards [Internet]. [cited 2023 Mar 6]. Available from: https://www.digitalhealth.net/2023/01/government-plans-500-expansion-of-virtual-wards/
10. Aarons GA, Hurlburt M, Horwitz SM. Advancing a Conceptual Model of Evidence-Based Practice Implementation in Public Service Sectors. Adm Policy Ment Health. 2011;38(1):4–23.
11. Moullin JC, Dickson KS, Stadnick NA, Rabin B, Aarons GA. Systematic review of the Exploration, Preparation, Implementation, Sustainment (EPIS) framework. Implement Sci. 2019 Dec;14(1):1–16.
12. Barker PM, Reid A, Schall MW. A framework for scaling up health interventions: lessons from large-scale improvement initiatives in Africa. Implement Sci IS. 2016 Jan 29;11:12.

## P92: Regulation of infant formula: the role of health policy in dissemination and implementation strategy

### Bartosz Helfer^1,2^

#### ^1^Meta-Research Centre, University of Wroclaw, Wroclaw, Poland; ^2^Institute of Psychology, University of Wroclaw, Wroclaw, Poland

##### **Correspondence:** Bartosz Helfer (bartosz.helfer@gmail.com)

*Implementation Science 2024*, **19(1)**:P92

**Background**

There is an ongoing debate regarding safety, quality, and marketing of infant formula products with many authors calling for a new improved regulatory framework. Health policy plays a key role in the development, dissemination, and implementation of evidence-based practices to address these concerns. This analysis aimed to examine the health policy role in the dissemination and implementation strategy regarding improved regulation of infant formula, using Crable et al.'s [1] recommendations as a guide.

**Methods**

A health policy analysis was conducted to identify the key dimensions of the policy's function and form, examine the nonlinear phases of policy dissemination and implementation, describe the temporal roles of stakeholders, consider policy-relevant outer and inner context adaptations, and identify bridging factors necessary for policy success. The analysis included a review of relevant literature, stakeholder views, and an assessment of existing regulatory frameworks.

**Results**

There is a clear need to strengthen the evidence base for infant formula regulation, enhance transparency in policy development, develop new evidence-based guidelines, establish robust monitoring and enforcement systems, promote public awareness, and facilitate international collaboration. Stakeholders, including healthcare professionals, researchers, manufacturers, consumer advocates, and caregivers should work together to help develop a successful dissemination and implementation strategy for health policy.

**Conclusions**

A comprehensive and evidence-based health policy approach is necessary to fully address the current controversy regarding regulation of infant formula. By applying Crable et al.'s [1] recommendations, health policies can be more effectively disseminated and implemented to ensure better safety, quality, and appropriate marketing of infant formula products. This approach will ultimately contribute to better infant nutrition and public health outcomes.

**Trial Registration:** Non applicable

**Consent to publish:** Non applicable

**Reference**

1. Crable EL, Lengnick-Hall R, Stadnick NA, Moullin JC, Aarons GA. Where is “policy” in dissemination and implementation science? Recommendations to advance theories, models, and frameworks: EPIS as a case example. Implementation Science. 2022 Dec 12;17(1):80.

## O95: Knowledge translation strategies for the sustainability of evidence-based interventions in healthcare: A scoping review

### Rachel Flynn^1,2^, Christine Cassidy^3^, Lauren Dobson^2^, Ian D. Graham^4,5^, Shannon D. Scott^2^

#### ^1^School of Nursing and Midwifery, Brookfield Health Sciences Complex, University College of Cork, College Road Cork, Ireland , T12 AK54; ^2^Faculty of Nursing, Level 3, Edmonton Clinic Health Academy, 11405 87 Avenue, University of Alberta, Edmonton, Alberta Canada, T6G 1C9; ^3^School of Nursing, Room N21, Forrest Bldg., Faculty of Health, Dalhousie University PO Box 15000 5869 University Avenue Halifax NS B3H 4R2; ^4^School of Epidemiology and Public Health, University of Ottawa, 600 Peter Morand Crescent, Ottawa, ON Canada K1G 5Z3; ^5^The Centre for Implementation Research, Ottawa Hospital Research Institute, 501 Smyth Road, Box 241, Ottawa, Ontario K1H 8L6

##### **Correspondence:** Rachel Flynn (rachelflynn@ucc.ie)

*Implementation Science 2024*, **19(1)**:O95

**Background**

This scoping review aimed to consolidate the current evidence on: i) what and how KT strategies are being used for the sustainability of evidence-based interventions (EBIs) in institutional healthcare settings; ii) barriers and facilitators to the use of KT strategies for sustainability; and iii) reported KT implementation outcomes and EBI sustainability outcomes.

**Methods**

We conducted a scoping review of five electronic databases. We included studies that described the use of specific KT strategies to facilitate the sustainability of EBIs (more than 1 year post-implementation). Two reviewers independently screened titles and abstracts, full text papers, and extracted data. We coded KT strategies using the ERIC taxonomy of implementation strategies and barriers/facilitators using the Consolidated Framework for Sustainability. We performed descriptive numerical summaries and a narrative synthesis to analyze results.

**Results**

From the 25 included studies, the most common KT strategies for sustainability of an EBI were train & educate stakeholders (*n*=38) and develop stakeholder interrelationships (*n*=34). Barriers to KT strategy use for EBI sustainability were mostly related to resources (*n*=20). Facilitators to KT strategy use for EBI sustainability were mostly related to the people involved (*n*=28) and design and delivery of the KT strategy (*n*=20). Most studies (*n*=11) did not clearly report whether they used different or the same KT strategies between EBI implementation and EBI sustainability. Seven studies adapted their KT strategies from implementation to sustainability and only two studies reported using a new KT strategy for EBI sustainability.

**Conclusions**

Our review provides insight into a conceptual problem where implementation and sustainability are two discrete activities that occur at separate times. Our findings show we need to consider implementation and sustainability as a continuum and at the start of the EBI implementation select, design and adapt KT strategies across the continuum with this in mind.

**Trial Registration:** Not applicable

**Consent to publish:** Not applicable

## P96: Evaluating the prioritisation and implementation process within the Mental Health Implementation Network (MHIN)

### Blossom Fernandes^1^, Shalini Ahuja^2^, Nick Sevdalis^3^, MHIN Consortium, Annette Boaz^1^

#### ^1^Department of Health Services Research and Policy, Faculty of Public Health and Policy, London School of Hygiene and Tropical Medicine (LSHTM), 15-17 Tavistock Place, London, WC1H 9SH, UK; ^2^The Centre for Implementation Science, Institute of Psychiatry, Psychology & Neuroscience (IoPPN), 16 De Crespigny Park, London, SE5 8AF, UK; ^3^Department of Psychological Medicine, Yong Loo Lin School of Medicine, National University of Singapore

##### **Correspondence:** Blossom Fernandes (blossom.fernandes@lshtm.ac.uk)

*Implementation Science 2024*, **19(1)**:P96

**Background**

A cross-disciplinary consortium called the Mental Health Implementation Network (MHIN) with key stakeholders was established in England in 2020 implementing mental health interventions in six regions of England. Led by local Applied Research Collaborations and their partners. The aim of the present study is twofold: to develop an overarching evaluation strategy for programme level and site level evaluations of MHIN, and b) programme wide evaluations which focuses on the relational work between prioritisation and implementation, including the development of sustainability constructs linked with the implementation support packages at each sites.

**Methods**

The study is underpinned by an embedded mixed method approach [1]. Data collection methods include observations, expert consultations, document analysis, structured questionnaire and semi structured interviews with key stakeholders encompassing the public and local communities, multi-sector health and care providers, commissioners, government, NGO, clinical, managerial, commissioning, academic and other partners. Overall the Exploration, Preparation, Implementation, Sustainment (EPIS) framework [2] is used to understand and support the implementation process at the six delivery sites. Evaluation strategy was developed using expert consultations and document analysis.

**Results**

Data is currently analysed using different qualitative approaches including narrative synthesis and framework analysis. We will share emerging themes from an ongoing analyses; these are centred around the relations between stakeholders, the resources needed for setting up a priority network and regional vs national implication of setting up a network. Further findings include the negotiations recorded at site activities, and local evaluation of the tailored implementation support for the sites.

**Conclusion**

The findings from MHIN stakeholders in this process to support a wide variety of projects and ARC sites will provide insightful information relating to the factors promoting or inhibiting implementation from different stakeholder perspectives. This could be extended beyond the specific project and be useful for implementation researchers and implementation practitioners.

**Trial Registration:** Not applicable

**Consent to publish:** Not applicable

**References**

1. Cresswell JW, Plano Clark VL. Designing and conducting mixed methods research. 2011.
2. Moullin JC, Dickson KS, Stadnick NA, Rabin B, Aarons GA. Systematic review of the exploration, preparation, implementation, sustainment (EPIS) framework. Implementation Science. 2019 Dec;14(1):1-6.

## P98: Co-development workshops as an implementation strategy?

### Leah Bührmann^1^, Sebastian Potthoff^1^, Peter van der Graaf^2^, Tracy Finch^2^

#### ^1^Department of Social Work, Education and Community Wellbeing, Northumbria University, Newcastle upon Tyne, UK; ^2^Department of Nursing, Midwifery and Health, Northumbria University, Newcastle upon Tyne, UK

##### **Correspondence:** Leah Bührmann (leah.buhrmann@northumbria.ac.uk)

*Implementation Science 2024*, **19(1)**:P98

**Background**

Implementing interventions across complex systems, at different levels and between different professional groups and organisations is challenging. Co-design methods have the potential to support implementation of boundary-crossing interventions but are often used implicit. We aimed to make these processes explicit and reflected with stakeholders on the benefits of co-design methods for their own implementation practices.

**Methods**

In this study, an implementation toolkit that supports the integration of health and social care in the Integrated Care System of North East England was co-developed. Regional stakeholders (*n*=13), including health care professionals, service users, and decision makers, were invited to participate in a series of seven co-development workshops. Workshops were conducted according to a systematic intervention development process. After each workshop, participants rated the workshops on five questions ranging from overall satisfaction to how the workshop might have influenced individual practices. The workshops were held online, recorded, and transcribed.

**Results**

The workshops resulted in a series of co-developed materials, including a comprehensive context assessment, a list of local determinants to implementation, a detailed power-interest mapping of key stakeholders for local implementation, planned implementation activities, and best practice examples for implementation. Feedback from participants indicated how useful such workshops are for their own practices. The workshops stimulated the exchange of perspectives among stakeholders from different backgrounds, facilitated the sharing of best practices, and established new collaborations that directly impact workshop participants' daily practice. This being an implicit product of the workshops, it raised the question of how we can best utilise co-development workshops as part of an implementation process.

**Conclusion**

Co-development workshops have the potential to be an explicit implementation strategy. If considered during study planning, such workshops have the potential to build capacity for participant’s individual practices as well as to contribute to an infrastructure that will ultimately support implementation of the co-developed materials in practice.

**Trial Registration:** Not applicable

**Consent to publish:** Not applicable

## O99: Implementation science with an equity lens: optimising the use of normalisation process theory in participatory health research with migrants

### Yuki Seidler^1^, Carl May^2^, Anne MacFarlane^3^

#### ^1^University of Vienna, Department of Political Science, Universitätsstrasse 7,1010 Vienna, Austria; ^2^London School of Hygiene and Tropical Medicine, Department of Health Services Research and Policy, Faculty of Public Health and Policy, Keppel Street, London, WC1E 7HT, UK; ^3^University of Limerick, Health Research Institute, Faculty of Education & Health Sciences, School of Medicine, V94 T9PX, Ireland

##### **Correspondence:** Yuki Seidler (yuki.seidler@univie.ac.at)

*Implementation Science 2024*, **19(1)**:O99

**Background**

Work in the field of implementation science has recently taken up a focus on health equity issues [1]. This paper presents a protocol to advance state of the art, building on pioneering work that integrated Normalisation Process Theory (NPT) with Participatory Learning and Action (PLA) research [2-3]. In an EU funded project (2011-2015), this combined approach was found effective in: 1) addressing the exclusion of migrants in health research, and 2) understanding the implementation processes of using trained interpreters in supporting migrants in European primary healthcare systems [4-6]. It could not conclude, however, if PLA was the optimal method to be integrated with NPT in terms of representation, efficiency and effectiveness. Evidence about comparative merits of PLA *vis a vis* other co-creation methods is required. This paper asks how does a participatory, online Delphi method [7] compare with PLA in NPT informed implementation research.

**Methods**

This is a participatory health research [8] using NPT which provides an empirically verifiable explanation of the mechanisms that motivate and shape implementation processes. It is a comparative, instrumental case study [9] using ‘implementation work to normalise trained interpreters in Austrian healthcare settings’ as the case. Purposive sampling will guide recruitment of community and health sector participants in two regional health authorities. Fieldwork will be informed by literature reviews and involve prospective, parallel use of NPT-PLA (site 1) and NPT-online Delphi (site 2) to investigate and support the development of implementation action plans. A qualitative comparative analysis of the action plans and participants’ experiences will be conducted.

**Results**

This project will generate new knowledge about co-creation methods in theoretically informed implementation research.

**Conclusion**

Findings will inform transdisciplinary participatory approaches and patient-centred and inclusive models of practice in theory-informed implementation science research.

**Trial Registration:** Not applicable

**Consent to publish:** Not applicable

**References**

1.Baumann AA, Cabassa LJ. Reframing implementation science to address inequities in healthcare delivery. BMC Health Serv Res. 2020;20(1):190.
May C, Rapley T, Finch T. Normalization Process Theory. In: Nilsen P, Birken S, editors. International Handbook of Implementation Science. London: Edward Elgar; 2020. p. 144–67.
Chambers R. The origins and practice of participatory rural appraisal. World Development. 1994;22(7):953-69.
MacFarlane A, O'Donnell C, Mair F, O'Reilly-de Brún M, de Brún T, Spiegel W, et al. REsearch into implementation STrategies to support patients of different ORigins and language background in a variety of European primary care settings (RESTORE): study protocol. Implement Sci. 2012;7:111.
O'Reilly-de Brún M, de Brún T, O'Donnell CA, Papadakaki M, Saridaki A, Lionis C, et al. Material practices for meaningful engagement: An analysis of participatory learning and action research techniques for data generation and analysis in a health research partnership. Health Expect. 2018;21(1):159-70.
MacFarlane A, Dowrick C, Gravenhorst K, O'Reilly-de Brún M, de Brún T, van den Muijsenbergh M, et al. Involving migrants in the adaptation of primary care services in a 'newly' diverse urban area in Ireland: The tension between agency and structure. Health Place. 2021;70:102556.
Kezar A, Maxey D. The Delphi technique: an untapped approach of participatory research. International journal of social research methodology. 2016;19(2):143-60.
International Collaboration for Participatory Health Research. Research ICfPH. Position Paper 1: What is Participatory Health Research? . Berlin; 2013.
Yin RK. Case study research and applications: design and methods. Sixth ed. Los Angeles: SAGE; 2018.

## O100: The sustained implementation of interventions to support self-management: a scoping review

### Helen Ross-Blundell^1^, Annette Boaz^2^, Nicola Hancock^3^, Fiona Jones^4^

#### ^1^Faculty of Health, Science, Social Care and Education, Kingston University, Kingston upon Thames, Surrey, UK; ^2^Faculty of Public Health & Policy, London School of Hygiene and Tropical Medicine, London, UK; ^3^School of Health Sciences, University of East Anglia, UK; ^4^Population Health Research Institute, St George’s University of London, London, UK

##### **Correspondence:** Helen Ross-Blundell (k2035529@kingston.ac.uk)

*Implementation Science 2024*, **19(1)**:O100

**Background**

Sustained use of an evidence-based intervention ensures maximal long-term and ongoing benefits for patients and services from the initial investment of time and money [1,2]. Supporting self-management has been recognised as an essential component of healthcare, with an established evidence-base demonstrating effectiveness in improving clinical outcomes and patient experience [3,4]. Despite a growing body of evidence exploring the implementation of self-management interventions, there is a paucity in research around the sustainability of these interventions [5]. This scoping review aims to identify and map the evidence available on the sustainability of self-management interventions implemented within adult healthcare services.

**Methods**

A database search was run in Medline, Embase, CINAHL, AMED and PsycInfo, alongside a grey literature search. The search terms were kept deliberately broad due to recognised difficulties in defining the key concepts of self-management and sustainability. Studies considering the long-term effectiveness of interventions were initially included. Multiple stages of selection and extraction enabled detailed exploration of how sustainability is captured and considered.

**Results**

578 articles met the broad inclusion criteria, with 483 of these reporting on the long-term effectiveness of interventions. The remaining 95 articles considered the sustained implementation of a self-management intervention. The depth to which sustainability was included or reported on varied greatly. Only a small proportion featured sustainability as the primary focus of the study, providing details as to the evaluation methods or determinants of sustainability. A detailed analysis of the findings will be presented at the conference.

**Conclusion**

The review found a predominance of research focusing on long-term effectiveness and clinical outcomes rather than sustained implementation. A detailed analysis of the papers focussed on implementation identifies barriers and facilitators to sustainability, highlights gaps in the literature and provides a base for future evaluations to work from.

**Trial Registration:** Not applicable

**Consent to publish:** Not applicable

**References**

1. Greenhalgh T, Robert G, Macfarlane F, Bate P, Kyriakidou O. Diffusion of Innovations in Service Organizations: Systematic Review and Recommendations. Milbank Q. 2004 Dec;82(4):581–629.
2. Wiltsey Stirman S, Kimberly J, Cook N, Calloway A, Castro F, Charns M. The sustainability of new programs and innovations: a review of the empirical literature and recommendations for future research. Implement Sci. 2012 Mar 14;7(1):17.
3. de Silva D. Helping people help themselves. London: The Health Foundation; 2011.
4. Taylor SJ, Pinnock H, Epiphaniou E, Pearce G, Parke HL, Schwappach A, et al. A rapid synthesis of the evidence on interventions supporting self-management for people with long-term conditions: PRISMS – Practical systematic RevIew of Self-Management Support for long-term conditions. Health Serv Deliv Res. 2014 Dec;2(53):1–580.
5. Ahmad DN, Ellins DJ, Krelle H, Lawrie M. Person-centred care: from ideas to action. London: The Health Foundation; 2014.

